# Leaf Na^+^ effects and multi-trait GWAS point to salt exclusion as the key mechanism for reproductive stage salinity tolerance in rice

**DOI:** 10.1093/aob/mcae227

**Published:** 2024-12-28

**Authors:** Marjorie P de Ocampo, Bui Phuoc Tam, James A Egdane, Dmytro Chebotarov, Kazuyuki Doi, Akira Yamauchi, Abdelbagi M Ismail, Amelia Henry, Shiro Mitsuya

**Affiliations:** International Rice Research Institute, Pili Drive, Los Baños, Laguna 4031,Philippines; Loc Troi Agricultural Research Institute, Hoa Tan Village, Dinh Thanh Commune, Thoai Son District, An Giang Province, Vietnam; International Rice Research Institute, Pili Drive, Los Baños, Laguna 4031,Philippines; International Rice Research Institute, Pili Drive, Los Baños, Laguna 4031,Philippines; Graduate School of Bioagricultural Sciences, Nagoya University, Chikusa, Nagoya 464-8601, Japan; Graduate School of Bioagricultural Sciences, Nagoya University, Chikusa, Nagoya 464-8601, Japan; International Rice Research Institute, Pili Drive, Los Baños, Laguna 4031,Philippines; International Rice Research Institute, Pili Drive, Los Baños, Laguna 4031,Philippines; Graduate School of Bioagricultural Sciences, Nagoya University, Chikusa, Nagoya 464-8601, Japan

**Keywords:** Favourable haplotypes, genome-wide association study (GWAS), reproductive stage, rice, salinity tolerance, trimming leaves

## Abstract

**Background and Aims:**

Since salinity stress may occur across stages of rice (*Oryza sativa*) crop growth, understanding the effects of salinity at the reproductive stage is important, although it has been much less studied than at the seedling stage.

**Methods:**

Lines from the Rice Diversity Panel 1 (RDP1) and the 3000 Rice Genomes (3KRG) were used to screen morphological and physiological traits, map loci controlling salinity tolerance through genome-wide association studies (GWAS), and identify favourable haplotypes associated with reproductive stage salinity tolerance.

**Key Results:**

Salt exclusion was identified as the key tolerance mechanism in this study, based on reduced panicle length as flag leaf Na^+^ increased and a lack of effect of trimming the leaves on genotypic rankings in the salinity treatment. Since larger biomass showed a negative effect on the number of filled grains in multiple experiments, future studies should investigate the effect of whole-plant transpiration levels on salt uptake. In addition to genome-wide significant peaks identified in the single-trait GWAS analyses, six loci showed colocations for multiple traits across experiments. Among these colocating loci, three candidate loci that exhibited favourable haplotypes were also characterized to be involved in co-expression networks, among which apoplast and cell wall functions had been annotated, further highlighting the role of salt exclusion.

**Conclusion:**

The loci identified here could be considered as potential sources for improving reproductive stage salinity tolerance in rice.

## INTRODUCTION

Rice plants show different stress tolerance levels depending on the growth stage of the crop at which the stress occurs. In the case of salinity, rice is relatively tolerant during germination, active tillering and towards maturity, but sensitive during the early seedling and reproductive stages (Pearson and Bernstein, 1959; Akbar *et al.*, 1972; Ismail *et al.*, 2007; Walia *et al.*, 2007). The physiological bases of salinity tolerance during the early seedling stage are fairly well established: key traits include salt exclusion, or the uptake of Na^+^, which is restricted either at the root level or from active tissue; compartmentation of ions in structural and older tissues; vigorous growth and developmental mechanisms wherein responses to salinity stress are altered; and tissue tolerance, or tolerance of Na^+^ entering the cell at relatively higher concentrations (Yeo and Flowers, 1986; Yeo *et al.*, 1990; Peng and Ismail, 2004; Moradi and Ismail, 2007). However, relatively less is known about the important mechanisms associated with tolerance at the reproductive stage, which appear to be different from those conferring tolerance at the seedling stage (Moradi *et al.*, 2003). Previous studies have reported either exclusion or leaf-to-leaf compartmentation as key salt tolerance mechanisms at the reproductive stage (Yeo and Flowers, 1986; Moradi *et al.*, 2003), although these studies on reproductive stage salinity tolerance in rice were conducted on small sets of lines. Furthermore, different methods for reproductive stage salinity screening have been used in previous studies, including those in which the leaves were trimmed (which may affect factors such as light penetration, hormonal responses, potential for salt compartmentation to existing biomass, etc.). Therefore, evaluation of larger sets of lines and direct comparison of the different screening methods may reveal additional insights in terms of genetic variation.

Publicly available germplasm through the Genebank at the International Rice Research Institute (IRRI), along with the availability of the genomic datasets, has provided a platform for conducting genome-wide association studies (GWAS). This includes the Rice Diversity Panel 1 (RDP1; McCouch *et al.*, 2010; Zhao *et al.*, 2011) and the 3000 Rice Genomes (3KRG; Li *et al.*, 2014; The 3000 Rice Genomes Project, 2014), which serve as a foundation for discovery of novel alleles for important rice phenotypes using various bioinformatics and/or genetic approaches. In this study we explore the extent of genetic variation in salinity tolerance among diverse rice lines at the reproductive stage. We characterized a range of physiological and agronomic traits to help pinpoint key salinity tolerance mechanisms at the reproductive stage. GWAS was performed using both the RDP1 panel and two subsets from the 3KRG to map loci controlling salinity tolerance and to identify favourable haplotypes that may be used to develop improved rice lines with salinity tolerance at the reproductive stage, as well as to identify candidate genes and their co-expression networks whose functions could add confidence to the key salt tolerance mechanisms observed from the physiological measurements.

## MATERIALS AND METHODS

Four experiments were conducted in this study, three using diversity panels but with two different phenotyping protocols and a fourth experiment using both phenotyping protocols. The RDP1 was used in the first experiment and two subsets from the 3KRG (first batch and second batch) were used in the second and third experiments. The fourth experiment was conducted using selected lines from the first three experiments.

### Plant materials

The plant material used in this study consisted of 324 out of 413 lines from the RDP1 (Supplementary Data Table S1) and two subsets of ~100 lines each from the 3KRG (Supplementary Data Table S2). For the 3KRG subsets, these were lines that stood out in ongoing studies on reproductive stage drought, which we then aimed to evaluate under reproductive stage salinity. Pokkali, a salinity-tolerant landrace from India, and NSIC Rc222 (IRRI 154), a salinity-sensitive, high-yielding variety from the Philippines, were used as checks in all experiments.

### Growth conditions for reproductive stage salinity screening

All experiments were carried out in a greenhouse at IRRI under natural light using 1-L soil-filled pots filled with fertilized soil (50 N, 25 P and 25 K mg kg^−1^). Seeds were placed in an oven for 5 d at 50 °C for dormancy breaking, after which 20 seeds per line were put in Petri dishes with moistened paper towels and were incubated at 32 °C for 48 h. Four or five pre-germinated seeds were placed on the soil surface of each pot in a split plot design wherein treatment was the main plot and lines were the subplot, with three replications. The subsequent experimental protocols used for the RDP1 and the 3KRG subsets were slightly different, the main difference being the leaf trimming that was implemented in the 3KRG experiments. Since both methods (trimming/not trimming) have been used in previous reproductive stage salinity studies, and given their potentially different effects on genotypic rankings, we aimed to compare them directly in a final experiment. For the RDP1 experiment, the pots were kept in concrete tanks filled with tap water maintained at 3 cm below the soil surface. Two weeks after seeding, seedlings were thinned to three per pot and the water level was raised to ~1–2 cm above the soil surface. To impose the reproductive stage salinity treatment in the RDP1 experiment, water was siphoned when the seedlings were 21 d old and the pots were drained for 12 h. The tanks were then flooded with either tap water (control) or saline solution, with an electrical conductivity (EC) of 5 dS m^−1^ using NaCl for 3 d, which was then increased progressively to an EC of 10 dS m^−1^ until maturity. The pots were kept flooded for the duration of the experiment, and the EC of the saline solution was monitored daily and adjusted when necessary using NaCl and tap water according to Gregorio *et al.* (1997). Air temperature inside the greenhouse was in the range of ~25–35 °C and day-time light intensity was in the range of ~600–1000 µmol m^−2^ s^−2^.

For the 3KRG experiments (first batch and second batch), pots were kept in 60-L large trays filled with tap water. Control and salinity treatment trays were placed in alternate rows on the greenhouse floor. Seedlings were thinned to one per pot after 14 d. The level of solution was raised to ~1 cm above the soil surface, and the volume of water in the trays was reconstituted daily with tap water. At the onset of flag leaf appearance (booting stage), leaves from all tillers were trimmed leaving only the top three leaves per tiller. A salinity level of EC 10 dS m^−1^ was imposed for 20 d, after which the plants were returned to non-salinized water until harvest according to Calapit-Palao *et al.* (2013) and Ahmadizadeh *et al.* (2016). In this experiment, only conditions outside the greenhouse were monitored; temperature and relative humidity were in the range of 24–32 °C and 81–92 % for the 3KRG first batch and 20–32 °C and 85–86 % for the 3KRG second batch.

For the fourth experiment, 20 lines from the RDP1 and 20 lines from the 3KRG (10 from the first batch and 10 from the second batch) were selected based on grain yield (top 10 with highest yield and top 10 with lowest yield under salinity) to evaluate the trimming treatment effect. Plants were grown following the protocol for the 3KRG experiments. Four treatments (Control Trimmed, Control Untrimmed, Saline Trimmed and Saline Untrimmed) were imposed. At the onset of flag leaf appearance (booting stage), leaves of the Control and Saline Trimmed treatments were removed, leaving the top three leaves per tiller, whereas all leaves of the Untrimmed plants were kept intact. Temperature inside the greenhouse was in the range of 24–42 °C and relative humidity in the range of ~84–95 %.

### Measurement of leaf Na^+^ and K^+^ concentrationss

Flag leaves were collected 20 d after salinity stress initiation in all experiments and were used in determining Na^+^ and K^+^ concentrations. Dried flag leaf samples (20 mg) were placed in test tubes and extracted with 10 mL of 0.1 N acetic acid in a water bath at 90 °C for 2 h. The extracted tissue was cooled at room temperature, left overnight, and then filtered using Whatman filter paper No. 1. Concentrations of Na^+^ and K^+^ were measured using an atomic absorption spectrophotometer (AAS 3100, Perkin Elmer, USA) at wavelengths of 589 nm for Na^+^ and 766.5 nm for K^+^.

### Yield and yield components

At harvest in all experiments, tiller number per plant, panicle length, main tiller weight, and grain yield per plant were measured, the numbers of filled and unfilled grains were counted, and 100-grain weight from the main tiller was determined. Plant height was measured from the soil surface to the tip of the longest leaf. Shoots were dried in an oven at 50 °C and the weight was determined in all experiments. In order to better compare the level of salinity tolerance across lines with different yield values under control conditions, we used relative grain yield as a standard measure of salinity tolerance, which was calculated as (yield in salinity treatment/yield in control treatment) × 100.

### Assessment of pollen fertility

To determine pollen viability in the fourth experiment, the potassium iodide method was used (Sarhadi *et al.*, 2012). Ten spikelets were collected from individual plants during the heading stage (15 d after flag leaf appearance). Spikelets were fixed with 70 % (v/v) ethanol in vials and stored at 4 °C. The spikelets were dissected on a glass slide to expose the anthers, which were then crushed thoroughly to release the pollen and stained with 1 % (w/v) I_2_-KI solution. The pollen was then examined under a compound microscope at ×4 magnification (Axioplan 2, Zeiss, Germany). Pollen grains stained dark blue were considered viable, and those stained yellow or light-coloured were counted as sterile. Pollen viability was calculated by dividing the number of fertile pollen grains by the total number and presented as a percentage.

### Statistical analysis

Data analysis was performed for each parameter measured based on a split plot design model with three replications using R v. 4.1.2 (R Core Team, 2020). The package *agricolae* was used for analysis of variance (ANOVA), Fisher’s least significant difference (LSD) test (Steel *et al.*, 1997) and correlation (Lin, 1989). The LSD test was performed at the 0.05 significance level to determine specific pairwise differences between means. The package *corrplot* was used for correlation analysis among parameters.

### Genome-wide association study

The GWAS was conducted using the imputed RICE-RP dataset for the RDP1 (Wang *et al.*, 2018) and the 3000 Rice Genome 1 Million Genome-wide association study single-nucleotide polymorphism (SNP) data for the 3KRG (Mansueto *et al.*, 2017). The SNP data were filtered using PLINK 1.90b3b (Chang *et al.*, 2015) based on minor allele frequency (> 0.05), and the linkage disequilibrium (LD) pruned data set was generated by the ‘indep‐pairwise’ command with window size 2 kb and *r*^2^ = 0.85. The ped and map files generated by PLINK were then converted to hapmap files through Tassel 5.0 (Bradbury *et al.*, 2007). Finally, associations for each panel (RDP1, 3KRG first batch and second batch) were run separately and analysed using a linear mixed-model implemented in efficient mixed-model association (EMMA) by the R package of the Genome Association and Prediction Integrated Tool Package (GAPIT) (Zhang *et al.*, 2010; Lipka *et al.*, 2012). The kinship matrix was computed within GAPIT with default settings and the top *K* principal components were used as covariates, where *K* is the number of principal components (PCs) used computed by GAPIT based on the Bayesian information criterion.

Colocation analysis was done by combining the GWAS results from the three experiments (RDP1 and 3KRG) in R v. 4.1.2 using the packages *readr* v. 2.1.2 (https://cran.r-project.org/package=readr), *ggplot2* v. 3.3.5 (Wickham, 2016), *dplyr* v. 1.0.8 (https://cran.r-project.org/package=dplyr) and *reshape2* v. 1.4.4 (Wickham, 2007). Different thresholds of 3, 3.5 and 4 and different window sizes of 1, 2, 5, 20 and 100 kb were compared for detecting colocation To assess the statistical significance of the observed number of colocations, we first computed, for each threshold value, each trait and each window size, the empirical probability of a significant GWAS peak by dividing the number of significant windows for a trait by the total number of windows with SNPs. Next, for each pair of traits the probability of colocation *P*_coloc_ was computed by multiplying the empirical probabilities of the two traits. The *P* value for the observed number of colocations was then computed using the probability of colocation and the observed number of colocations. The number of colocations between relative grain yield and another trait was modelled as a binomial random variable with the parameters expNcoloc = Nwind * p_coloc; sigma = Nwind * p_coloc * (1- p_coloc))^0.5; z = (obsNcoloc—expNcoloc)/sigma, where Nwind is the number of windows, p_coloc is the probability of colocation and obsNcoloc is the number of colocations. If the *P* value of the observed number of colocations was <0.05, it was considered as significant.

In this study, a threshold of −log*P* = 3 and window size of 100 was selected since it identified the most traits with significant numbers of colocations with relative grain yield. While using a less stringent cutoff, e.g. −log*P* = 3 for GWAS may increase the risk of false positives, requiring a peak in one trait to coincide with a peak in another trait helps mitigate this issue. This is because chance associations are unlikely to occur in both traits simultaneously, especially when the traits are independent and measured from distinct agronomic and physiological parameters with relative grain yield, as in this study. Many of the traits related to reproductive stage salinity tolerance are thought to be polygenic, meaning they are controlled by numerous quantitative trait loci (QTLs) with low heritability. This low heritability results from complex genetic regulation and interaction with environmental cues, rather than being influenced by single, major-effect loci. Regional Manhattan plots from all colocating traits for the selected region were generated in R v. 4.1.2 using the packages *ggplot2* v. 3.3.5 (Wickham, 2016), *ggrepel* v. 0.9.4 (https://cran.r-project.org/package=ggrepel), *readr* v. 2.1.2 (https://cran.r-project.org/package=readr), *deplyr* v. 1.0.8 (https://cran.r-project.org/package=dplyr), *qqman* v. 0.1.9 (https://cran.r-project.org/package=qqman) and *vroom* v. 1.6.4 (https://cran.r-project.org/package=vroom). Candidate genes for all loci with physiological traits colocating with relative grain yield were identified using Jbrowse of the Rice SNP-seek database (https://snp-seek.irri.org/_jbrowse.zul?tracks=DNA,msu7gff,msu7snpsv2,msu7indelsv2) and the gene ontology and gene functions using the Rice Genome Annotation Project (RGAP) (http://rice.uga.edu/analyses_search_locus.shtml).

Of the 269 loci that colocated with relative grain yield and other traits associated with reproductive stage salinity tolerance, we selected six loci that showed colocations between relative grain yield and at least seven other traits. From within the 100-kb window that we selected from the colocation analysis, we compared the 20-, 40- and 60-kb subregions. The haplotype groups of the loci in each subregion inside the 100-kb window were generated using the SNP‐seek database ‘Genotype’ function and the ‘Haploview’ function was set to 15 variety groups. Variety order and image tabs were downloaded. The ‘variety order’ file from the SNP‐seek table was then loaded in R to get the kgroup (haplotype group) of each locus. Haplotype groupings were then visualized by the ‘Haplotype view’ tool in SNP‐seek. Boxplots of the haplotype groupings were then generated using the packages *ggplot2* v. 3.3.5 (Wickham, 2016), *ggpubr* v. 0.4.0 (https://github.com/kassambara/ggpubr), *Rmisc* v. 1.5 (https://cran.r-project.org/package=Rmisc), *plyr* v. 1.8.6 (https://cran.r-project.org/package=plyr) and *tibble* (https://cran.r-project.org/package=tibble) in R v. 4.1.2. A 40-kb window subregion was selected in this study since this window resulted in the best phenotypic distinction among haplotypes. Co-expression network analysis was then performed using RicePilaf (Shrestha *et al.*, 2024) to identify sets of genes with similar expression patterns across different samples. GWAS intervals of the three candidate loci were inputted. RiceNet v. 2 (Lee *et al.*, 2015) of the co-expression network and the ClusterONE module detection algorithm (Nepusz *et al.*, 2012) with the default value of the clustering parameter = (2, minimum cluster density 0.30) were selected for the analysis.

## RESULTS

### Genotypic variation in response to salinity stress at reproductive stage

In order to analyse genetic variation in the relationships between rice grain yield and agronomic and physiological traits, panels of 324 rice lines from the RDP1 and two subsets of 197 lines from the 3KRG were evaluated in three greenhouse studies. Since the two diversity panels characterized here were screened with and without leaf trimming, we then compared a subset of contrasting lines in a fourth experiment to investigate whether those protocol differences affected the ranking of lines and if the results could provide insight towards potential tolerance mechanisms.

Significant differences in agronomic and physiological traits, and yield components, were observed between control and saline conditions in the three diversity panel experiments (Supplementary Data Tables S3–S5). Significant differences between treatments with leaves Trimmed and Untrimmed were observed for shoot biomass and leaf Na^+^/K^+^ ratio in the fourth experiment (Supplementary Data Table S6).

Across plant traits, relative grain yield (yield_Saline treatment_/yield_Control treatment_ × 100) was considered as the most important trait to assess the level of salinity tolerance among rice lines. Among the contrasting lines in the fourth experiment, 78 Xuan Wu, YN1353-3, Anadi White and Rinaldo Bersani were consistent with the diversity panel experiments in terms of high relative grain yield under reproductive stage salinity (Table 1). The checks showed low relative grain yield as compared with the test entries (Table 1), which was likely due to photoperiod sensitivity in the case of Pokkali (which did not flower in the 3KRG first-batch experiment) and due to reproductive stage salinity sensitivity in IRRI 154. There was no significant effect of leaf trimming on genotypic rankings for reproductive stage salinity tolerance as indicated by consistent linear correlations (Supplementary Data Fig. S1A–M). We accessed the available data on seedling stage salinity response of the 3KRG (Mansueto *et al.*, 2017), and only one accession (Kogoni 91-1, which was reported to be tolerant to salinity at the seedling stage) also had higher relative grain yield at the reproductive stage in the current study.

**Table 1.** Top ten lines from each treatment/experiment based on relative grain yield under reproductive stage salinity compared with control treatment. Check varieties Pokkali (salinity tolerant) and IRRI 154 (salinity sensitive) are shown at the bottom of the table.

RDP1		3KRG first batch		3KRG second batch		Contrasting lines	
Genotype	Mean relative grain yield (%)	Genotype	Mean relative grain yield (%)	Genotype	Mean relative grain yield (%)	Genotype	Mean relative grain yield (%)
Rathuwee	46.2	Hong Du Bai	85.6	ARC 13544	88.8	78 Xuan Wu	75.8
Rinaldo Bersani	42.1	Kogoni 91-1	82.8	Merle	79.4	Rojofotsy 738	74.7
Dosel	40.1	B 3913 B 16-20 St 28	82.6	IR 87707-445-B-B-B	67.0	YN 1353-3	59.0
Dawebyan	38.4	YN 1353-3	82.2	A 201	65.8	CA 902/B/2/1	55.6
Niquen	34.8	IR 77298-14-1-2-10	80.8	Bai Ri Xian	64.9	L-202	52.2
Dhala Shaitta	31.8	Co 36	78.4	IR64	64.0	Chitraj 14-134	51.5
Pirinae 69	29.8	Rpw 9-4 (Ss 1)	78.4	Anadi White	61.0	Chuan 4	49.1
Norin 20	29.5	Ariana	74.7	AdaySel	60.4	Anadi White	48.7
JM70	28.3	Was 198-B-3-1-3	74.6	Orione	58.9	Kachilon	48.7
Chodongji	27.0	J 6 Ir 520 (Wc 693)	73.2	78 Xuan Wu	58.4	Rinaldo Bersani	47.1
Pokkali	1.10				25.2		12.8
IRRI 154			23.7		24.1		13.7

### Phenotypic correlations under salinity stress at reproductive stage

**Fig. 1.**
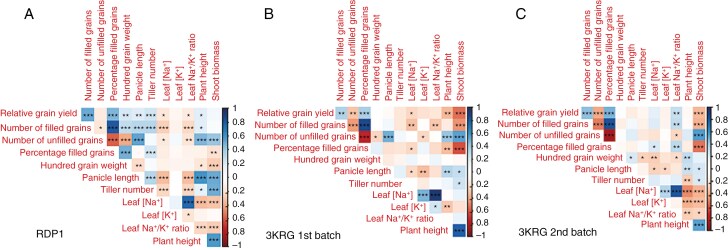
Correlation plots among relative grain yield (Saline/Control multiplied by 100) and its components and agronomic and physiological traits in the (A) RDP1, (B) 3KRG first-batch and (C) 3KRG second-batch experiments under reproductive stage salinity (10 dS m^−1^). *P < 0.05, **P < 0.01, ***P < 0.001.

**Fig. 2.**
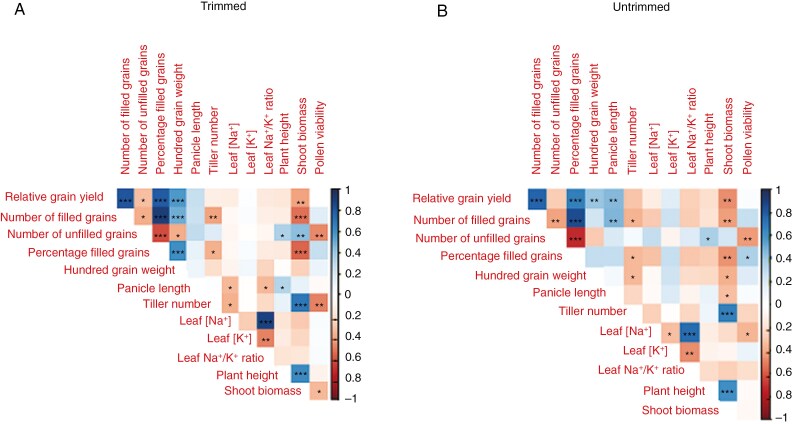
Correlation plots among relative grain yield and its components and agronomic and physiological traits in the fourth experiment on contrasting lines under reproductive stage salinity (10 dS m^−1^) in the (A) Trimmed and (B) Untrimmed treatments. *P < 0.05, **P < 0.01, ***P < 0.001.

**Fig. 3.**
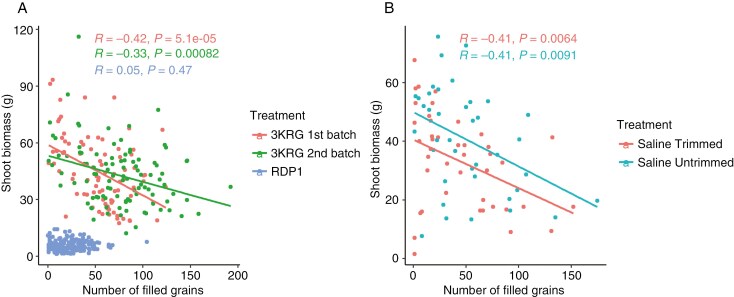
Scatterplots of number of filled grains and shoot biomass of (A) the three diversity panel experiments and (B) the experiment on selected contrasting lines.

In the salinity treatments on the three diversity panel experiments and in the selected contrasting lines, relative grain yield positively correlated with the number of filled grains and percentage of filled grains; the number of filled grains positively correlated with the percentage of filled grains and negatively correlated with the number of unfilled grains; and the number of unfilled grains negatively correlated with the percentage of filled grains and positively correlated with shoot biomass (Fig. 1A–C). No correlation was observed between relative grain yield and pollen viability, which was only measured in the fourth experiment on selected contrasting lines (Fig. 2). A number of plant size-related traits were positively inter-correlated across the three diversity panel experiments, including panicle length, plant height, shoot biomass and tiller number. No correlation was observed between shoot biomass and the number of filled grains in the RDP1 experiment during which the plants were salinized until maturity. However, we observed consistent negative correlations between shoot biomass and the number of filled grains in both 3KRG experiments under reproductive stage salinity (Fig. 3A), including in both the Trimmed and Untrimmed treatments of the selected contrasting lines (Fig. 3B). In general, all of the significant relationships among agronomic and plant size-related traits were consistent for both Trimmed and Untrimmed treatments.

**Fig. 4.**
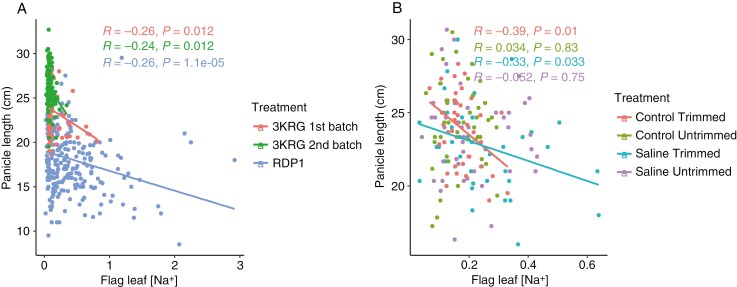
Scatterplots of flag leaf sodium concentration and panicle length from the main tiller of (A) the three diversity panel experiments and (B) among treatments in the fourth experiment comparing selected contrasting lines.

In terms of ion uptake, flag leaf [Na^+^] positively correlated with flag leaf [K^+^] and flag leaf Na^+^/K^+^ ratio (Fig. 1A, B). There was a consistent negative relationship between panicle length and flag leaf [Na^+^] in the three diversity panel experiments (Figs 1 and 4A). Interestingly, among the selected contrasting lines grown in the fourth experiment, this relationship between panicle length and flag leaf [Na^+^] was only significant in the Trimmed treatment and not in the Untrimmed treatment (Fig. 4B).

No correlation was observed between the previously reported standard evaluation scores (SESs; IRRI, 2014) under seedling stage salinity and the relative grain yield under reproductive stage salinity of corresponding lines from the 3KRG experiments (Supplementary Data Fig. S2), as well as in the Trimmed and Untrimmed treatments of the selected contrasting lines in the current study. This is in agreement with the conclusion that different QTLs/genes independently control salinity tolerance at the two different growth stages (Singh *et al.*, 2021).

### Genome-wide association mapping of salinity tolerance-related traits at the reproductive stage

In order to identify candidate genes associated with physiological traits that may contribute to salt tolerance at the reproductive stage and to identify favourable haplotypes to guide breeding of salt-tolerant lines, we conducted GWAS on the data from the three diversity panel experiments. A total of 902 719 SNPs for the RDP1 panel, 468 420 SNPs for the 3KRG first batch and 483 799 SNPs for the 3KRG second batch remained after SNP filtering. Principal component analysis and relatedness heat maps showed genetic clusters that are typical for rice diversity panels (Supplementary Data Figs S3A and B, S5A and B and S7A and B). The Manhattan and quantile–quantile (QQ) plots were generated for the markers and the traits associated with salt tolerance at the reproductive stage (Supplementary Data Figs S4, S6 and S8). Based on Bonferroni correction with the number of independent tests computed by the method of Li and Ji (2005), two genome-wide significant peaks were found in the RDP1 experiment (Table 2; Supplementary Data Fig. S4). Other clear peaks were also observed with a reduced threshold of −log10(*P*) > 5 (Supplementary Data Fig. S4).

**Table 2.** Significant SNPs for salinity tolerance at reproductive stage using the RDP1 panel by GWAS, based on a cutoff of −log10(0.1/total SNPs).

Experiment	Traits	Chromosome	Position	Log*P*
RDP1	Relative panicle length	8	5 363 946	7.27
		8	5 360 623	7.09
		8	5 266 568	7.06
	Relative tiller number	8	20 612 514	6.80
		8	20 616 317	6.45
		8	20 610 100	6.01

**Fig. 5.**
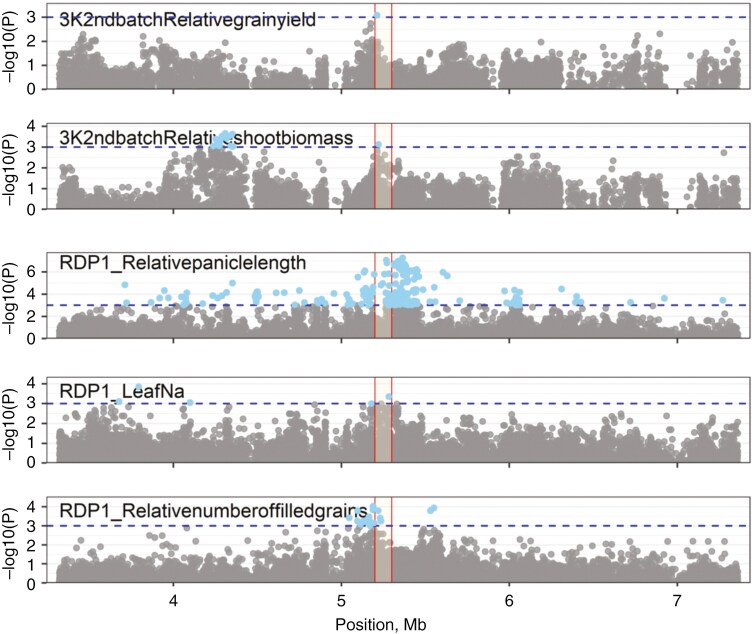
Regional Manhattan plots showing selected colocation regions along with 2-Mb flanking regions Chr8_5.2–5.3 Mb. The colocation regions are highlighted by vertical red lines. The variants passing the significance threshold used for colocation analysis are highlighted in blue.

**Fig. 6.**
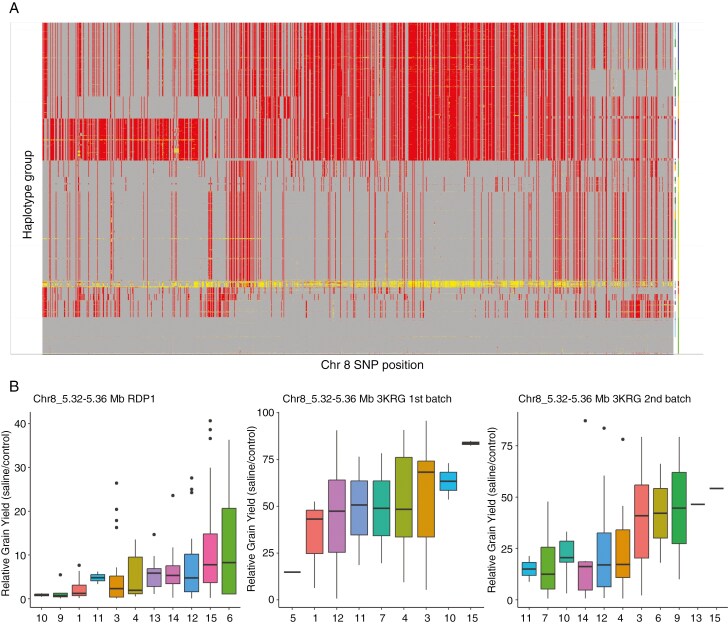
(A) Haploview of Chr8_5.32–5.36 Mb (red, homozygous variant; yellow, heterozygous variant; grey, homozygous variant) and (B) boxplots of the haplotype groupings for the 40-kb subregion inside the 100-kb window on chromosome 8 (the position indicated after the underscore denotes the start of the locus in megabases that showed a high number of colocating GWAS peaks for relative grain yield).

We examined SNP colocations within a 100-kb window between relative grain yield and relative yield components, agronomic traits and physiological traits under salinity from all experiments using a reduced threshold of −log*P* = 3 in order to increase the power of the analysis while still controlling the number of false positives (Table 3). For each trait colocating with relative grain yield, the number of colocations was significantly greater than that predicted to occur by chance (Table 3). Six loci showed the highest number of colocations among relative grain yield, panicle length, tiller number, shoot biomass, number of filled grains and other traits associated with reproductive stage salinity tolerance (Table 4), three of which showed the greatest degree of variation in relative grain yield among haplotype groups and were selected for further investigation. Regional Manhattan plots indicated visibly identifiable peaks at the three loci (Fig. 5; Supplementary Data Figs S9 and S10). Notably, the two significant peaks observed in the RDP1 experiment (Table 2) are at the same loci on chromosome 8 that have been highlighted by the trait/relative grain yield colocations and favourable haplotype analyses (Table 4). Eighty-seven candidate genes from the three colocating relative grain yield GWAS peaks were identified with multiple salinity response traits (Table 5; Supplementary Data Table S7). Candidate genes categorized as expressed proteins, hypothetical proteins, transposon proteins and putative, unclassified, expressed and retrotransposon proteins were discarded from the list of candidate genes. The positions 25.22–25.26 Mb on chromosome 1, 5.32–5.36 Mb on chromosome 8 and 7–7.04 Mb on chromosome 11 from the 40-kb subregion inside the 100-kb window showed the highest relative grain yield among haplotype groups in both the RDP1 and 3KRG experiments (Fig. 6; Supplementary Data Figs S10 and S11). Based on their high values for relative grain yield (Fig. 6B; Supplementary Data Figs S10B and S11B), haplotype group 15 for locus Chr1_25.22–25.26 Mb, haplotype group 15 for locus Chr8_5.32–5.36 Mb and haplotype group 2 for locus Chr11_7–7.04 Mb appeared to be the most favourable.

**Table 3.** Number of observed colocations, expected colocations and *P*-value colocating with relative grain yield from GWAS on 100-grain weight, number of filled grains, number of unfilled grains, panicle length, plant height, shoot biomass and leaf [K^+^] based on a cutoff of −log*P* = 3 and a window of 100 kb.

Experiment	Traits	Observed number of colocations	Expected number of colocations	*P*-value
RDP1	100-Grain weight	25	10.169	1.06E−06
	Number of filled grains	21	14.632	4.77E−02
	Panicle length	38	27.516	2.24E−02
	Shoot biomass	16	9.663	2.06E−02
3KRG first batch	Number of filled grains	9	0.848	4.28E−19
3KRG second batch	Number of filled grains	7	1.102	9.52E−09
	Number of unfilled grains	3	0.715	3.43E−03
	Panicle length	7	2.203	6.14E−04
	Plant height	4	1.786	2.28E−05
	Shoot biomass	14	4.943	5.49E−03
	Leaf [K^+^]	7	2.769	4.88E−02

**Table 4.** Loci with the highest number of colocations among the traits measured in the RDP1, 3KRG first-batch and 3K3G second-batch experiments based on a cutoff of −log*P* = 3 and a window size of 100 kb.

Locus	Number of colocations among the traits measured
Chr1_25.1–25.2 Mb	7
Chr8_ 5.2–5.3 Mb	7
Chr11_6.9–7 Mb	8
Chr11_7–7.1 Mb	8
Chr11_17.9–18.0 Mb	8
Chr11_28.7–28.8 Mb	8

**Table 5.** Candidate genes from the 40-kb subregion within the 100-kb window in which relative grain yield GWAS peaks colocated with multiple salinity response traits.

Chromosome	Start	End	Locus ID	Gene description	Reference
8	5 208 385	5 209 569	LOC_Os08g08960	Cupin domain containing protein, expressed	
	5 222 214	5 223 311	LOC_Os08g08970	Cupin domain containing protein, expressed	
	5 228 822	5 230 034	LOC_Os08g08980	Cupin domain containing protein, expressed	
	5 233 768	5 234 798	LOC_Os08g08990	Cupin domain containing protein, expressed	
	5 238 999	5 240 148	LOC_Os08g09000	Cupin domain containing protein, expressed	
	5 242 495	5 243 657	LOC_Os08g09010	Cupin domain containing protein, expressed	
	5 248 666	5 249 832	LOC_Os08g09020	Cupin domain containing protein, expressed	
	5 254 286	5 255 230	LOC_Os08g09040	Cupin domain containing protein, expressed	
	5 260 152	5 261 299	LOC_Os08g09060	Cupin domain containing protein, expressed	
	5 264 245	5 265 389	LOC_Os08g09080	Cupin domain containing protein, expressed	
	5 273 052	5 268 917	LOC_Os08g09100	RNA recognition motif containing protein, putative, expressed	
	5 279 294	5 274 864	LOC_Os08g09110	NB-ARC domain containing protein, expressed	
	5 320 873	5 318 646	LOC_Os08g09180	Postsynaptic protein CRIPT, putative, expressed	
	5 322 709	5 325 262	LOC_Os08g09190	Auxin efflux carrier component, putative, expressed (OsPILs2)	Ta *et al.*, 2018
	5 334 951	5 327 531	LOC_Os08g09200	Aconitate hydratase protein, putative, expressed	Lekklar *et al.*, 2019
	5 341 430	5 345 421	LOC_Os08g09210	Phosphoribosylamine–glycine ligase, putative, expressed	
	5 347 221	5 345 885	LOC_Os08g09220	OsFBX262 – F-box domain containing protein, expressed	
	5 352 105	5 363 367	LOC_Os08g09230	Starch synthase III, putative, expressed	
	5 364 349	5 366 503	LOC_Os08g09240	Autophagy-related protein, putative, expressed	
	5 377 152	5 373 216	LOC_Os08g09250	Glyoxalase family protein, putative, expressed	
	5 384 023	5 379 310	LOC_Os08g09260	tRNA synthetase, putative, expressed	Lekklar *et al.*, 2019
	5 384 443	5 388 851	LOC_Os08g09270	Pentatricopeptide, putative, expressed	

**Fig. 7.**
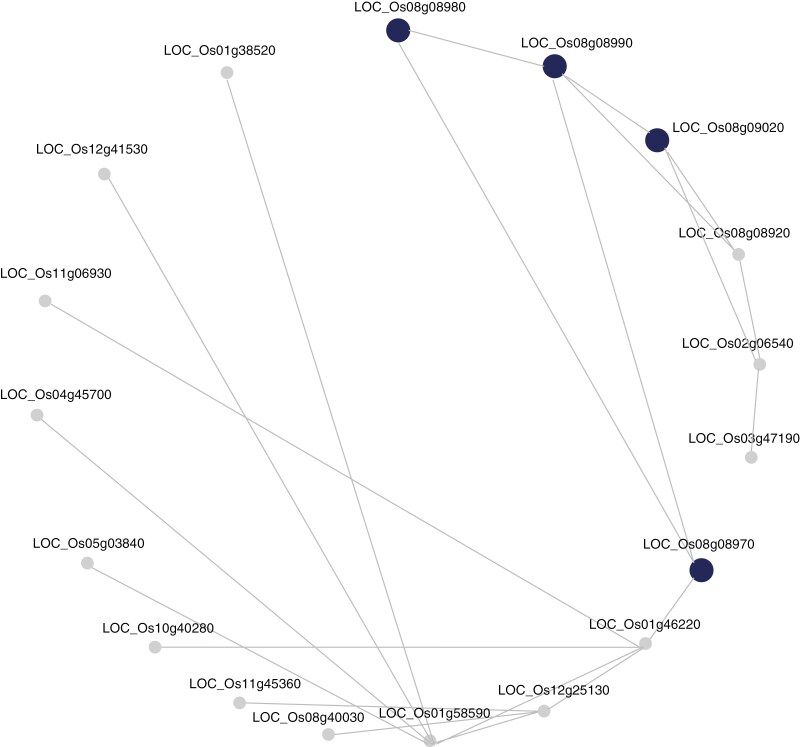
Co-expression network analysis of the Chr8_5.32–5.36 Mb GWAS interval using RiceNet v. 2 in RicePilaf, showing a significantly enriched ClusterONE module 576 (adjusted *P*-value = 5.6E−08). The larger, dark blue nodes refer to the genes implicated by the GWAS interval and the lines connecting them represent relationships between genes. The more connected the genes are, the more important the gene in the interaction network.

We then performed a co-expression network analysis for the three candidate loci using the RicePilaf tool (Shrestha *et al.*, 2024). The set of genes in the interval Chr8_5.32–5.36 Mb was significantly enriched in 12 co-expression modules annotated with gene ontology terms and pathways. The corresponding numbers of modules annotated for the other two loci were 11 (Chr1_25.22–25.26 Mb) and 2 (Chr11_7.0–7.04 Mb). ‘Apoplast’ was the most frequently observed gene ontology among the modules associated with the co-expression network identified on Chr8_5.32–5.36 Mb (Table 5; Fig. 7) and Chr1_25.22–25.26 Mb (Supplementary Data Table S8; Supplementary Data Fig. S12). The other enriched gene ontology terms included cell death (Table 6), cutin biosynthetic process and lignin catabolic process (Supplementary Data Table S8).

**Table 6.** The most frequently listed gene ontology terms enriched in different co-expression modules for the multi-trait GWAS interval on chromosome 8.

Gene ontology term	Module	Genes
Apoplast	576	LOC_Os08g08970; LOC_Os08g08980; LOC_Os08g08990;
		LOC_Os08g08920; LOC_Os08g09020
	755	LOC_Os08g09060; LOC_Os08g09080; LOC_Os08g09010;
		LOC_Os08g09040; LOC_Os08g08970; LOC_Os08g08980;
		LOC_Os08g08990; LOC_Os08g08920; LOC_Os08g09020;
		LOC_Os08g09000
	1538	LOC_Os08g08920; LOC_Os08g09020
Cell death	857	LOC_Os02g10310
	1295	LOC_Os04g36680
	1930	LOC_Os02g10310
	2240	LOC_Os04g36680

## DISCUSSION

This study compared two different rice diversity panels to explore the extent of genetic variation in salinity tolerance at the reproductive stage. We aimed to identify the key salinity tolerance mechanisms based on phenotypic correlations and candidate gene function, and identify favourable haplotypes that may be used to develop improved rice lines with salinity tolerance at the reproductive stage.

With the treatments imposed in this study, we were able to evaluate the relative mechanistic effects of exclusion (i.e. salt being prevented from reaching the leaf tissue or panicle) and compartmentation (i.e. salt accumulation in the leaf tissue and not the panicle). Plant species classified as sodium excluders regulate ion transport and maintain low levels of salt in their tissues (Chen *et al.*, 2018). Across the three diversity panel experiments in this study, we found a negative relationship between flag leaf [Na^+^] and panicle length. This observation and the positive correlation between biomass and unfilled grains in the RDP1 experiment, as well as the lack of effect of trimming the leaves on the genotype rankings in the fourth experiment (Supplementary Data Fig. S1), indicates that salt exclusion was the key tolerance mechanism in this study. It is known that salinity stress during the reproductive stage can cause a significant decrease in panicle length (Gerona *et al.*, 2019; Zhang *et al.*, 2022). Increased salt exclusion functions to reduce the rate at which salt accumulates in the roots and transpiring organs (Rahman *et al.*, 2016), which may result in reduced restriction of panicle length. Interestingly, in the fourth experiment the relationship between flag leaf [Na^+^] and panicle length was observed only in the Trimmed treatments and not in the Untrimmed treatments. One possible reason for this is that since only the top three leaves (including the flag leaf) were left intact in the Trimmed treatments, the source left for the carbohydrate assimilation was very limited, resulting in significantly reduced panicle lengths in the Control Trimmed treatment as well as in the Salinity Trimmed treatment. While other factors, such as pollen fertility, Na^+^ concentration and Na^+^/K^+^ ratio in the flag leaf, were also found to contribute to salinity tolerance at the reproductive stage in rice (as reviewed by Singh *et al.*, 2021), the relationship between flag leaf [Na^+^] and panicle length was the most consistent salt tolerance mechanism observed here.

If compartmentation of Na^+^ in the leaves had been an important reproductive stage salinity tolerance mechanism in this study, we expected to see larger genotypic differences between the Trimmed and Untrimmed treatments. The older leaves would have acted as a sink in the Untrimmed biomass to absorb salt and keep it from reaching the panicle. Although this was unlikely based on the three correlations mentioned above, we cannot rule out partitioning of Na^+^ to older leaves because that was not measured in this study. However, larger biomass was in general not advantageous under reproductive stage salinity in this study. The negative correlation between shoot biomass and number of filled grains (Fig. 3) suggests that there was some detrimental aspect of having high biomass under reproductive stage salinity. It may be that the greater transpiration demand of high-biomass lines increases salt uptake. In support of this finding, Yamazaki *et al.* (2020) reported a negative correlation between biomass and salt tolerance in sorghum. Further research is needed to understand the underlying mechanisms and develop strategies to mitigate this negative impact of biomass, which is an important trait for high yield potential under favourable conditions. Laza *et al.* (2003) also reported that the relationship between grain yield and biomass production was relatively weak, and that harvest index is more important than biomass in determining grain yield under suboptimum growing conditions. The growing conditions were another factor to consider in the relationship between biomass and salt uptake since a higher vapor pressure deficit (VPD) would be expected to increase salt uptake due to increased transpiration, although the VPD of the conditions in our experiments was generally low.

Given that sodium exclusion was identified as the key tolerance mechanism based on physiological measurements, favourable haplotypes and candidate loci associated with salinity tolerance functions were identified based on colocations from GWAS at reproductive stage. In future studies, we recommended not to separate batches of lines, which limited the number of lines in each GWAS. A better alternative would be to plant more lines per experiment but to grow each replication at different times to maintain similar sizes and labour requirements.

From the three loci (on chromosomes 1, 8 and 11) identified for having the greatest numbers of colocations in the multi-trait GWAS, we identified 87 candidate genes associated with salinity tolerance for relative grain yield. LOC_Os08g09200, one of the candidate genes detected by GWAS for relative grain yield, encodes an aconitate hydratase protein that functions in metabolism and energy production affecting salinity tolerance (Shahbazi *et al.*, 2023). This gene overlaps with a QTL for chlorophyll levels between marker E37-M61-7 and RM152 in the mapping study of Ghomi *et al.* (2013) for salinity tolerance at the seedling stage using an Iranian rice population. Interestingly, the same gene was also identified by Lekklar *et al.* (2019) in a GWAS using Thai rice lines for salinity tolerance at the flowering stage. The same gene was also identified by Li *et al.* (2015), where it regulated the expression pattern of an essential element in the response of the TCA cycle to abiotic stress and suggested a possible regulatory pathway for the transcriptional regulation of rice respiratory metabolism genes in response to heat.

Another candidate gene from the chromosome 8 colocating region, LOC_Os08g09190, also known as OsPILS2, functions as an auxin efflux carrier component. Auxin regulates the developmental plasticity of the plant root in most abiotic stress conditions, including salinity and water deficit (Korver *et al.*, 2018). It also plays an important role in plant developmental responses and stress, modulating a complex balance of biosynthesis, transport and signalling that improves physiological changes in plant architecture and Na^+^ accumulation (Ribba *et al.*, 2020). This gene overlaps with the QTLs for panicle branch number and panicle branch length identified in the study of Ta *et al.* (2018) in the GWAS of a Vietnamese landrace panel of rice controlling panicle morphological traits.

LOC_Os11g12740, another candidate gene on chromosome 11, encodes a peptide transporter, PTR2, but has no prior information yet related to salt tolerance mechanisms in rice (Syarifudin and Chadchawan, 2022). This gene was also identified by Li *et al.* (2009) in their study on the architecture of rice inflorescence, wherein SP1 (short panicle1) was found to regulate spikelet formation in rice. Interestingly, two of the candidate loci identified in this study were found on chromosomes 1 (25.22–25.26 Mb) and 11 (6.9–7.19, 17.8–18.1 and 28.7–28.8 Mb) for which no salinity tolerance QTL was previously reported.

To further add confidence to the key salt tolerance mechanisms observed from the physiological measurements and candidate gene functions, we explored co-expression networks of the candidate genes to reveal key reproductive stage salinity tolerance mechanisms based on gene ontologies. ‘Apoplast’, being the most frequent gene ontology across co-expression modules from two of the loci (Chr8_5.32–5.36 Mb and Chr1_25.22–25.26 Mb) identified from the multi-trait GWAS, plays a crucial role in salt exclusion by creating barriers that prevent the movement of Na^+^ in the plant. Maintaining low Na^+^ levels in the cytoplasm is crucial for plants to survive in saline conditions (Krishnamurthy *et al.*, 2009). High uptake of Na^+^ levels in the apoplast are associated with reduced plant survival during salt stress. In rice, the movement of water and solutes through the apoplast plays a significant role in determining salt tolerance. Strengthening apoplastic barriers reduces sodium accumulation in shoots, enhancing plant resilience under saline conditions (Krishnamurthy *et al.*, 2011). Therefore the gene ontologies most frequently associated with the co-expression networks of the candidate genes identified here appear to support our conclusion from the physiological measurements that salt exclusion was the key tolerance mechanism at the reproductive stage in rice.

## Conclusions

This study investigated the effects of salinity stress on rice at the reproductive stage and found that salt exclusion was the primary mechanism for tolerance, as evidenced by reduced panicle length with increased [Na^+^] in the flag leaf, a lack of effect of trimming leaves on genotypic rankings, and the functions associated with candidate genes and their co-expression networks. Three colocating loci from the multi-trait GWAS exhibited favourable haplotypes based on their high relative grain yield. Following validation and subsequent confirmation of their low frequency in the current breeding pool, these loci may be deployed in breeding pipelines by introgressing them into elite lines, either to improve existing varieties or to be used in further crossing.

## SUPPLEMENTARY DATA

Supplementary data are available at *Annals of Botany* online and consist of the following. Table S1: list of 324 RDP1 lines used in this study. Table S2: list of a subset of 197 lines from the 3KRG panel used in this study. Table S3: ANOVA for different agronomic and physiological traits in RDP1 at reproductive stage under salt stress of 10 d Sm^−1^. Table S4: ANOVA for different agronomic and physiological traits in the 3KRG first batch at reproductive stage under salt stress of 10 dSm^−1^. Table S5: ANOVA for different agronomic and physiological traits in the 3KRG second batch at reproductive stage under salt stress of 10 dSm^−1^. Table S6: ANOVA for different agronomic and physiological traits in the selected contrasting lines at reproductive stage under salt stress of 10 dSm^−1^. Table S7: candidate genes from the 40-kb subregion within the 100-kb window on chromosomes 1 and 11 in which relative grain yield GWAS peaks colocated with multiple salinity response traits. Table S8: the most frequently listed gene ontology terms enriched in different modules for the multi-trait GWAS interval on chromosome 1. Figure S1: scatterplot of (A) relative grain yield, (B) panicle length (C) number of filled grains, (D) number of unfilled grains, (E) percentage of filled grains, (F) 100-grain weight, (G) pollen viability, (H) leaf [Na^+^], (I) leaf [K^+^], (J) leaf Na^+^/K^+^ ratio, (K) plant height, (L) shoot biomass and (M) tiller number of Trimmed and Untrimmed treatments of the selected contrasting lines at the reproductive stage. Figure S2: scatterplot of standard evaluation score (SES) extracted from the SNP-seek database (snp-seek.irri.org) under seedling stage salinity at an EC12 dSm^−1^ and relative grain yield at the reproductive stage of the 3KRG. Figure S3: population structure in the RDP1 panel. Figure S4: Manhattan plot and quantile–quantile (QQ) plots of relative grain yield, relative 100-grain weight, relative number of filled grains, relative number of unfilled grains, relative panicle length, relative plant height, relative shoot biomass, relative tiller number, leaf [Na^+^] under salinity, leaf [K^+^] under salinity and leaf Na^+^/K^+^ ratio under salinity using RDP1. Figure S5: population structure in the 3KRG first batch panel. (A) Heat map of the kinship matrix and (B) scatterplot of the first two principal components (PCs) produced by GAPIT. Figure S6: Manhattan plot and quantile–quantile (QQ) plots of relative grain yield, relative 100-grain weight, relative number of filled grains, relative number of unfilled grains, relative panicle length, relative plant height, relative shoot biomass, relative tiller number, leaf [Na^+^] under salinity, leaf [K^+^] under salinity and leaf Na^+^/K^+^ ratio under salinity using the 3KRG first batch panel. Figure S7: population structure in the 3KRG second-batch panel. Figure S8: Manhattan plot and quantile–quantile (QQ) plots of relative grain yield, relative 100-grain weight, relative number of filled grains, relative number of unfilled grains, relative panicle length, relative plant height, relative shoot biomass, relative tiller number, leaf [Na^+^] under salinity, leaf [K^+^] under salinity and leaf Na^+^/K^+^ ratio under salinity using the 3KRG second-batch panel. Figure S9: regional Manhattan plots showing selected colocation regions along with 2-Mb flanking regions (A) Chr1 25.1–25.2 Mb and (B) Chr11 7.0–7.1 Mb. Figure S10: (A) haploview of Chr1_25.22–25.26 Mb and (B) boxplot of the haplotype groupings for the 40-kb subregion inside the 100-kb window on chromosome 1 (the position indicated after the underscore denotes the start of the locus, in megabases, that showed a high number of colocating GWAS peaks for relative grain yield). Figure S11: (A) haploview of Chr11_7–7.04 Mb and (B) boxplot of the haplotype groupings for the 40-kb subregion inside the 100-kb window on chromosome 11 (the position indicated after the underscore denotes the start of the locus, in megabases, that showed a high number of colocating GWAS peaks for relative grain yield). Figure S12: co-expression network analysis of the Chr1_25.22–25.26 GWAS interval showing enriched ClusterONE module 2580 (adjusted *P*-value = 2.442115E−02) of RiceNet v. 2 in RicePilaf. The larger, dark blue nodes refer to the genes implicated by the GWAS interval and the lines between them represent relationships between genes.

mcae227_suppl_Supplementary_Tables_S1-S8_Figures_S1-S11

## Data Availability

All phenotypic data will be made available on the IRRI Dataverse site upon publication.
