# Supplementary material for: Leaf Na+ effects and multi-trait GWAS point to salt exclusion as the key mechanism for reproductive stage salinity tolerance in rice
Source: Ann Bot. 2024 Dec 28;135(5):949–62. doi: 10.1093/aob/mcae227 (PMC12064422; doi:10.1093/aob/mcae227)
Supplement: mcae227_suppl_Supplementary_Tables_S1-S8_Figures_S1-S11 [file mcae227_suppl_supplementary_tables_s1-s8_figures_s1-s11.docx]

**de Ocampo et al.**

**Leaf Na^+^ effects and multi-trait GWAS point to salt exclusion as the key mechanism for reproductive stage salinity tolerance in rice**

**Supplementary Files**

**Table S1.** List of 324 RDP1 lines used in this study.

| No | IRGC Acc No | Line | Subpopulation | Origin |
| --- | --- | --- | --- | --- |
| 1 | 117600 | Sze Guen Zim | IND | China |
| 2 | 117602 | Bellardone | TEJ | France |
| 3 | 117605 | Basmati 217 | TRJ | India |
| 4 | 117606 | Coppocina | TRJ | Bulgaria |
| 5 | 117607 | Carolina Gold | TRJ | United States |
| 6 | 117609 | Cuba 65 | TRJ | Cuba |
| 7 | 117610 | Early Wataribune | TEJ | Japan |
| 8 | 117611 | Geumobyeo | TEJ | South Korea |
| 9 | 117614 | Pai Hok Glutinous | IND | Hong Kong |
| 10 | 117615 | Iguape Cateto | TRJ | Haiti |
| 11 | 117616 | Bahia | TEJ | Spain |
| 12 | 117618 | Khao Gaew | AUS | Thailand |
| 13 | 117619 | Luk Takhar | TEJ | Afghanistan |
| 14 | 117620 | Ming Hui | IND | China |
| 15 | 117621 | MTU9 | IND | India |
| 16 | 117622 | RTS4 | IND | Vietnam |
| 17 | 117626 | WC 4443 | TRJ | Bolivia |
| 18 | 117628 | Shim Balte | AUS | Iraq |
| 19 | 117632 | 68-2 | TEJ | France |
| 20 | 117632 | 68-2 | TEJ | France |
| 21 | 117633 | ARC 6578 | AUS | India |
| 22 | 117635 | Tia Bura | TRJ | Indonesia |
| 23 | 117638 | SML 242 | IND | Suriname |
| 24 | 117638 | Toploea 70/76 | TEJ | Romania |
| 25 | 117639 | Sab Ini | TEJ | Egypt |
| 26 | 117641 | ARC 7229 | AUS | India |
| 27 | 117643 | Baber | TEJ | India |
| 28 | 117645 | Sadri Belyi | AROMATIC | Azerbaijan |
| 29 | 117646 | Vary Vato 462 | ADMIX | Madagascar |
| 30 | 117647 | Baghlani Nangarhar | TEJ | Afghanistan |
| 31 | 117651 | Vialone | TEJ | Italy |
| 32 | 117658 | Binulawan | IND | Philippines |
| 33 | 117659 | BJ 1 | AUS | India |
| 34 | 117660 | IRAT 44 | TRJ | Burkina Faso |
| 35 | 117661 | Black Gora | AUS | India |
| 36 | 117662 | Blue Rose | ADMIX | Louisiana |
| 37 | 117663 | Byakkoku Y 5006 Seln | IND | Australia |
| 38 | 117664 | Bombon | TEJ | Spain |
| 39 | 117666 | British Honduras Creole | TRJ | Belize |
| 40 | 117668 | Bul Zo | TEJ | South Korea |
| 41 | 117669 | C57-5043 | TRJ | United States |
| 42 | 117670 | H256-76-1-1-1 | TRJ | Argentina |
| 43 | 117671 | Caawa/Fortuna 6-103-15 | TRJ | Taiwan |
| 44 | 117672 | Kon Suito | TEJ | Mongolia |
| 45 | 117673 | Niquen | TRJ | Chile |
| 46 | 117674 | Canella De Ferro | TRJ | Brazil |
| 47 | 117675 | Carolina Gold | TRJ | United States |
| 48 | 117676 | Carolina Gold Sel | TRJ | United States |
| 49 | 117677 | Pirinae 69 | ADMIX | Former Yugoslavia |
| 50 | 117679 | Chau | IND | Vietnam |
| 51 | 117682 | Chiem Chanh | IND | Vietnam |
| 52 | 117683 | IR-44595 | IND | Nepal |
| 53 | 117684 | Chinese | TEJ | China |
| 54 | 117686 | Chodongji | TEJ | South Korea |
| 55 | 117687 | Chuan 4 | AUS | Taiwan |
| 56 | 117691 | CS-M3 | TEJ | United States-CA |
| 57 | 117694 | Criollo La Fria | IND | Venezuela |
| 58 | 117696 | Delrex | TRJ | United States |
| 59 | 117700 | Dam | ADMIX | Thailand |
| 60 | 117701 | Darmali | ADMIX | Nepal |
| 61 | 117702 | Dee Geo Woo Gen | IND | Taiwan |
| 62 | 117705 | Dhala Shaitta | AUS | Bangladesh |
| 63 | 117707 | Dom Zard | AROMATIC | Iran |
| 64 | 117709 | Caucasica | TEJ | Former Soviet Union |
| 65 | 117710 | Dom-sufid | AROMATIC | Iran |
| 66 | 117721 | Dourado Agulha | TRJ | Brazil |
| 67 | 117723 | DV85 | AUS | Bangladesh |
| 68 | 117725 | DZ78 | AUS | Bangladesh |
| 69 | 117728 | 27 | TRJ | Dominican Republic |
| 70 | 117733 | WAB 502-13-4-1 | TRJ | Cote D'Ivoire |
| 71 | 117735 | Fortuna | TRJ | United States |
| 72 | 117738 | C1-6-5-3 | ADMIX | Mexico |
| 73 | 117739 | Ghati Kamma Nangarhar | AUS | Afghanistan |
| 74 | 117740 | Gogo Lempuk | TRJ | Indonesia |
| 75 | 117744 | Guan-Yin-Tsan | IND | China |
| 76 | 117746 | Hon Chim | IND | Hong Kong |
| 77 | 117747 | Maratelli | TEJ | Italy |
| 78 | 117748 | Vavilovi | TEJ | Kazakhstan |
| 79 | 117749 | Hatsunishiki | TEJ | Japan |
| 80 | 117750 | Hsia Chioh Keh Tu | IND | Taiwan |
| 81 | 117751 | Hu Lo Tao | TEJ | China |
| 82 | 117752 | I-Geo-Tze | ADMIX | Taiwan |
| 83 | 117753 | Shangyu 394 | TEJ | China |
| 84 | 117754 | IAC 25 | TRJ | Brazil |
| 85 | 117755 | IR 36 | IND | Philippines |
| 86 | 117756 | Bombilla | TEJ | Spain |
| 87 | 117756 | Zerawchanica Karatalski | TEJ | Poland |
| 88 | 117757 | IR 8 | IND | Philippines |
| 89 | 117758 | IRAT 177 | TRJ | French Guiana |
| 90 | 117759 | Tox 782-20-1 | TRJ | Nigeria |
| 91 | 117760 | JM70 | IND | Mali |
| 92 | 117761 | IRGA 409 | IND | Brazil |
| 93 | 117762 | Riz Local | ADMIX | Burkina Faso |
| 94 | 117763 | Jambu | TRJ | Indonesia |
| 95 | 117764 | Jaya | IND | India |
| 96 | 117766 | JC149 | IND | India |
| 97 | 117768 | Jhona 349 | AUS | India |
| 98 | 117769 | Jouiku 393G | TEJ | Japan |
| 99 | 117770 | Kaukkyi Ani | ADMIX | Myanmar |
| 100 | 117773 | Kamenoo | TEJ | Japan |
| 101 | 117776 | Kasalath | AUS | India |
| 102 | 117779 | Gambiaka Sebela | TEJ | Mali |
| 103 | 117780 | Leah | TRJ | Bulgaria |
| 104 | 117782 | Kiang-Chou-Chiu | IND | Taiwan |
| 105 | 117786 | Kitrana 508 | AROMATIC | Madagascar |
| 106 | 117788 | Saku | ADMIX | Mongolia |
| 107 | 117789 | KU115 | ADMIX | Thailand |
| 108 | 117792 | Kun-Min-Tsieh-Hunan | IND | China |
| 109 | 117793 | L-202 | TRJ | United States_CA |
| 110 | 117794 | LAC 23 | TRJ | Liberia |
| 111 | 117796 | Lacrosse | ADMIX | United States |
| 112 | 117797 | Lemont | TRJ | United States |
| 113 | 117802 | Leung Pratew | IND | Thailand |
| 114 | 117807 | Mansaku | TEJ | Japan |
| 115 | 117808 | Amposta | TEJ | Puerto Rico |
| 116 | 117808 | LD 24 | IND | Sri Lanka |
| 117 | 117811 | Mehr | AUS | Iran |
| 118 | 117812 | Baldo | ADMIX | Italy |
| 119 | 117815 | Moroberekan | TRJ | Guinea |
| 120 | 117272 | Moroberekan | TRJ | Guinea |
| 121 | 117817 | Mudgo | IND | India |
| 122 | 117818 | N 22 | TRJ | India |
| 123 | 117819 | Norin 20 | TEJ | Japan |
| 124 | 117820 | Hunan Early Dwarf No. 3 | IND | China |
| 125 | 117822 | Nova | ADMIX | United States |
| 126 | 117824 | O-Luen-Cheung | IND | Taiwan |
| 127 | 117825 | Azerbaidjanica | TEJ | Azerbaijan |
| 128 | 117826 | Oro | TEJ | Chile |
| 129 | 117828 | Oryzica Llanos 5 | IND | Colombia |
| 130 | 117829 | OS6 | TRJ | Nigeria |
| 131 | 117831 | Ostiglia | TEJ | Argentina |
| 132 | 117832 | M. Blatec | ADMIX | Macedonia |
| 133 | 117833 | Padi Kasalle | TRJ | Indonesia |
| 134 | 117834 | Pate Blanc Mn 1 | TRJ | Cote D'Ivoire |
| 135 | 117835 | Pagaiyahan | IND | Taiwan |
| 136 | 117836 | Sri Malaysia Dua | TEJ | Malaysia |
| 137 | 117840 | Pappaku | IND | Taiwan |
| 138 | 117841 | Pato De Gallinazo | ADMIX | Australia |
| 139 | 117843 | Pratao | TRJ | Brazil |
| 140 | 117844 | Triomphe Du Maroc | TEJ | Morocco |
| 141 | 117846 | Peh-Kuh | IND | Taiwan |
| 142 | 117848 | Peh-Kuh-Tsao-Tu | IND | Taiwan |
| 143 | 117849 | Phudugey | AUS | Bhutan |
| 144 | 117850 | Rathuwee | IND | Sri Lanka |
| 145 | 117852 | Bulgare | TEJ | France |
| 146 | 117854 | Radin Ebos 33 | IND | Malaysia |
| 147 | 117858 | Razza 77 | TEJ | Italy |
| 148 | 117859 | Rikuto Kemochi | TEJ | Japan |
| 149 | 117860 | Rinaldo Bersani | ADMIX | Italy |
| 150 | 117863 | Rojofotsy 738 | ADMIX | Madagascar |
| 151 | 117864 | CA 902/B/2/1 | AUS | Chad |
| 152 | 117865 | Sigadis | IND | Indonesia |
| 153 | 117866 | Agusita | TEJ | Hungary |
| 154 | 117868 | S4542A3-49B-2B12 | TRJ | United States |
| 155 | 117869 | Saturn | ADMIX | United States |
| 156 | 117870 | Saraya | AUS | Fiji |
| 157 | 117872 | Paraiba Chines Nova | IND | Brazil |
| 158 | 117879 | Seratoes Hari | IND | Indonesia |
| 159 | 117880 | Shai-Kuh | IND | China |
| 160 | 117881 | Shinriki | TEJ | Japan |
| 161 | 117883 | Halwa Gose Red | AUS | Iraq |
| 162 | 117884 | Shoemed | TEJ | United States |
| 163 | 117887 | Short Grain | IND | Thailand |
| 164 | 117889 | SLO 17 | IND | India |
| 165 | 117890 | Sintane Diofor | IND | Burkina Faso |
| 166 | 117894 | Tchibanga | IND | Gabon |
| 167 | 117895 | Kaukau | AUS | Mali |
| 168 | 117898 | Osogovka | ADMIX | Macedonia |
| 169 | 117900 | T 1 | AUS | India |
| 170 | 117901 | WC 521 | ADMIX | China |
| 171 | 117902 | T26 | AUS | India |
| 172 | 117903 | Ta Hung Ku | TEJ | China |
| 173 | 117904 | Ta Mao Tsao | TEJ | China |
| 174 | 117905 | Taichung Native 1 | IND | Taiwan |
| 175 | 117907 | Tainan Iku 487 | TEJ | Taiwan |
| 176 | 117908 | Taipei 309 | TEJ | Taiwan |
| 177 | 117911 | Tokyo Shino Mochi | ADMIX | Japan |
| 178 | 117912 | TKM6 | IND | India |
| 179 | 117914 | Sadri Tor Misri | ADMIX | Iran |
| 180 | 117915 | Taducan | IND | Philippines |
| 181 | 117918 | Trembese | TRJ | Indonesia |
| 182 | 117920 | SL 22-613 | ADMIX | Sierra Leone |
| 183 | 117921 | Tsipala 421 | ADMIX | Madagascar |
| 184 | 117922 | Chibica | TEJ | Mozambique |
| 185 | 117923 | Arabi | ADMIX | Egypt |
| 186 | 117924 | B6616A4-22-Bk-5-4 | TRJ | United States |
| 187 | 117927 | Padi Pagalong | TRJ | Malaysia |
| 188 | 117928 | Sundensis | IND | Kazakhstan |
| 189 | 117929 | Hiderisirazu | ADMIX | Japan |
| 190 | 117931 | ECIA76-S89-1 | IND | Cuba |
| 191 | 117932 | WAB 501-11-5-1 | TRJ | Cote D'Ivoire |
| 192 | 117935 | WC 4419 | TRJ | Honduras |
| 193 | 117936 | Yabani Montakhab 7 | TEJ | Egypt |
| 194 | 117939 | YRL-1 | ADMIX | Australia |
| 195 | 117941 | PI 298967-1 | ADMIX | Australia |
| 196 | 117942 | Dosel | TEJ | Spain |
| 197 | 117942 | Italica Carolina | TEJ | Poland |
| 198 | 117943 | Zhenshan 2 | IND | China |
| 199 | 117944 | Nipponbare | TEJ | Japan |
| 200 | 117632 | 583 | TRJ | Ecuador |
| 201 | 117935 | WC 3397 | TRJ | Jamaica |
| 202 | 117819 | N12 | AROMATIC | India |
| 203 | 117636 | Ai-Chiao-Hong | IND | China |
| 204 | 117600 | Aijiaonante | IND | China |
| 205 | 117643 | Asse Y Pung | TRJ | Philippines |
| 206 | 117264 | Azucena | TRJ | Philippines |
| 207 | 117652 | Basmati | AROMATIC | Pakistan |
| 208 | 117658 | Bico Branco | AROMATIC | Brazil |
| 209 | 117664 | Boa Vista | TRJ | El Salvador |
| 210 | 117713 | Djimoron | IND | Guinea |
| 211 | 117735 | Firooz | AROMATIC | Iran |
| 212 | 117750 | Honduras | TRJ | Honduras |
| 213 | 117760 | IRAT 13 | TRJ | Cote D'Ivoire |
| 214 | 117773 | Kalamkati | AUS | India |
| 215 | 117813 | Melanotrix | TEJ | Tajikistan |
| 216 | 117867 | RT 1031-69 | TRJ | Zaire |
| 217 | 117890 | Sinampaga Selection | TRJ | Philippines |
| 218 | 117897 | Sultani | TRJ | Egypt |
| 219 | 117912 | TeQing | IND | China |
| 220 | 117916 | TOg 7178 | ADMIX | Senegal |
| 221 | 117927 | Varyla | ADMIX | Madagascar |
| 222 | 117943 | ZHE 733 | IND | China |
| 223 | 117691 | CO18 | IND | India |
| 224 | 117840 | Pao-Tou-Hung | IND | China |
| 225 | 117622 | RTS14 | IND | Vietnam |
| 226 | 117629 | 318 | TRJ | TURKEY |
| 227 | 117630 | 519 | IND | Uruguay |
| 228 | 117634 | 9524 | AUS | India |
| 229 | 117631 | 56-122-23 | TEJ | Thailand |
| 230 | 117640 | ARC 10086 | ADMIX | India |
| 231 | 117642 | ARC 10376 | AUS | India |
| 232 |  | ASD 1 | TEJ | India |
| 233 | 117644 | Aswina 330 | AUS | Bangladesh |
| 234 | 117656 | Berenj | ADMIX | Afghanistan |
| 235 | 117623 | BR24 | IND | Bangladesh |
| 236 | 117678 | Cenit | TRJ | Argentina |
| 237 | 117681 | Chang Ch'Sang Hsu Tao | IND | China |
| 238 | 117685 | China 1039 | IND | China |
| 239 |  | CI 11011 | ADMIX | United States |
| 240 | 117690 | CI 11026 | ADMIX | United States |
| 241 | 117692 | Coarse | AUS | Pakistan |
| 242 | 117693 | Cocodrie | TRJ | United States |
| 243 | 117697 | CTG 1516 | AUS | Bangladesh |
| 244 | 117699 | Cybonnet | TRJ | United States |
| 245 | 117282 | Cypress | TRJ | United States |
| 246 | 117703 | Dawebyan | IND | Myanmar |
| 247 | 117704 | DD 62 | AUS | Bangladesh |
| 248 | 117708 | Deokjeokjodo | TEJ | Korea |
| 249 | 117711 | DJ 123 | AUS | Bangladesh |
| 250 | 117712 | DJ 24 | AUS | Bangladesh |
| 251 | 117714 | DK 12 | AUS | Bangladesh |
| 252 | 117715 | DM 43 | AUS | Bangladesh |
| 253 | 117716 | DM 56 | AUS | Bangladesh |
| 254 | 117717 | DM 59 | AUS | Bangladesh |
| 255 | 117719 | Doble Carolina Rinaldo Barsani | ADMIX | Uruguay |
| 256 | 117721 | Dom-Sofid | AROMATIC | Iran |
| 257 | 117266 | Dular | AUS | India |
| 258 | 117724 | DV 123 | AUS | Bangladesh |
| 259 | 117726 | DZ 193 | AUS | Bangladesh |
| 260 | 117729 | Edith | TRJ | United States |
| 261 | 117731 | EMATA A 16-34 | IND | Myanmar |
| 262 | 117741 | Ghorbhai | AUS | Bangladesh |
| 263 | 117743 | Goria | AUS | Bangladesh |
| 264 | 117613 | Habiganj Boro 6 | ADMIX | Bangladesh |
| 265 | 117765 | Jamir | AUS | Bangladesh |
| 266 | 117767 | JC 117 | IND | India |
| 267 |  | Kachilon | AUS | Bangladesh |
| 268 | 117774 | Kalubala Vee | AUS | Sri Lanka |
| 269 | 117778 | Karkati 87 | AUS | Bangladesh |
| 270 | 117783 | Khao Tot Long 227 | AUS | Thailand |
| 271 | 117785 | Kihogo | TEJ | Tanzania |
| 272 | 117624 | Kiuki No. 46 | TEJ | Japan |
| 273 | 117791 | KPF-16 | ADMIX | Bangladesh |
| 274 | 117798 | Lady Wright Seln | TRJ | United States |
| 275 | 117799 | Lambayeque 1 | AROMATIC | Peru |
| 276 | 117805 | Llanero 501 | TRJ | Venezuela |
| 277 | 117806 | Lomello | TEJ | Thailand |
| 278 | 117809 | M-202 | ADMIX | United States-CA |
| 279 | 117271 | Minghui 63 | IND | China |
| 280 | 117272 | Moroberekan | TRJ | Guinea |
| 281 |  | N 22 | AUS | India |
| 282 | 117274 | Nipponbare | TEJ | Japan |
| 283 | 117821 | Nira | IND | United States |
| 284 | 117827 | Okshitmayin | ADMIX | Myanmar |
| 285 | 117839 | Palmyra | ADMIX | United States |
| 286 | 117847 | Paung Malaung | AUS | Myanmar |
| 287 |  | PR 304 | TRJ | Puerto Rico |
| 288 | 117856 | PTB 30 | AUS | India |
| 289 | 117857 | R 101 | TRJ | Zaire |
| 290 | 117862 | Rikuto Norin 21 | ADMIX | Japan |
| 291 | 117871 | Sabharaj | IND | Bangladesh |
| 292 | 117276 | Sadu Cho | IND | Korea |
| 293 | 117875 | Sanbyang-Daeme | ADMIX | Korea |
| 294 | 117876 | Santhi Sufaid | AUS | Pakistan |
| 295 | 117878 | Sathi | AUS | Pakistan |
| 296 | 117885 | Shirkati | AUS | Afghanistan |
| 297 | 117886 | Shirogane | TEJ | Japan |
| 298 | 117892 | Sitpwa | TEJ | Myanmar |
| 299 | 117896 | Sufaid | AUS | Pakistan |
| 300 | 117899 | Surjamkuhi | AUS | India |
| 301 | 117908 | Tainan-Iku No. 512 | TEJ | Taiwan |
| 302 | 117279 | Tainung 67 | TEJ | Taiwan |
| 303 |  | Uzbekskij 2 | TEJ | Uzbekistan |
| 304 | 117930 | Victoria F.A. | ADMIX | Argentina |
| 305 | 117938 | WIR 3764 | TEJ | Uzbekistan |
| 306 | 117940 | Yodanya | IND | Myanmar |
| 307 | 117751 | HSIA-CHIOH-KEH-TU | IND | Chinese Taipei (Taiwan) |
| 308 |  | 205 |  |  |
| 309 |  | ARC 10177 |  |  |
| 310 |  | ARC 10352 |  |  |
| 311 |  | ASWINA |  |  |
| 312 |  | BASMATI 1 |  |  |
| 313 |  | CHAHORA 144 |  |  |
| 314 |  | DA16 |  |  |
| 315 |  | GHARIB |  |  |
| 316 |  | IR 64-21 |  |  |
| 317 |  | JUMBO JET |  |  |
| 318 |  | KHAO HAWM |  |  |
| 319 |  | MIRITI |  |  |
| 320 |  | MOJITO COLORADO |  |  |
| 321 |  | NPE 844 |  |  |
| 322 |  | POKKALI |  |  |
| 323 |  | RAYADA |  |  |
| 324 |  | SWARNA |  |  |

**Table S2.** List of a subset of 197 lines from 3KRG panel used in this study.

| No | IRGC Acc No | Line | Subpopulation | Origin |
| --- | --- | --- | --- | --- |
| 3K3G 1^st^ batch  1 |  | Zhenshan 97 B | ind1A | China |
| 2 |  | Minghui 63 | indx | China |
| 3 |  | Azucena | trop1 | Philippines |
| 4 |  | Vandana | indx |  |
| 5 | 125907 | Suweon 311 | ind1B | Korea, Republic of |
| 6 | 125954 | Bohoto Baloochestan | indx | Iran |
| 7 | 125737 | Ex Maraba Guruku | ind1A | Nigeria |
| 8 | 125917 | Tamashiro Hikari | indx | Japan |
| 9 | 125739 | Facagro 64 | ind2 | Burundi |
| 10 | 125871 | Pin Gaew 56 | ind3 | Thailand |
| 11 | 126003 | RD 15 | ind3 | Thailand |
| 12 | 125755 | Gui Hua Zao | indx | China |
| 13 | 125723 | Da Nuo (Zhan) | ind1A | China |
| 14 | 125726 | Deng Deng Qi | ind1A | China |
| 15 | 125925 | Tong Gu Hong | indx | China |
| 16 | 127031 | Altamira 9 | indx | Nicaragua |
| 17 | 121119 | Sican | indx | Peru |
| 18 | 127030 | 3210 | ind1B | Sri Lanka |
| 19 | 127050 | Iniap 6 | indx | Ecuador |
| 20 | 125969 | INIAP 10 | indx | Ecuador |
| 21 | 127049 | Hp 3319-2Wx-6-4-1-B | indx | Korea, Republic of |
| 22 | 126024 | IR 52718-B-B-6-B-B-1-1 | indx | Philippines |
| 23 | 125986 | Ir 80310-12-B-1-3-B | indx | Philippines |
| 24 | 125990 | IRGA 411-1-6-1F-A | ind1B | Colombia |
| 25 | 121160 | YN 1353-3 | indx | Myanmar |
| 26 | 128193 | Tp Mil 53 | indx | Philippines |
| 27 | 127698 | Pachchaiperumal 2462-11 | ind2 | Sri Lanka |
| 28 | 127419 | Hsieh Dau | ind1A | China |
| 29 | 127992 | Bello | ind2 | India |
| 30 | 127645 | Nan Teo 14 | ind1A | China |
| 31 |  | Alagusamba | ind2 | Sri Lanka |
| 32 | 127163 | ARC 6052 | ind2 | India |
| 33 | 127164 | ARC 6218 | indx | India |
| 34 | 127168 | ARC 7236 | ind2 | India |
| 35 | 127212 | Barkhe Tauli | ind1A | Nepal |
| 36 | 127131 | ARC 10894 | indx | India |
| 37 |  | Khao Phae Hom | indx | Lao People's Democratic Republic |
| 38 | 127987 | Bandang Bungkuaklan | ind3 | Indonesia |
| 39 | 127424 | Hurang Ariso Luta | indx | Brazil |
| 40 |  | Co 36 | indx | India |
| 41 | 127924 | Ai Nan Tsao 39 | ind1A | China |
| 42 | 128093 | Kwang Lu Ai 4 | ind1A | China |
| 43 |  | Sadu Cho | trop1 | Liberia |
| 44 | 127471 | K 17-9-1-1 | indx | India |
| 45 | 128015 | Chitraj 14-134 | ind2 | Bangladesh |
| 46 | 127563 | Ligungtung | indx | Philippines |
| 47 | 128523 | Liong Orn | ind3 |  |
| 48 | 128001 | Br 116-3B-53 | ind2 | Bangladesh |
| 49 |  | RP 1153-20-14 | indx | India |
| 50 |  | Ir 4535-Pp 23-6-8-1 | indx | Philippines |
| 51 | 127441 | IR 9560-2-6-3 | ind1B | Philippines |
| 52 | 127944 | Arc 12757 | indx | India |
| 53 | 127961 | Arc 14737 | ind2 | India |
| 54 | 127156 | ARC 14975 | indx | India |
| 55 | 127157 | Arc 15129 | aus | India |
| 56 | 127152 | ARC 13544 | aus | India |
| 57 | 127449 | Jasure Aus | aus | Bangladesh |
| 58 | 128097 | Lobang (White) | indx | Philippines |
| 59 | 127119 | Amarillo | indx | Philippines |
| 60 | 127647 | Napdai | ind2 | India |
| 61 | 127482 | Kalu T 139 | aus | India |
| 62 | 127230 | Bir Bahadur | ind2 | India |
| 63 | 127126 | AR 133 | indx | India |
| 64 | 127470 | K 15591-4 | indx | India |
| 65 | 127870 | Tv 30 | ind1B | Viet Nam |
| 66 | 127106 | Adiallo | indx | Senegal |
| 67 | 127447 | J 6 Ir 520 (Wc 693) | ind1B | Philippines |
| 68 | 127840 | Tai Zhou Xian | indx | China |
| 69 | 127222 | Bhata Pyagi | ind2 | India |
| 70 | 127122 | Anadi White | ind1A | Nepal |
| 71 | 127659 | Ncs 477 | ind2 | India |
| 72 | 127196 | B 3913 B 16-20 St 28 | indx | Indonesia |
| 73 | 127464 | Jin Hua 258 | ind1A | China |
| 74 | 127771 | Rong Dao 4 | ind1A | China |
| 75 |  | Ob Chuey | ind3 | Thailand |
| 76 | 128467 | Shangyipa | trop2 | Bhutan |
| 77 | 127306 | Daw Nok Kaen | ind3 | Thailand |
| 78 | 127450 | Jati Mani | indx | Indonesia |
| 79 | 128514 | Zuihou | temp | Japan |
| 80 | 127753 | Qing Er Xiao 2 | indx | China |
| 81 | 127803 | Si Chao 1 | ind1A | China |
| 82 | 127595 | Malagkit (Pinelipe) | trop1 | Philippines |
| 83 | 127872 | Ugaga | indx | Ghana |
| 84 | 127114 | Ai Jiao Ao Fan Zi | indx | China |
| 85 | 127123 | An Fu Zhan | ind1A | China |
| 86 | 127378 | Gao Jiao Ying Gan Zhan | indx | China |
| 87 |  | Aus 78-125 | ind3 | Gambia |
| 88 | 128289 | E Zi 32 | ind1A | China |
| 89 | 127365 | E Zi 100 | ind1B | China |
| 90 | 127101 | 78 Xuan Wu | ind1A | China |
| 3KRG 2^nd^ batch |  |  |  |  |
| 91 | 127175 | Asfala | ind3 | Kenya |
| 92 | 128246 | Bueng Mong Leng We | trop2 | Thailand |
| 93 | 127202 | Bai Mi Zai 7 | ind1A | China |
| 94 | 127276 | Chi Gu | ind1A | China |
| 95 | 127413 | Hong Du Bai | ind1A | China |
| 96 | 127569 | Liu Li You | ind1A | China |
| 97 | 127125 | An Nan Zao | ind1A | China |
| 98 | 127203 | Bai Ri Xian | ind1A | China |
| 99 | 127908 | Yun Nan Zhan | ind1A | China |
| 100 | 128504 | Xia Hong Gu | ind1A | China |
| 101 | 127435 | IR 19661-364-1-2-3 | ind1B | Philippines |
| 102 | 126957 | Fedearroz 50 | ind1B | Colombia |
| 103 | 126968 | Sanhuangzhan No 2 | indx | China |
| 104 | 126962 | IR 4630-22-2-5-1-3 | indx | Philippines |
| 105 | 126961 | IR 45427-2B-2-2B-1-1 | indx | Philippines |
| 106 | 126960 | IR 07F287 | indx | Philippines |
| 107 | 126964 | IR 77298-14-1-2-10 | ind1B | Philippines |
| 108 | 126966 | IRRI 146 | ind1B | Philippines |
| 109 | 126967 | Jinbubyeo | temp | Korea, Republic of |
| 110 | 126963 | IR 73571-3B-11-3-K2 | indx | Philippines |
| 111 | 124392 | Gambiaka | ind3 | Burkina Faso |
| 112 | 124380 | Botohavana 139 | ind2 | Madagascar |
| 113 | 121990 | Botra Maitso | ind2 | Madagascar |
| 114 | 122011 | Cica 8 | ind1B | Colombia |
| 115 | 122029 | Danau Laut Tawar | indx | Indonesia |
| 116 | 122088 | IR 1561-228-3-3 | indx | Philippines |
| 117 | 122092 | IR 2307-247-2-2-3 | ind1B | Philippines |
| 118 | 122099 | IR 57924-24 | indx | Philippines |
| 119 | 122140 | Kogoni 91-1 | indx | Mali |
| 120 | 124407 | Madinika 1329 | ind2 | Madagascar |
| 121 | 122220 | Rojokely | ind2 | Madagascar |
| 122 | 122254 | Som Cau 70 A | indx | Viet Nam |
| 123 | 124426 | Tsipala B 160 | ind2 | Madagascar |
| 124 | 122284 | WAS 170-B-B-1-1 | ind1B | Senegal |
| 125 | 122285 | WAS 173-B-B-6-2-2 | ind1B | Senegal |
| 126 | 122286 | WAS 174-B-3-5 | indx | Senegal |
| 127 | 122287 | Was 198-B-3-1-3 | ind1B | Senegal |
| 128 | 122288 | WAS 199-B-1-2-1 | ind1B | Senegal |
| 129 | 122289 | Was 203-B-B-2-4-1 | ind1B | Senegal |
| 130 | 122290 | WAS 206-B-B-2-2-1 | ind1B | Senegal |
| 131 | 122291 | WAS 21-B-B-20-4-3-3 | ind1B | Senegal |
| 132 | 122292 | WAS 33-B-B-15-1-4-5 | ind1B | Senegal |
| 133 | 122111 | IR 72967-12-2-3 | ind1B | Philippines |
| 134 | 122115 | IRAT 112 | trop1 | Cote d'Ivoire |
| 135 | 122165 | Medusa | ind1B | Italy |
| 136 | 122184 | Orione | indx | Italy |
| 137 | 121966 | Ariana | indx | Romania |
| 138 | 122017 | CNA 4081 | ind1B | Brazil |
| 139 | 121959 | A 201 | trop1 | United States of America |
| 140 | 122060 | Giza 178 | indx | Egypt |
| 141 | 122168 | Merle | indx | France |
| 142 | 126002 | Pokkali | ind2 | Sri Lanka |
| 143 | 117430 | Avo | indx | Madagascar |
| 144 | 126008 | Surmatiya | ind2 | India |
| 145 | 117494 | JC 1 | aro | India |
| 146 | 126298 | ARC 15091 | indx | India |
| 147 | 126115 | Chang Le San Shu Zao | ind1A | China |
| 148 | 125627 | UPRH 233 | ind1A | India |
| 149 | 126261 | Puillipina Katari | ind2 | Bangladesh |
| 150 | 126240 | Makalioka Standard | ind2 | Madagascar |
| 151 | 117537 | Nep Hoa Vang | japx | Viet Nam |
| 152 | 124432 | Da 7 | ind2 | Bangladesh |
| 153 | 126209 | Hnanwa | ind3 | Myanmar |
| 154 | 126000 | Nirguni | ind2 | India |
| 155 | 125738 | E Zi 96 | ind1A | China |
| 156 | 125869 | Patalasafed Sunghawado | ind2 | India |
| 157 | 127034 | Arc 18061 | indx | India |
| 158 | 125744 | Fu Zao Xian | ind1A | China |
| 159 | 126974 | Bucayab | ind3 | Philippines |
| 160 | 125831 | Mae Mai Lud Ni | ind3 | Thailand |
| 161 | 125681 | Bhojon Kolpo | ind2 | Bangladesh |
| 162 | 125826 | Leuang Yai 344 | ind3 | Thailand |
| 163 | 125822 | Leuang 28-1-87 | indx | Thailand |
| 164 | 126138 | Leuang Yai 2 B 72 | ind3 | Thailand |
| 165 | 125802 | Khao Leuang Rai | ind2 | Thailand |
| 166 | 125803 | Khao Mon | indx | Myanmar |
| 167 | 126014 | Xi Gan Jing Ren | indx | China |
| 168 | 126013 | Wp 65 | ind1B | Thailand |
| 169 | 125747 | Gam Pai 30-12-15 | ind3 | Thailand |
| 170 | 125931 | Vary Malady Mena | ind2 | Madagascar |
| 171 | 125656 | ARC 5840 | indx | India |
| 172 | 125853 | Ncs 237 | ind2 | India |
| 173 | 126084 | Rpw 9-4 (Ss 1) | indx | India |
| 174 | 125643 | Arc 11857 | indx | India |
| 175 | 126004 | RP 9-4 | ind1B | India |
| 176 | 125662 | B 78-S81 | indx | Brunei Darussalam |
| 177 | 125636 | Arc 10594 | indx | India |
| 178 | 126979 | Holdi Gira | ind2 | Bangladesh |
| 179 | 126129 | Ai Zi Hung | ind1A | China |
| 180 | 121246 | Ai Da | temp | China |
| 181 | 125849 | Nan Te Hao | ind1A | China |
| 182 | 125770 | I Kung Pao | ind1A | Taiwan |
| 183 | 125743 | Fukushima Mochi (Glut) | temp | Japan |
| 184 |  | IRRI 123 |  |  |
| 185 |  | Jinbubyeo |  |  |
| 186 |  | AdaySel |  |  |
| 187 |  | IR 64-21 |  |  |
| 188 |  | IR 87707-445-B-B-B |  |  |
| 189 |  | Li-Jiang-Xin-Tuan-Hei-Gu |  |  |
| 190 |  | NSIC Rc 222 | |  |
| 191 |  | Tainung 67 |  |  |
| 192 |  | Anjali |  |  |
| 193 |  | IR64 |  |  |
| 194 |  | IR74371-70-1-1 |  |  |
| 195 |  | IR87707-446-BBB |  |  |
| 196 |  | MTU1010 |  |  |
| 197 |  | Pokkali |  |  |
|  |  |  |  |  |

**Table S3.** Analysis of variance (ANOVA) for different agronomic and physiological traits in RDP1 at reproductive stage under salt stress of 10 dSm^-1^.

| **Traits** | **Pr(>F)** | | |
| --- | --- | --- | --- |
|  | **Line** | **Rep** | **Treatment** |
| Relative grain yield | <0.001*** | 0.001*** |  |
| Number of filled grains | <0.001*** | 0.383 | <0.001*** |
| Number of unfilled grains | <0.001*** | 0.888 | <0.001*** |
| Percentage filled grains | <0.001*** | 0.39 | <0.001*** |
| Hundred grain weight | <0.001*** | <0.001*** | <0.001*** |
| Panicle length | <0.001*** | 0.154 | <0.001*** |
| Plant height | <0.001*** | 0.157 | <0.001*** |
| Tiller number | <0.001*** | 0.605 | <0.001*** |
| Shoot biomass | <0.001*** | 0.018* | <0.001*** |
| Leaf [Na^+^] | <0.001*** | 0.002** | <0.001** |
| Leaf [K^+^] | <0.001*** | 0.014* | <0.001*** |
| Leaf Na^+^/K^+^ratio | 0.011* | 0.011* | <0.001*** |

Data are means of three replications; *, ** and ***, Significance at the 0.05, 0.01 and 0.001 levels by the Fisher’s least significant difference, respectively.

**Table S4.** Analysis of variance (ANOVA) for different agronomic and physiological traits in 3KRG 1^st^ batch at reproductive stage under salt stress of 10 dSm^-1^.

| **Traits** | **Pr(>F)** | | |
| --- | --- | --- | --- |
|  | **Line** | **Rep** | **Treatment** |
| Relative grain yield | <0.001*** | 0.577 |  |
| Number of filled grains | <0.001*** | 0.437 | <0.001*** |
| Number of unfilled grains | <0.001*** | 0.138 | <0.001*** |
| Percentage filled grains | <0.001*** | 0.033* | <0.001*** |
| Hundred grain weight | <0.001*** | 0.415 | <0.001*** |
| Panicle length | <0.001*** | 0.155 | 0.001*** |
| Plant height | <0.001*** | 0.002** | <0.001*** |
| Tiller number | <0.001*** | 0.101 | 0.005** |
| Shoot biomass | <0.001*** | 0.004** | <0.001*** |
| Leaf [Na^+^] | 0.204 | 0.966 | <0.001 *** |
| Leaf [K^+^] | <0.001*** | <0.001*** | <0.001*** |
| Leaf Na^+^/K^+^ratio | 0.122 | 0.009** | 0.086 |

Data are means of three replications; *, ** and ***, Significance at the 0.05, 0.01 and 0.001 levels by the Fisher’s least significant difference, respectively.

**Table S5.** Analysis of variance (ANOVA) for different agronomic and physiological traits in 3KRG 2^nd^ batch at reproductive stage under salt stress of 10 dSm^-1^.

| **Traits** | **Pr(>F)** | | |
| --- | --- | --- | --- |
|  | **Line** | **Rep** | **Treatment** |
| Relative grain yield | <0.001*** | 0.112 |  |
| Number of filled grains | <0.001*** | 0.202 | <0.001*** |
| Number of unfilled grains | <0.001*** | 0.600 | <0.001*** |
| Percentage filled grains | <0.001*** | 0.136 | <0.001*** |
| Hundred grain weight | 0.001*** | 0.955 | 0.038* |
| Panicle length | <0.001*** | 0.928 | 0.178 |
| Plant height | <0.001*** | 0.838 | <0.001*** |
| Tiller number | <0.001*** | 0.032* | 0.010* |
| Shoot biomass | <0.001*** | 0.030* | 0.056 |
| Leaf [Na^+^] | 0.071. | 0.552 | <0.001*** |
| Leaf [K^+^] | <0.001*** | 0.545 | 0.000*** |
| Leaf Na^+^/K^+^ratio | 0.041* | 0.310 | 0.001*** |

Data are means of three replications; *, ** and ***, Significance at the 0.05, 0.01 and 0.001 levels by the Fisher’s least significant difference, respectively.

**Table S6.** Analysis of variance (ANOVA) for different agronomic and physiological traits in the selected contrasting lines at reproductive stage under salt stress of 10 dSm^-1^.

| **Traits** | **Pr(>F)** | | |
| --- | --- | --- | --- |
|  | **Line** | **Rep** | **Treatment** |
| Relative grain yield | <0.001*** | 0.684 | 0.204 |
| Number of filled grains | <0.001*** | 0.254 | 0.532 |
| Number of unfilled grains | <0.001*** | 0.653 | 0.979 |
| Percentage filled grains | <0.001*** | 0.211 | 0.413 |
| Hundred grain weight | <0.001*** | 0.135 | 0.961 |
| Panicle length | <0.001*** | 0.108 | 0.181 |
| Plant height | <0.001 *** | 0.663 | 0.300 |
| Tiller number | <0.001*** | 0.077 | 0.163 |
| Shoot biomass | <0.001*** | 0.177 | <0.001*** |
| Leaf [Na^+^] | <0.001*** | 0.619 | 0.234 |
| Leaf [K^+^] | 0.068 | 0.013* | 0.827 |
| Leaf Na^+^/K^+^ ratio | <0.001*** | 0.703 | 0.063 |
| Pollen viability | <0.001*** | 0.001*** | 0.409 |

Data are means of three replications; *, ** and ***, Significance at the 0.05, 0.01 and 0.001 levels by the Fisher’s least significant difference, respectively.

**Table S7.** Candidate genes from the 40 kb subregion within the 100 kb window on chromosomes 1 and 11 in which relative grain yield GWAS peaks co-located with multiple salinity-response traits.

| Chr | start | end | Locus ID | Gene Description |
| --- | --- | --- | --- | --- |
| 1 | 25114081 | 25118368 | LOC_Os01g43844 | cytochrome P450 72A1, putative, expressed |
|  | 25119734 | 25121927 | LOC_Os01g43851 | cytochrome P450 72A1, putative, expressed |
|  | 25132885 | 25138147 | LOC_Os01g43870 | NLI interacting factor-like phosphatase, putative, expressed |
|  | 25144495 | 25146560 | LOC_Os01g43890 | OsSCP4 - Putative Serine Carboxypeptidase homologue, expressed |
|  | 25158379 | 25152102 | LOC_Os01g43910 | CGMC_MAPKCMGC_2.4 - CGMC includes CDA, MAPK, GSK3, and CLKC kinases, expressed |
|  | 25187250 | 25189517 | LOC_Os01g43940 | oligopeptide transporter, putative, expressed |
|  | 25226665 | 25233778 | LOC_Os01g44040 | CAS1 domain-containing protein 1 precursor, putative, expressed |
|  | 25234661 | 25238165 | LOC_Os01g44050 | siroheme synthase, putative, expressed |
|  | 25257001 | 25263981 | LOC_Os01g44069 | glycerol-3-phosphate acyltransferase, putative, expressed |
|  | 25271543 | 25273512 | LOC_Os01g44090 | X8 domain containing protein, expressed |
|  | 25281682 | 25286614 | LOC_Os01g44110 | serine/threonine-protein kinase, putative, expressed |
|  | 25291338 | 25298072 | LOC_Os01g44130 | aspartic proteinase oryzasin-1 precursor, putative, expressed |
|  |  |  |  |  |
|  |  |  |  |  |
| 11 | 6925243 | 6927549 | LOC_Os11g12410 | serpin domain containing protein, putative, expressed |
|  | 6940163 | 6942107 | LOC_Os11g12420 | serpin domain containing protein, putative, expressed |
|  | 6963249 | 6964563 | LOC_Os11g12460 | serpin domain containing protein, putative, expressed |
|  | 6975139 | 6977372 | LOC_Os11g12480 | OsFBL54 - F-box domain and LRR containing protein, expressed |
|  | 6989971 | 6986691 | LOC_Os11g12500 | ulp1 protease family, C-terminal catalytic domain containing protein, expressed |
|  | 7005794 | 7007235 | LOC_Os11g12520 | serpin domain containing protein, putative, expressed |
|  | 7018706 | 7011435 | LOC_Os11g12530 | receptor-like protein kinase 5 precursor, putative, expressed |
|  | 7061410 | 7056514 | LOC_Os11g12560 | receptor-like protein kinase HAIKU2 precursor, putative, expressed |
|  |  |  |  |  |
| 11 | 7005794 | 7007235 | LOC_Os11g12520 | serpin domain containing protein, putative, expressed |
|  | 7018706 | 7011435 | LOC_Os11g12530 | receptor-like protein kinase 5 precursor, putative, expressed; drought stress response; heavy metal stress treatment in root |
|  | 7061410 | 7056514 | LOC_Os11g12560 | receptor-like protein kinase HAIKU2 precursor, putative, expressed; time course of salt-stress treatment, weighted gene co-expression network analysis |
|  | 7104084 | 7110745 | LOC_Os11g12620 | receptor protein kinase CLAVATA1 precursor, putative, expressed; meiosis-related genes in rice anther autotetraploid |
|  | 7128857 | 7125124 | LOC_Os11g12650 | PHD-finger domain containing protein, putative, expressed |
|  | 7198552 | 7193230 | LOC_Os11g12740 | peptide transporter PTR2, putative, expressed |
|  |  |  |  |  |
|  |  |  |  |  |
| 11 | 17896685 | 17900238 | LOC_Os11g30740 | transposon protein, putative, CACTA, En/Spm sub-class, expressed |
|  | 17943544 | 17945390 | LOC_Os11g30810 | sulfotransferase domain containing protein, expressed |
|  | 17984964 | 17986719 | LOC_Os11g30910 | sulfotransferase domain containing protein, expressed |
|  | 18071815 | 18070199 | LOC_Os11g31060 | IQ calmodulin-binding and BAG domain containing protein, putative, expressed |
|  | 18092965 | 18095638 | LOC_Os11g31090 | transferase family protein, putative, expressed |
|  | 18102879 | 18096831 | LOC_Os11g31100 | gibberellin response modulator protein, putative, expressed |
|  |  |  |  |  |
| 11 | 28705306 | 28703899 | LOC_Os11g47460 | MYB family transcription factor, putative, expressed |
|  | 28718384 | 28719242 | LOC_Os11g47490 | senescence-induced receptor-like serine/threonine-protein kinase precursor, putative, expressed |
|  | 28724027 | 28723113 | LOC_Os11g47500 | glycosyl hydrolase, putative, expressed |
|  | 28728823 | 28727475 | LOC_Os11g47510 | glycosyl hydrolase, putative, expressed |
|  | 28730145 | 28731323 | LOC_Os11g47520 | glycosyl hydrolase, putative, expressed |
|  | 28733117 | 28734280 | LOC_Os11g47530 | glycosyl hydrolase, putative, expressed |
|  | 28735982 | 28737159 | LOC_Os11g47550 | glycosyl hydrolase, putative, expressed |
|  | 28739616 | 28740710 | LOC_Os11g47560 | glycosyl hydrolase, putative, expressed |
|  | 28748076 | 28749262 | LOC_Os11g47570 | glycosyl hydrolase, putative, expressed |
|  | 28753857 | 28755114 | LOC_Os11g47580 | glycosyl hydrolase, putative, expressed |
|  | 28757160 | 28755786 | LOC_Os11g47590 | glycosyl hydrolase, putative, expressed |
|  | 28760007 | 28758715 | LOC_Os11g47600 | glycosyl hydrolase, putative, expressed |
|  | 28761603 | 28760411 | LOC_Os11g47610 | glycosyl hydrolase, putative, expressed |
|  | 28763222 | 28762443 | LOC_Os11g47620 | ZOS11-09 - C2H2 zinc finger protein, expressed |
|  | 28766937 | 28766092 | LOC_Os11g47630 | ZOS11-10 - C2H2 zinc finger protein, expressed |
|  | 28774435 | 28777987 | LOC_Os11g47650 | trp repressor/replication initiator, putative, expressed |
|  | 28781161 | 28782202 | LOC_Os11g47670 | thaumatin family domain containing protein, expressed |
|  | 28783158 | 28783992 | LOC_Os11g47680 | thaumatin family domain containing protein, expressed |
|  | 28784150 | 28784662 | LOC_Os11g47690 | zinc finger, C3HC4 type domain containing protein, expressed |
|  | 28785275 | 28785787 | LOC_Os11g47700 | zinc finger, C3HC4 type domain containing protein, expressed |
|  | 28788237 | 28785848 | LOC_Os11g47710 | SNF7 domain containing protein, putative, expressed |
|  | 28804248 | 28808550 | LOC_Os11g47760 | DnaK family protein, putative, expressed |
|  | 28809410 | 28811550 | LOC_Os11g47770 | selT/selW/selH selenoprotein domain containing protein, expressed |
|  | 28815723 | 28811740 | LOC_Os11g47780 | pollen signalling protein with adenylyl cyclase activity, putative, expressed |
|  | 28820765 | 28824245 | LOC_Os11g47800 | spo0B-associated GTP-binding protein, putative, expressed |
|  | 28827676 | 28828513 | LOC_Os11g47809 | metallothionein, putative, expressed |
|  | 28842809 | 28838673 | LOC_Os11g47820 | glucan endo-1,3-beta-glucosidase precursor, putative, expressed |
|  | 28852938 | 28845905 | LOC_Os11g47830 | RNA recognition motif containing protein, expressed |
|  | 28857521 | 28853507 | LOC_Os11g47840 | OsRhmbd18 - Putative Rhomboid homologue, expressed |
|  | 28870096 | 28872571 | LOC_Os11g47870 | GRAS family transcription factor domain containing protein, expressed |
|  | 28887633 | 28889549 | LOC_Os11g47890 | GRAS family transcription factor domain containing protein, expressed |
|  | 28891244 | 28893528 | LOC_Os11g47900 | SCARECROW, putative, expressed |
|  | 28895187 | 28896974 | LOC_Os11g47910 | SCARECROW, putative, expressed |

**Table S8.** The most-frequently listed gene ontology terms enriched in different modules for the multi-trait GWAS interval on Chromosome 1.

| **Gene Ontology Term** | **Module** | **Genes** |
| --- | --- | --- |
| apoplast | 242 | LOC_Os05g38410;LOC_Os05g38420 |
|  | 1600 | LOC_Os05g38410;LOC_Os02g12730;LOC_Os05g38420 |
|  | 2580 | LOC_Os01g62490;LOC_Os05g38410;LOC_Os05g38420 |
|  |  |  |
| cutin biosynthethic process | 242 | LOC_Os01g44069 |
|  | 1600 | LOC_Os01g44069 |
|  | 1731 | LOC_Os01g44069 |
|  | 2580 | LOC_Os01g44069 |
|  |  |  |
| lignin catabolic process | 242 | LOC_Os05g38410;LOC_Os05g38420 |
|  | 1600 | LOC_Os05g38410;LOC_Os05g38420 |
|  | 2580 | LOC_Os01g62490;LOC_Os05g38410;LOC_Os05g38420 |


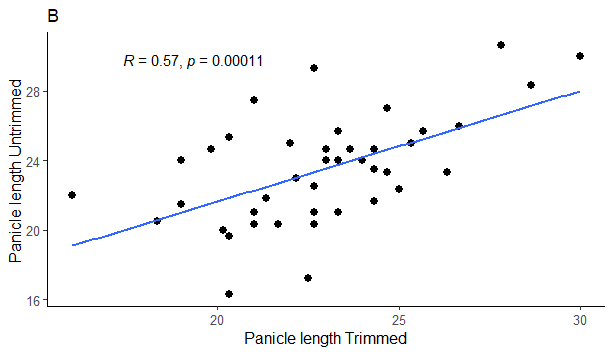

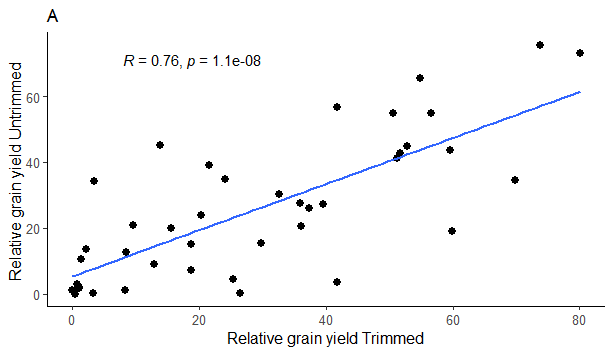


**Figure S1.** Scatterplot of (A) Relative grain yield, (B) Panicle length (C) Number of filled grains, (D) Number of unfilled grains, (E) Hundred grain wt, (F) Pollen viability, (G) Leaf [Na^+^], (H) Leaf [K^+^], (I) Leaf Na^+^/K^+^ ratio, (J) Plant height, (K) Shoot biomass and (L) Tiller number of Trimmed and Untrimmed treatments of the selected contrasting lines at the reproductive stage.


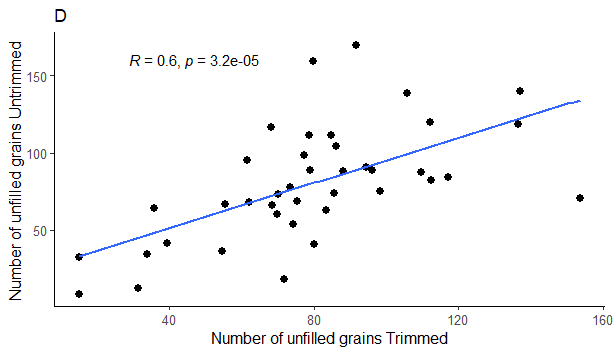

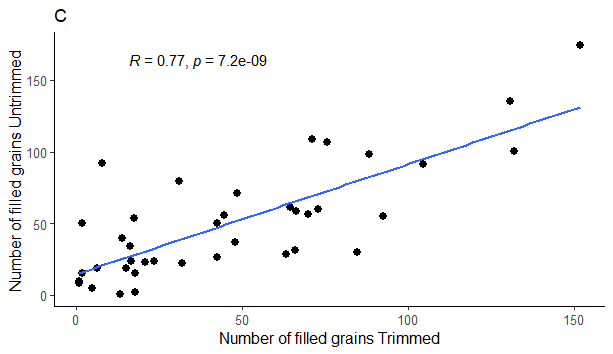


**(Figure S1 continued.)**


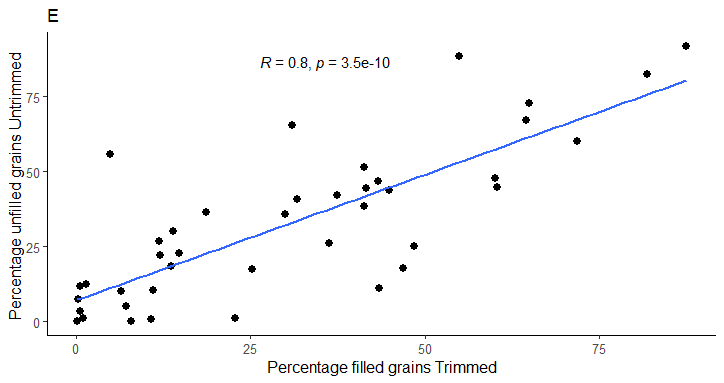


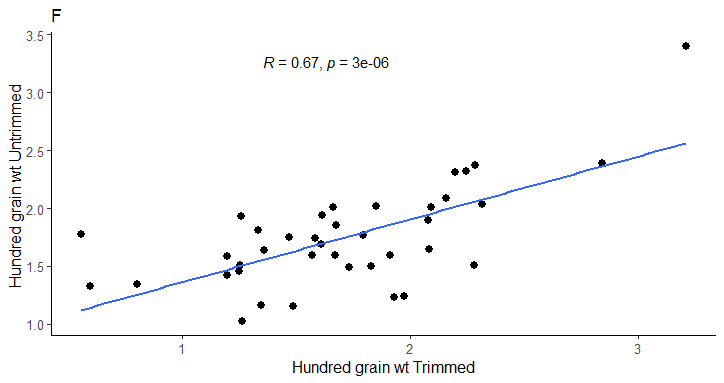


**(Figure S1 continued.)**


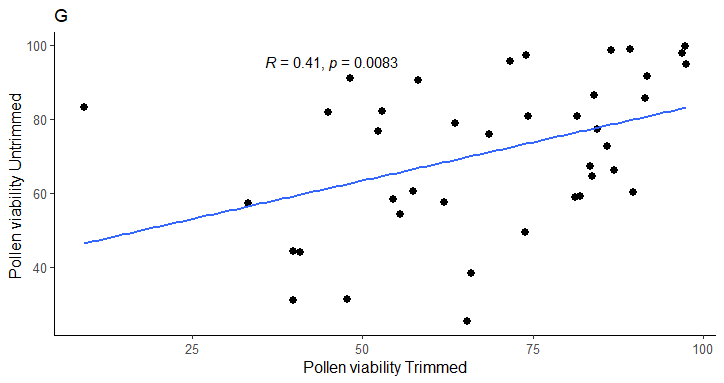


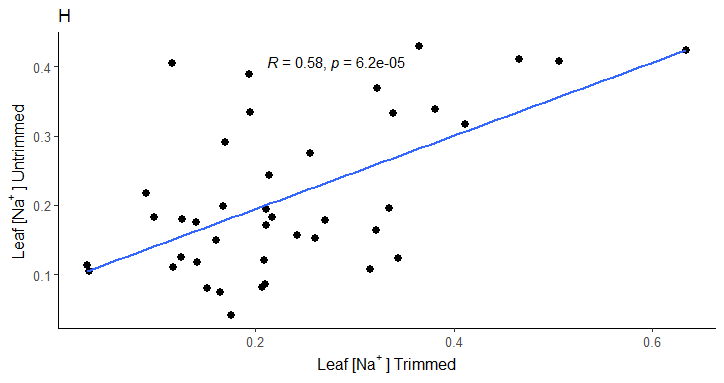
**(Figure S1 continued.)**


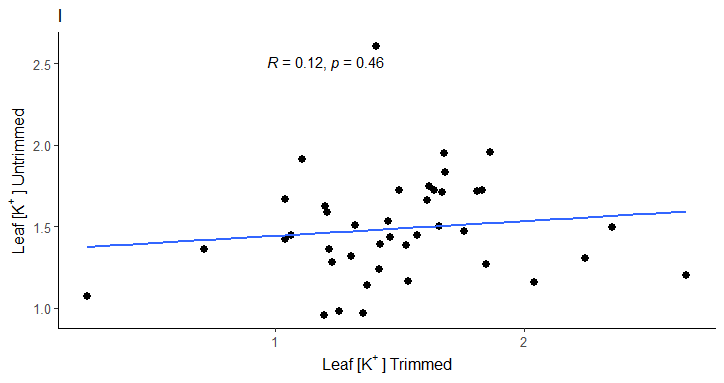
**
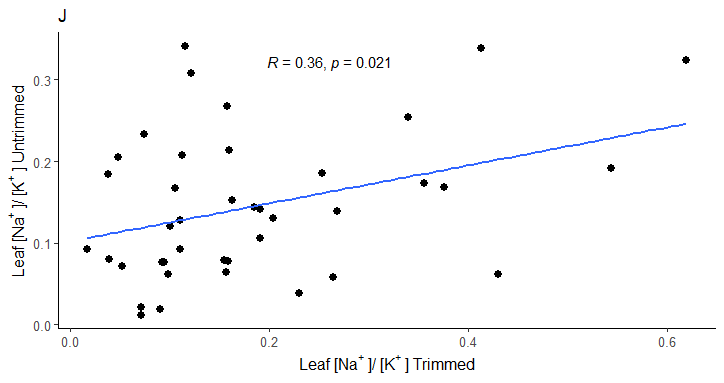
**

**(Figure S1 continued.)**


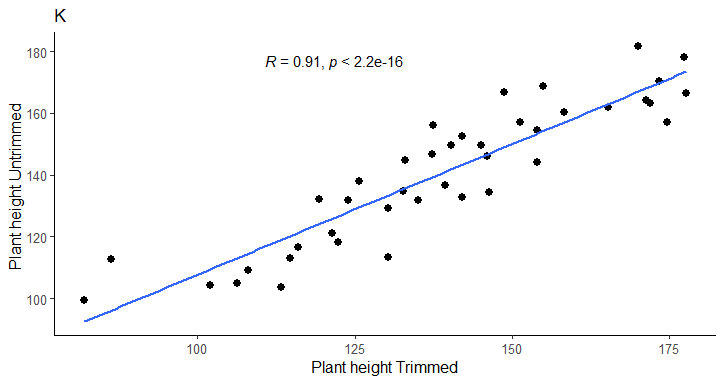


**
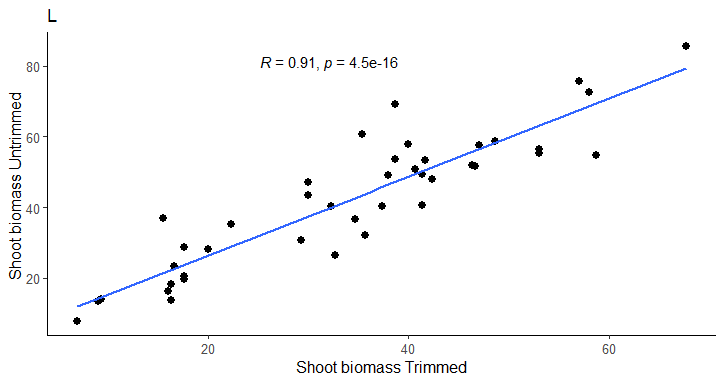
(Figure S1 continued.)**

**
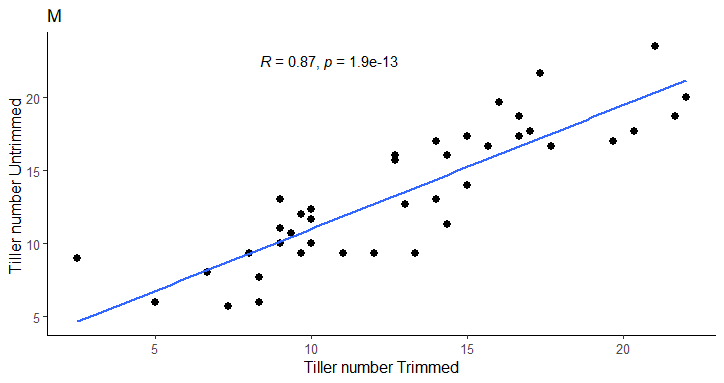
**

**(Figure S1 continued.)**


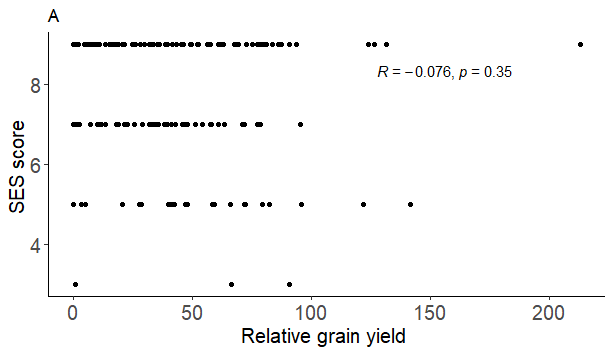


**Figure S2.** Scatterplot of Standard evaluation score (SES) extracted from the SNP-seek database (snp-seek.irri.org) under seedling stage salinity at an EC12 dSm^-1^ and relative grain yield at the reproductive stage of the 3KRG. Entries were scored based on visual symptoms using IRRI’s Standard evaluation system (SES) scores, with ratings from 1 (highly tolerant) to 9 (highly sensitive; IRRI, 2014). SES score of 1 has normal growth, only the old leaves show white tips while no symptoms on young leaves. SES score of 3 has near normal growth, but only leaf tips burn, few older leaves become whitish partially. SES score of 5 has severely retarded growth with most old leaves severely injured and few young leaves elongating. SES score of 7 has complete cessation of growth, most leaves dried and only few young leaves still green. SES score of 9 has almost all plants dead or dying.


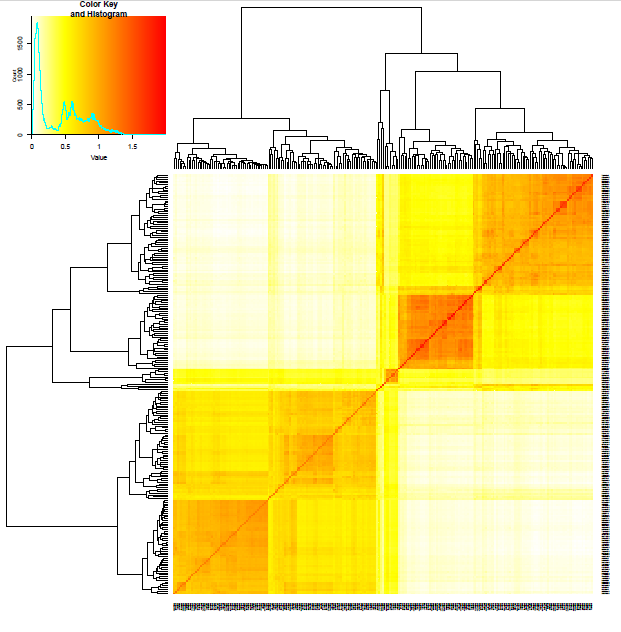

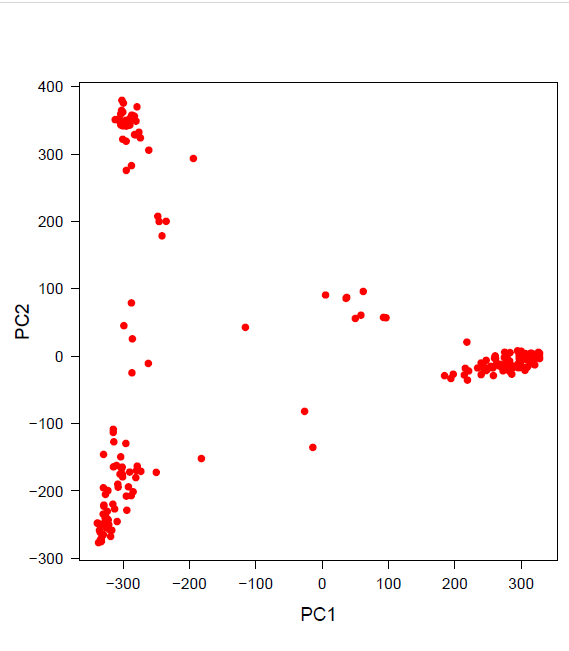


A

B

**Figure S3.** Population structure in the RDP1 panel. (A) Heatmap of the kinship matrix and (B) Scatterplot of the first two principal components (PC) produced by GAPIT.

Relative grain yield


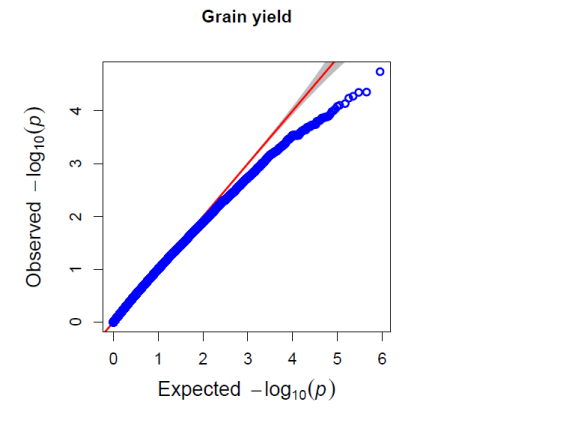


Chromosome


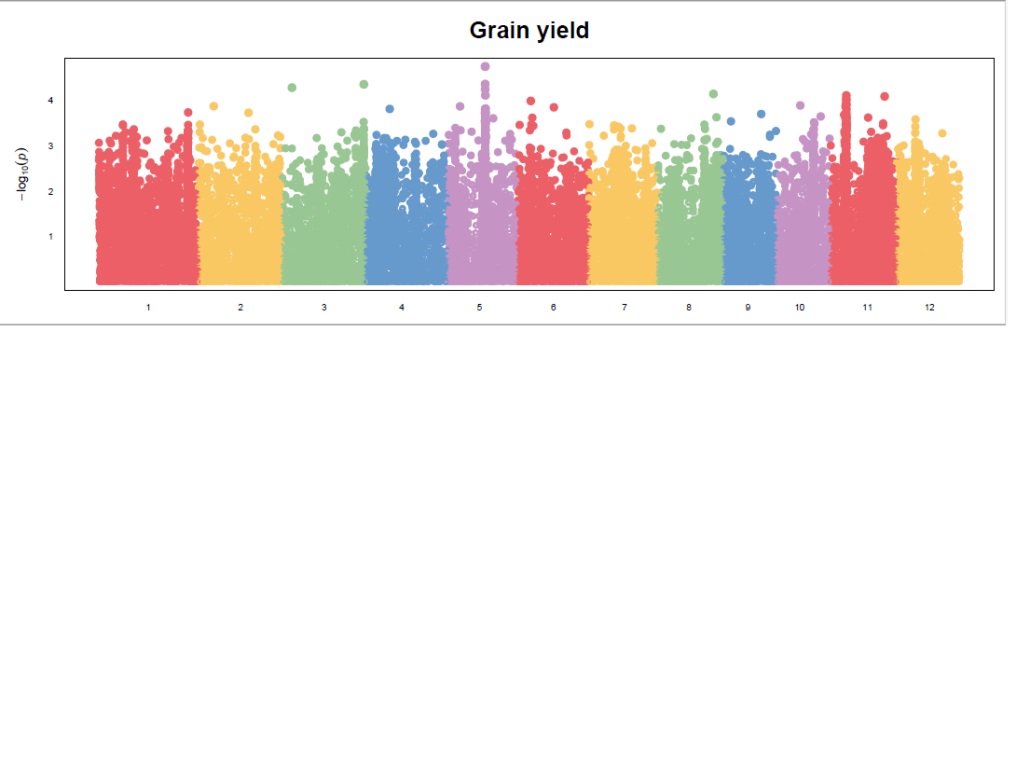


Relative hundred grain weight


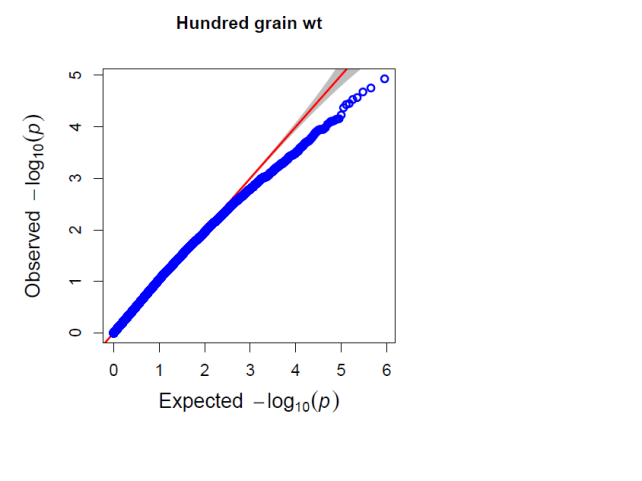


Chromosome


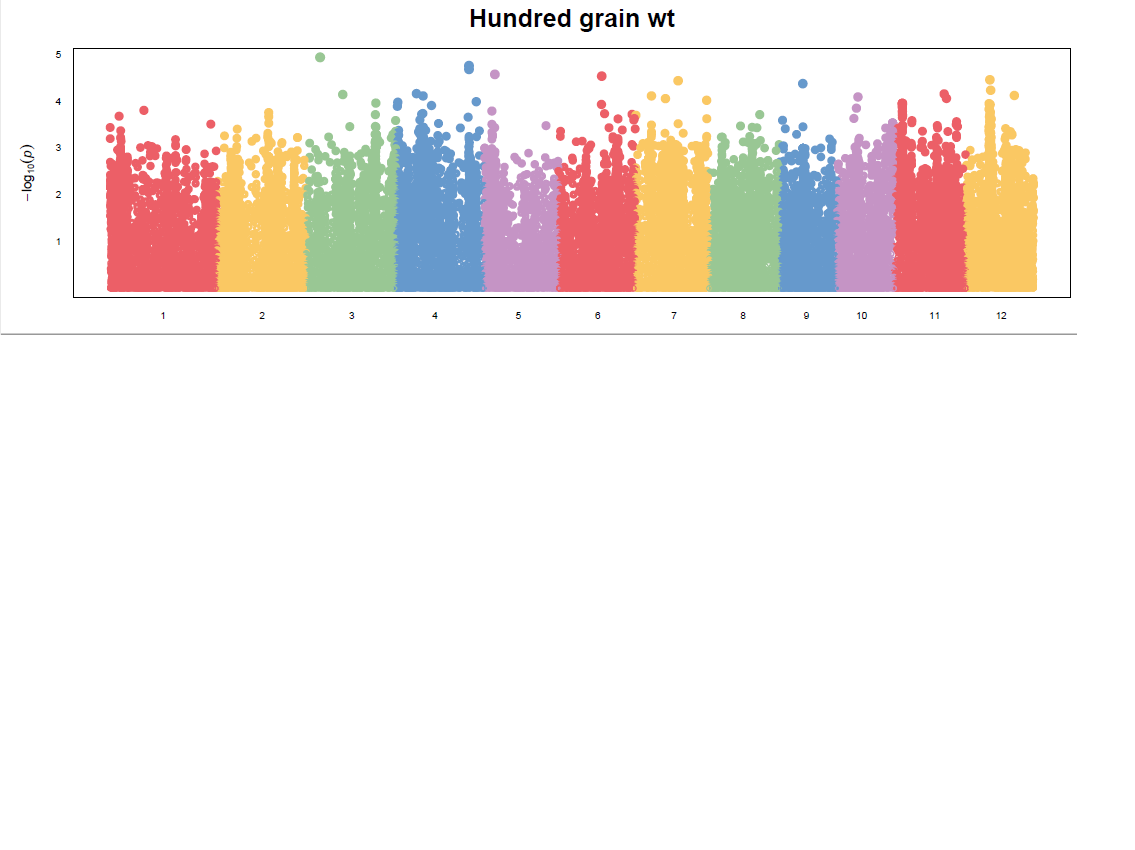


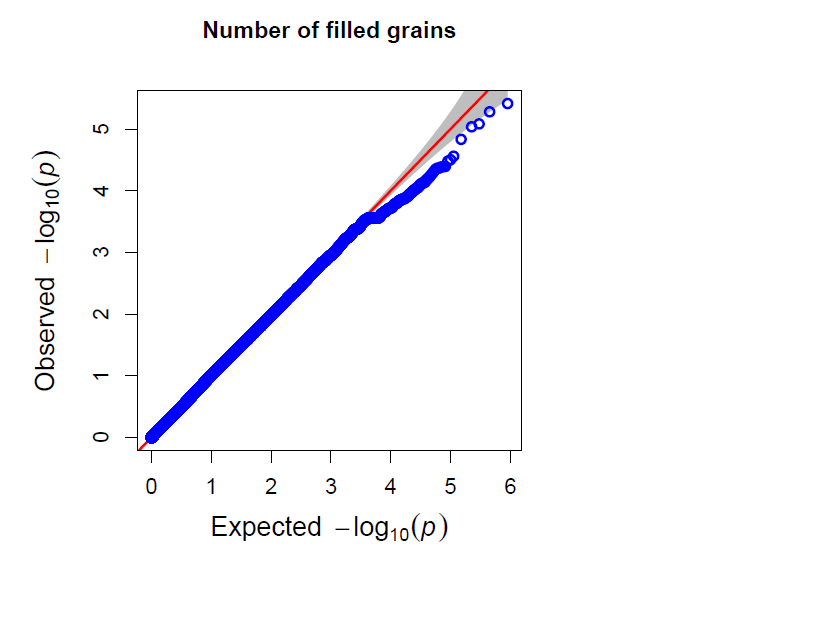


Relative number of filled grains

Chromosome


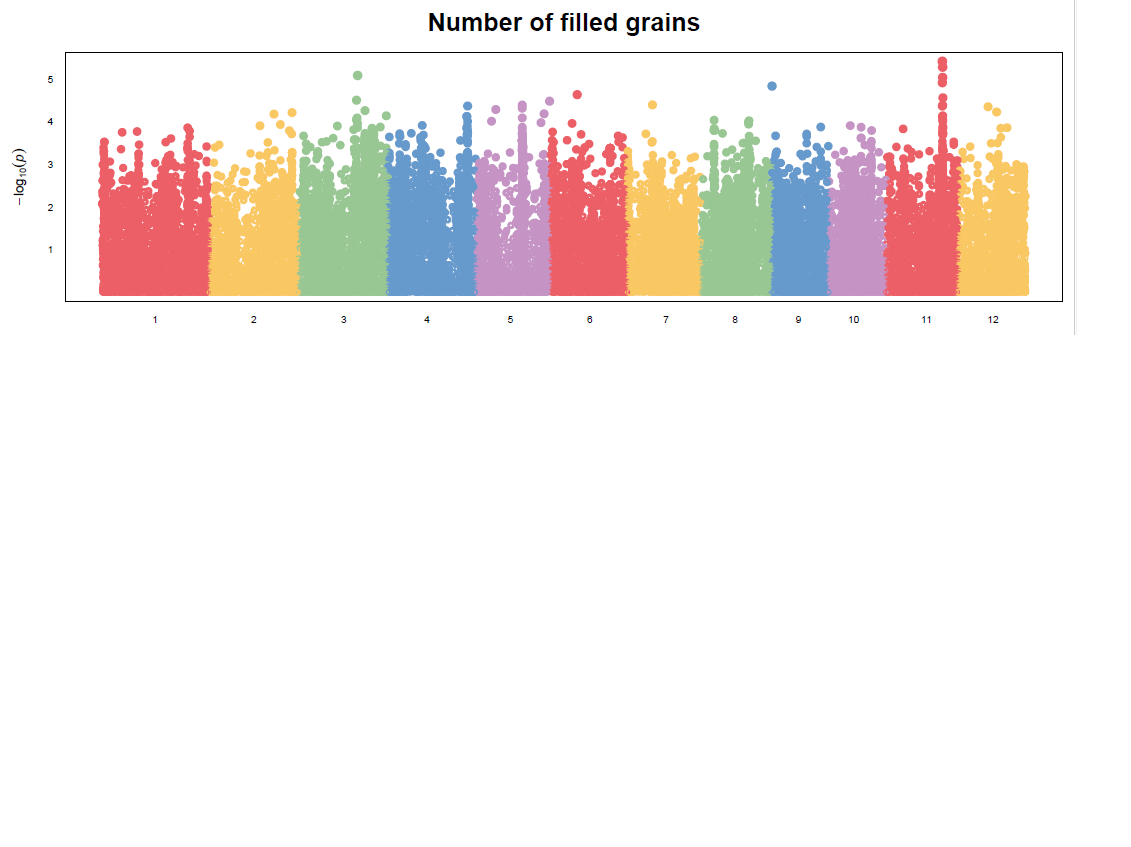


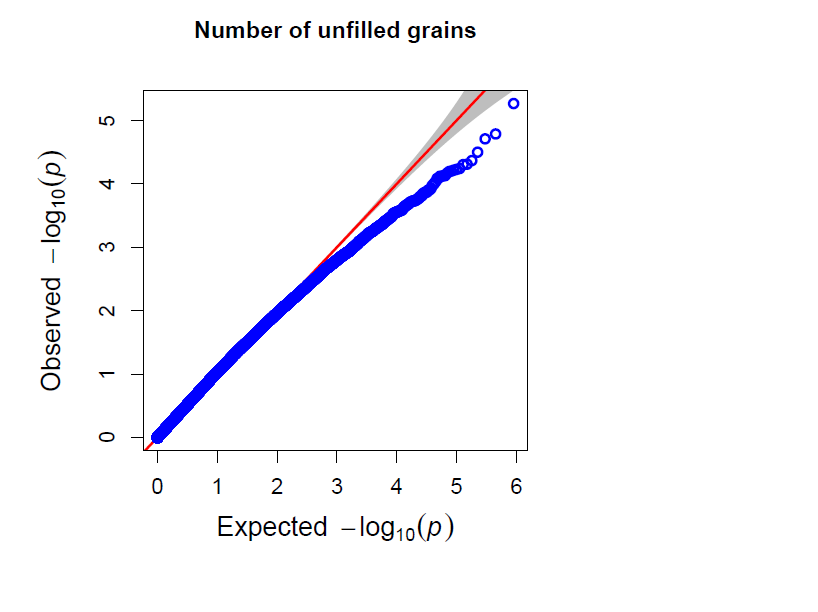


Relative number of unfilled grains

Chromosome


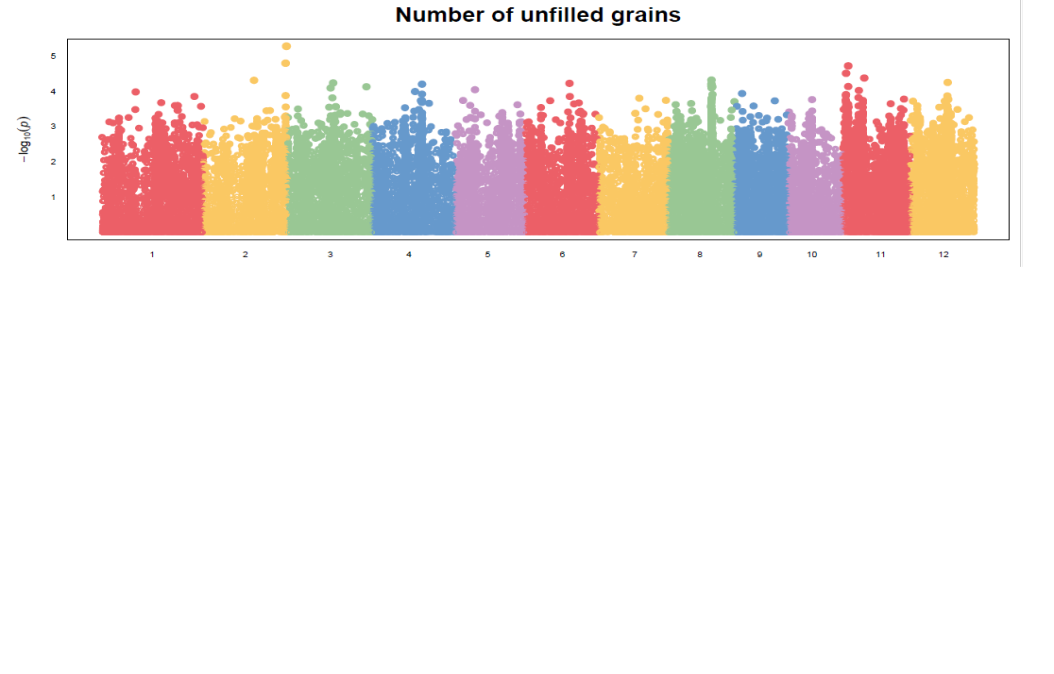


**Figure S4.** Manhattan plot and Quantile-Quantile (QQ) plots of relative grain yield, relative hundred grain weight, relative number of filled grains, relative number of unfilled grains, relative panicle length, relative plant height, relative shoot biomass, relative tiller number, leaf [Na^+^] under salinity, leaf [K^+^] under salinity and leaf Na^+^/K^+^ ratio under salinity using RDP1. Negative log_10_- transformed P values from a genome wide scan are plotted against position on each of the 12 chromosomes. Blue horizontal dashed line indicates the genome wide set threshold.


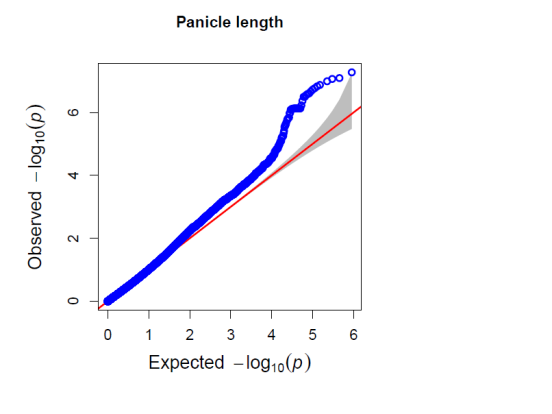

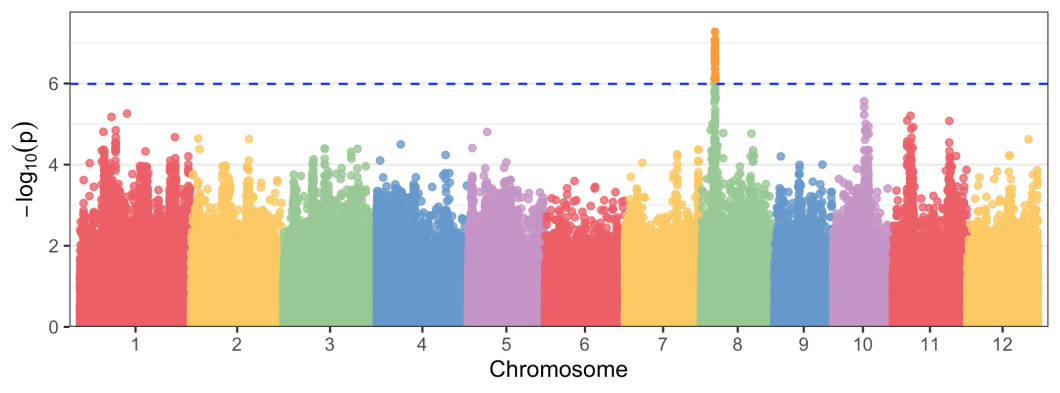


Relative panicle length

Relative plant height


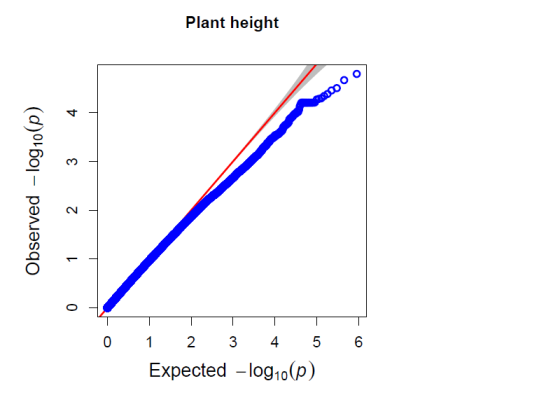


Chromosome


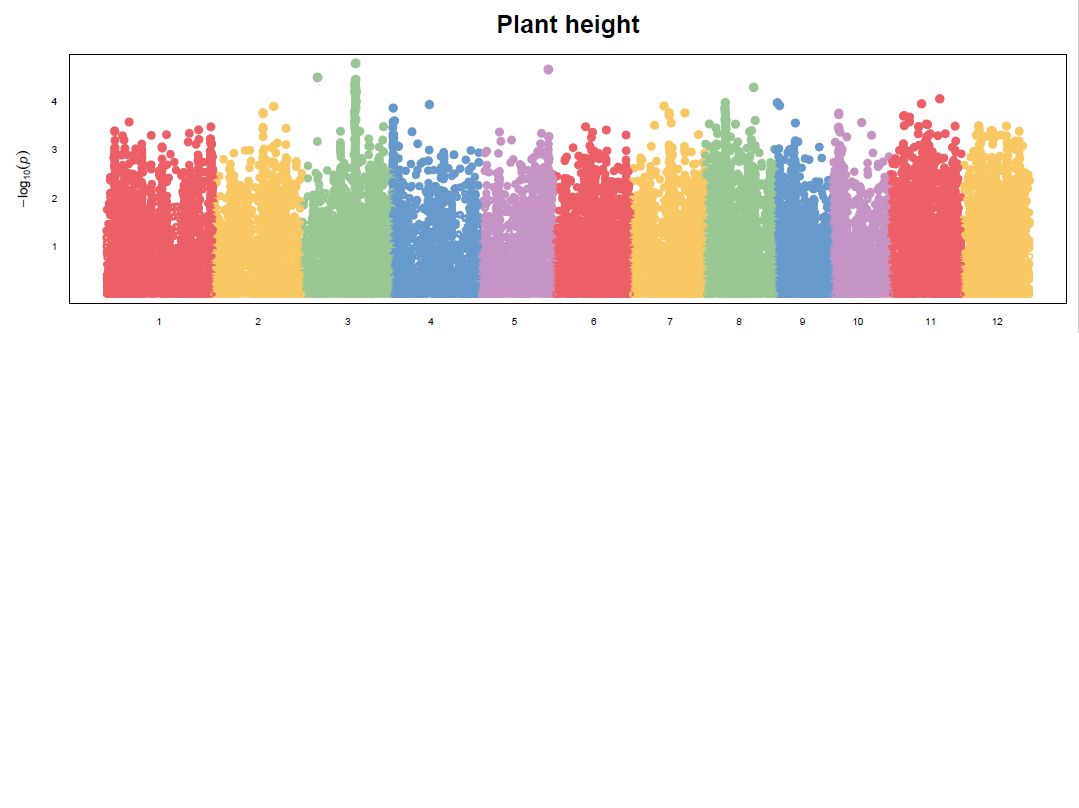


Relative plant height

Relative shoot biomass


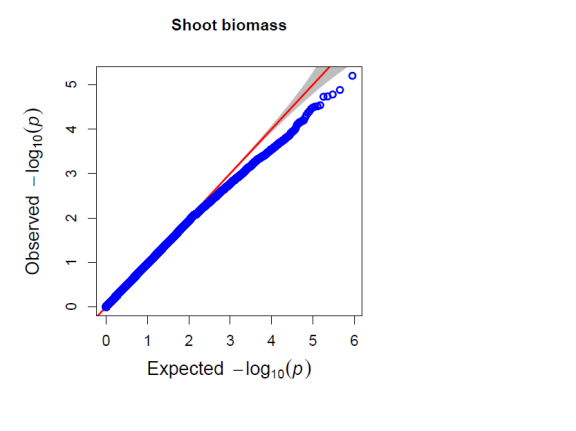


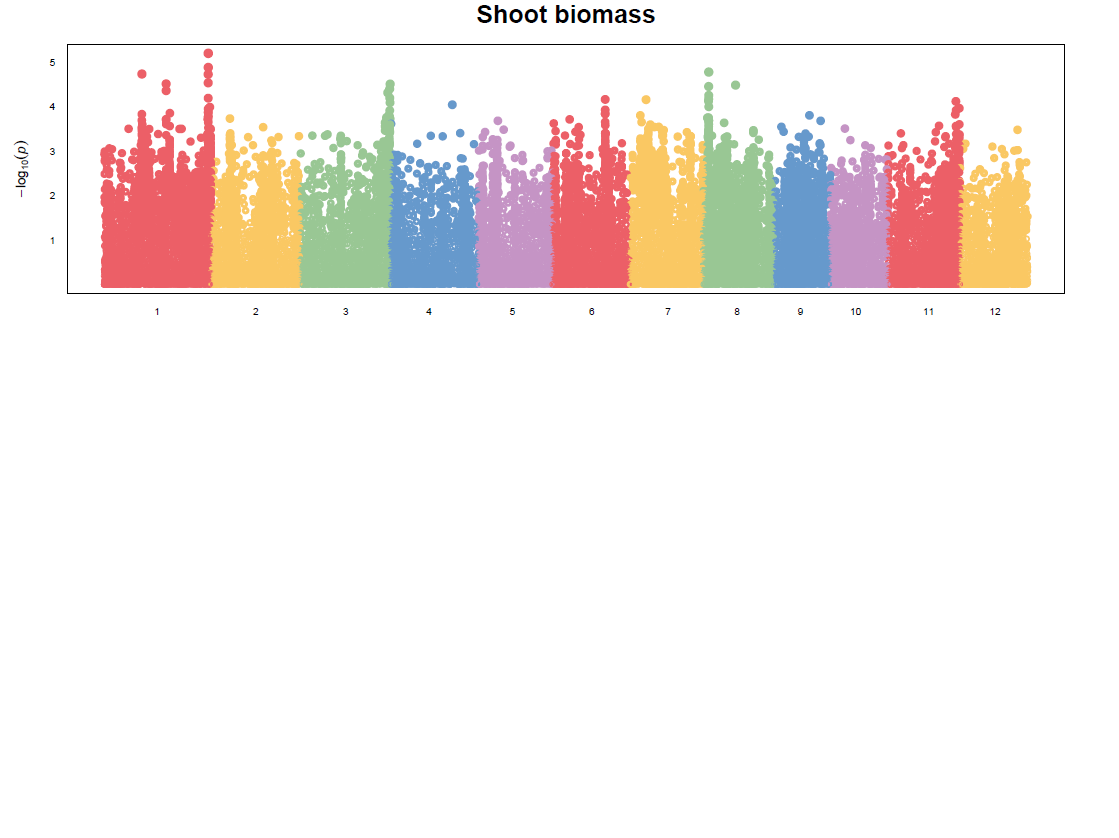


Chromosome

Relative tiller number


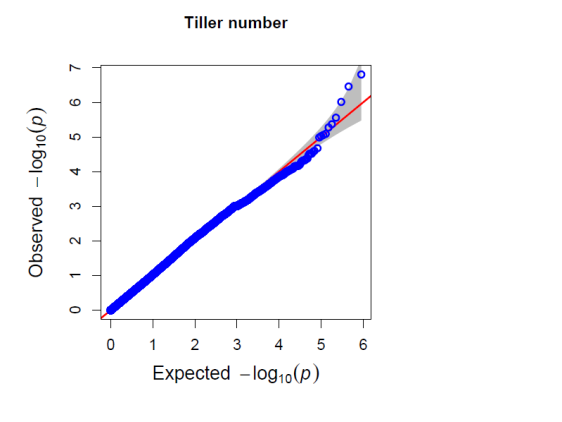

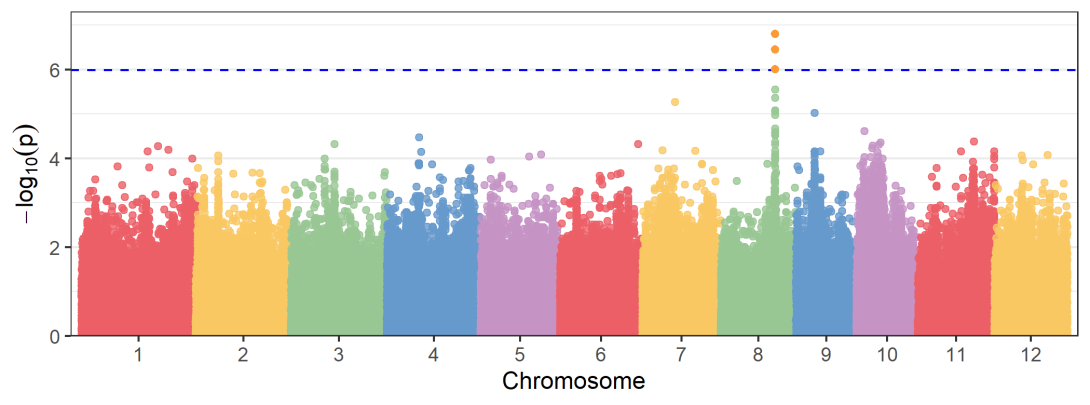


(**Figure S4 continued.)**

Leaf [Na^+^] under salinity


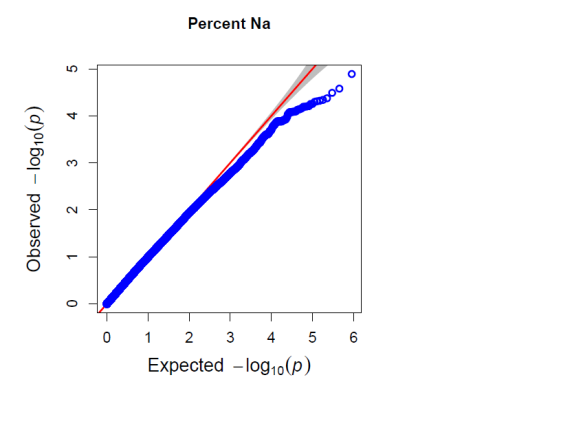


Chromosome


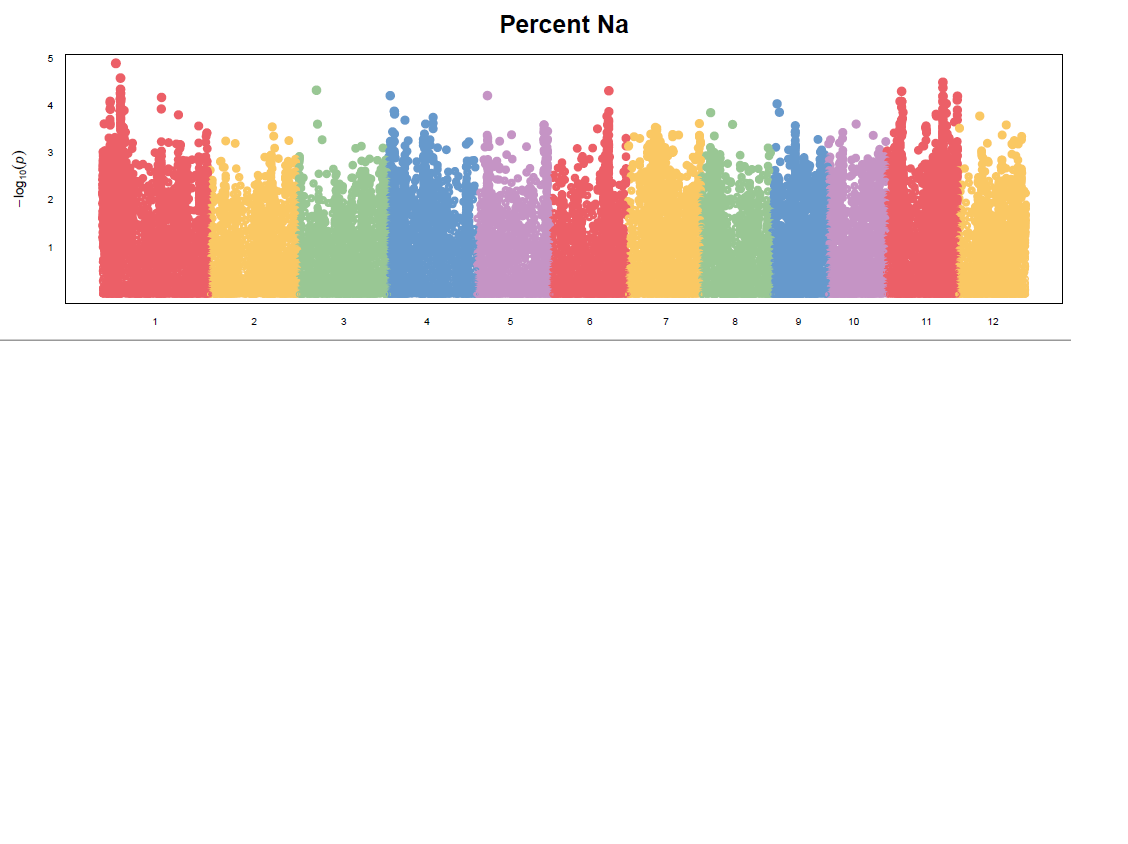


Leaf [K^+^] under salinity


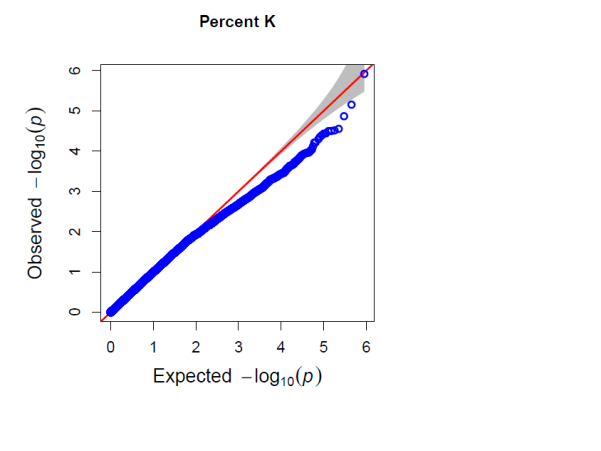


Chromosome


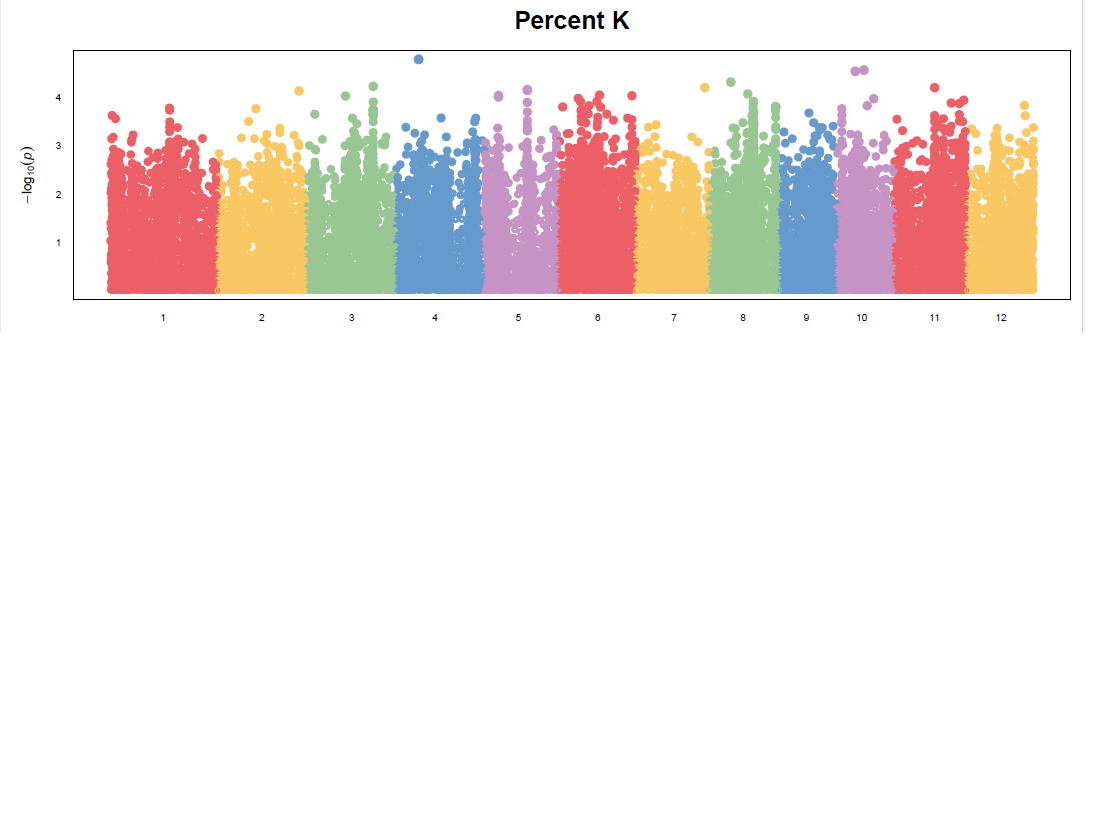


Leaf Na^+^/K^+^ ratio under salinity


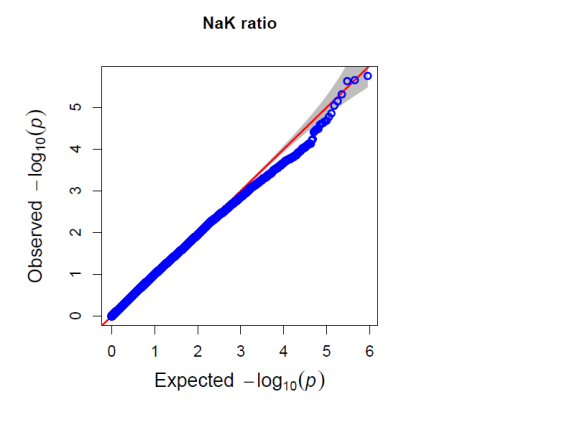


Chromosome


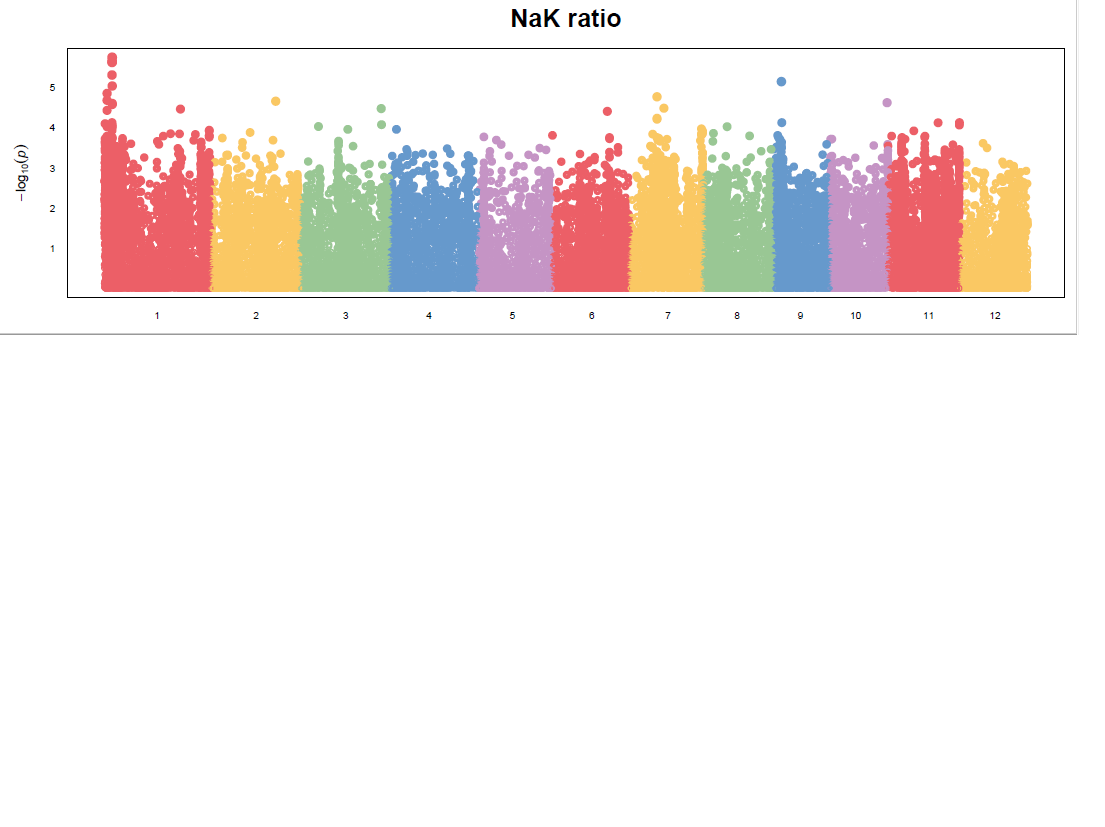


(**Figure S4 continued.)**


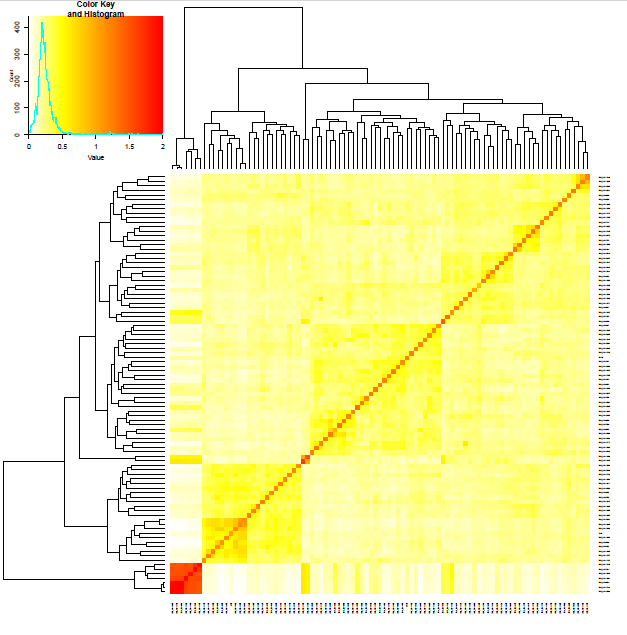

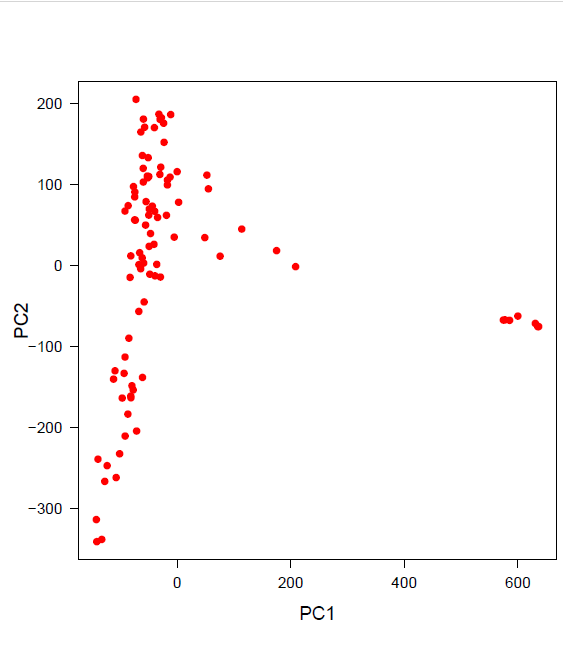


A

B

**Figure S5.** Population structure in the 3KRG 1^st^ batch panel (A) Heatmap of the kinship matrix and (B) Scatterplot of the first two principal components (PC) produced by GAPIT.

Chromosome


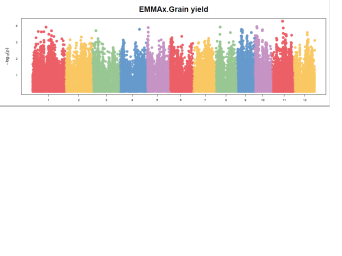


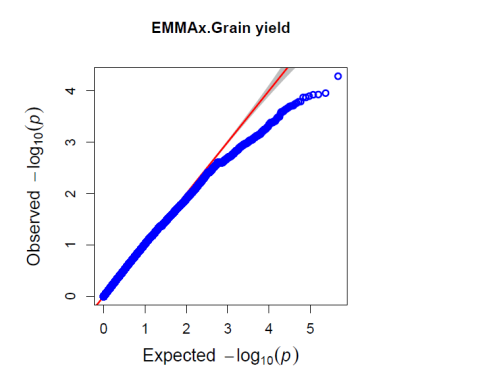


Relative grain yield

Relative hundred grain weight


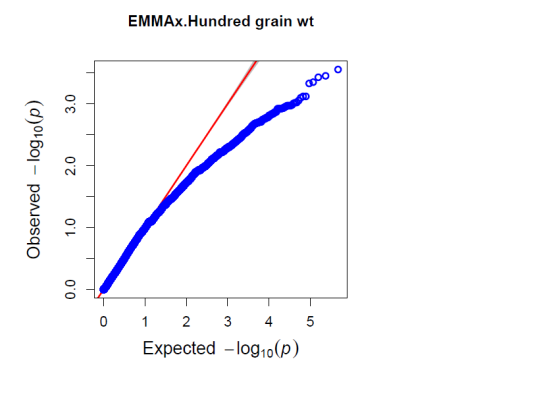


Chromosome


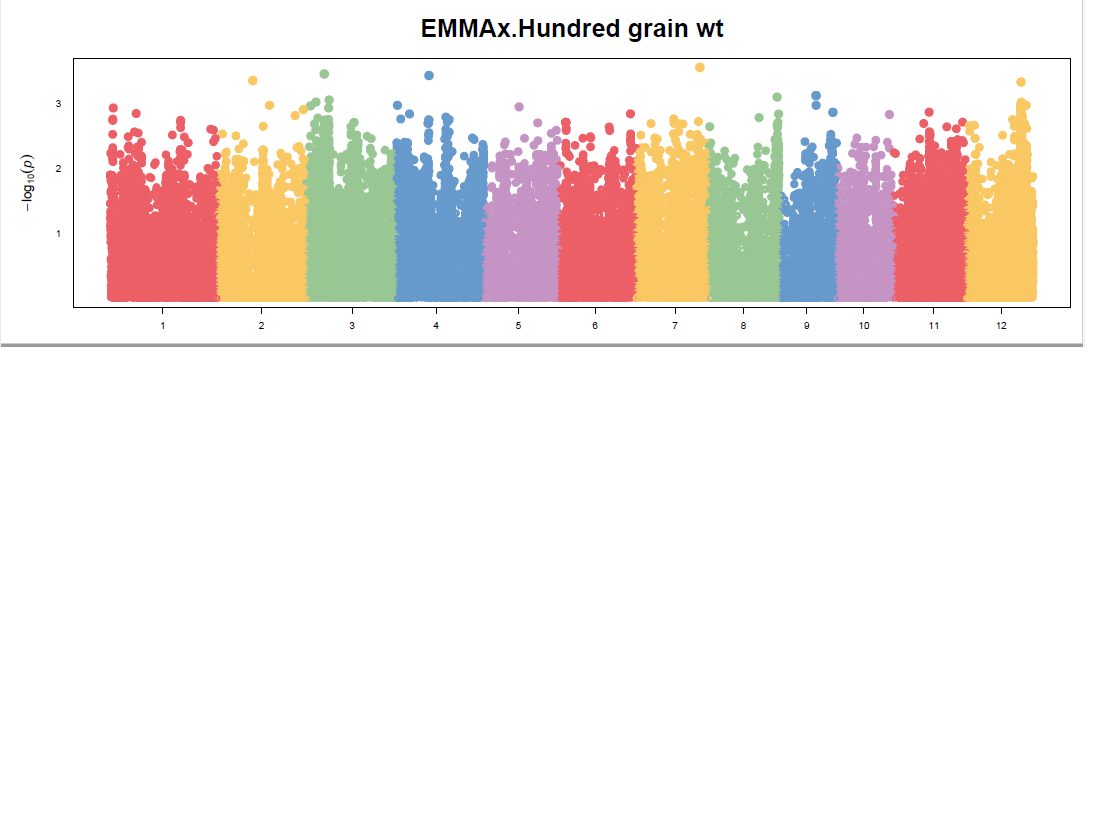


Relative number of filled grains


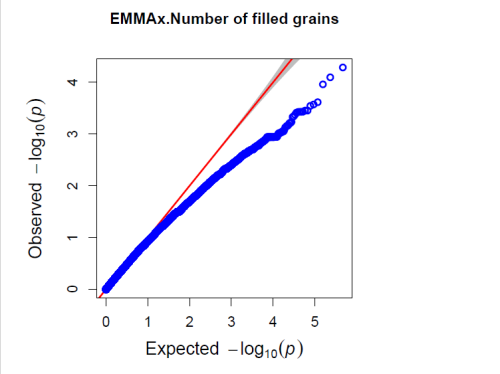


Chromosome


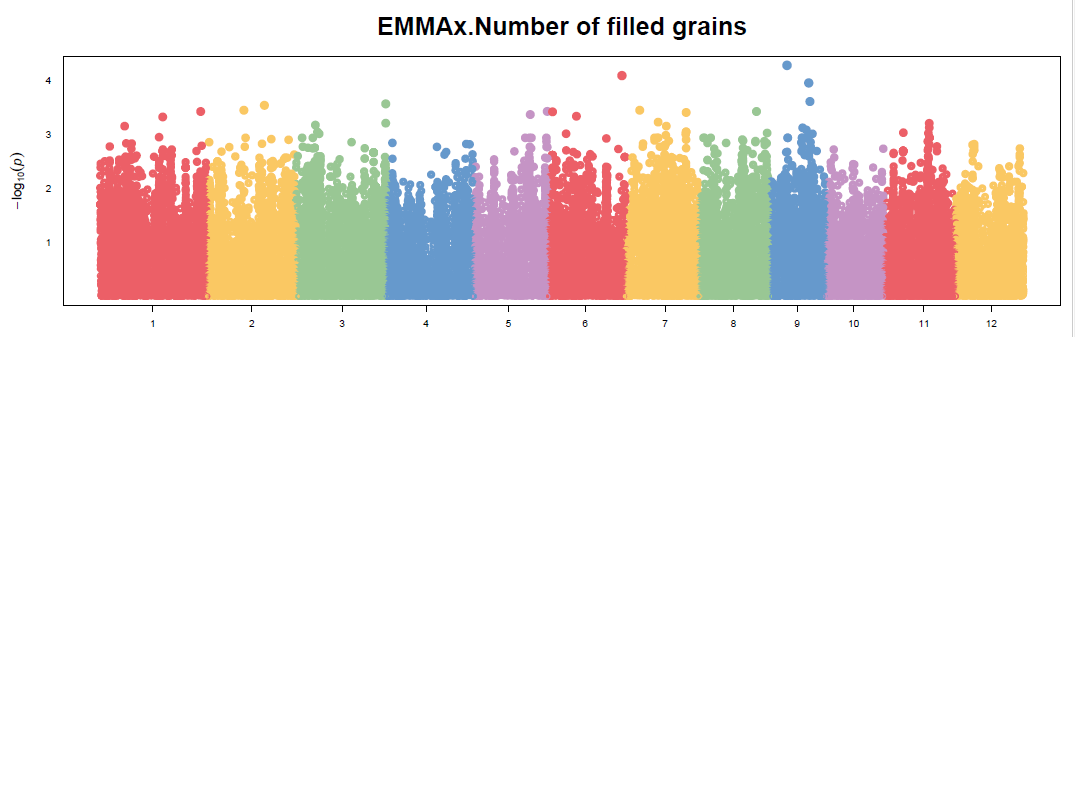


Relative number of unfilled grains


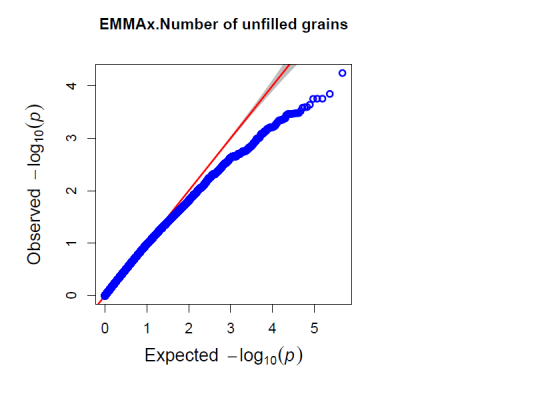


Chromosome


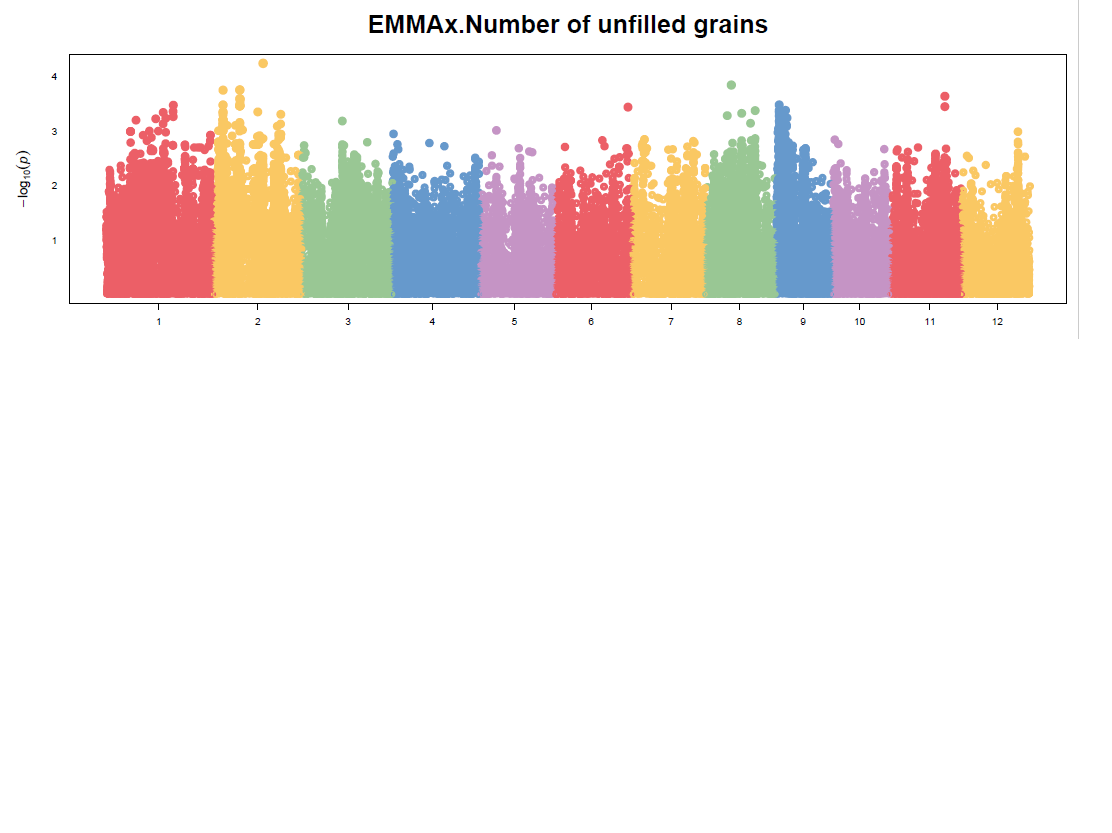


**Figure S6.** Manhattan plot and Quantile-Quantile (QQ) plots of relative grain yield, relative hundred grain weight, relative number of filled grains, relative number of unfilled grains, relative panicle length, relative plant height, relative shoot biomass, relative tiller number, leaf [Na^+^] under salinity, leaf [K^+^] under salinity and leaf Na^+^/K^+^ ratio under salinity using 3KRG 1^st^ batch panel. Negative log_10_- transformed P values from a genome wide scan are plotted against position on each of the 12 chromosomes.

Chromosome


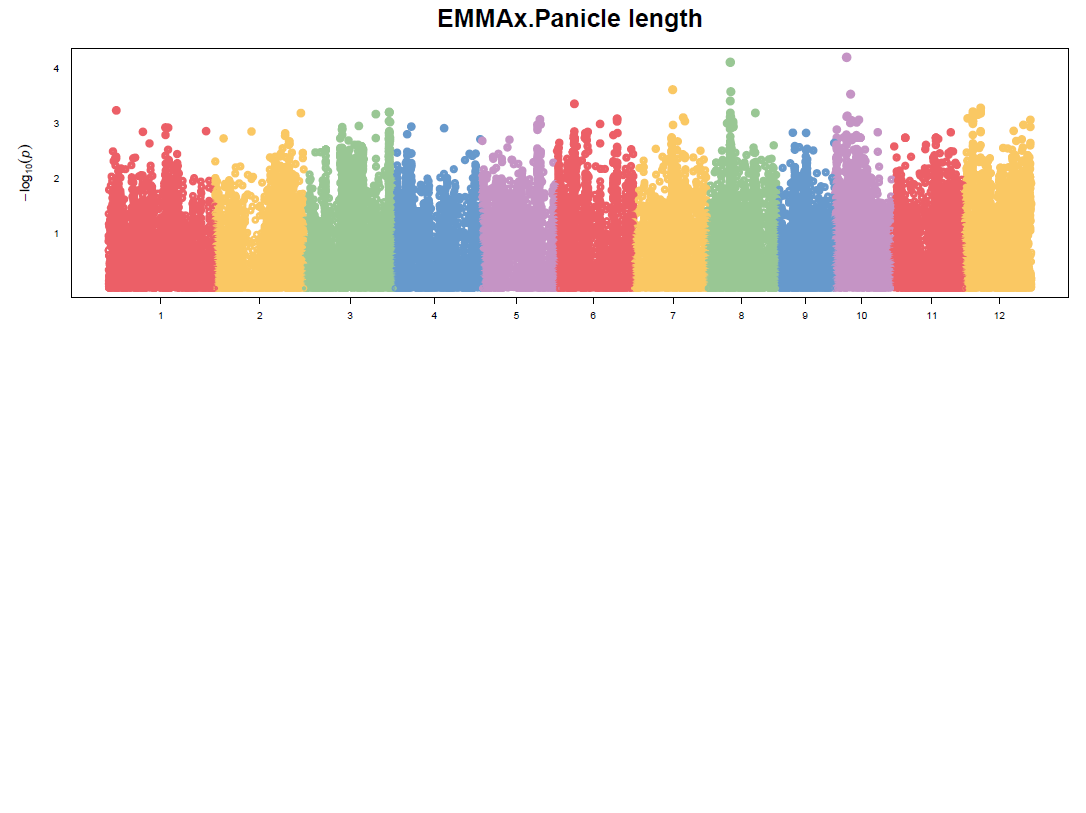


Relative panicle length


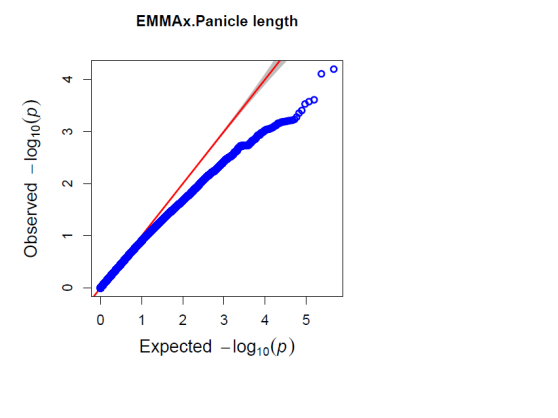


Relative plant height

Chromosome


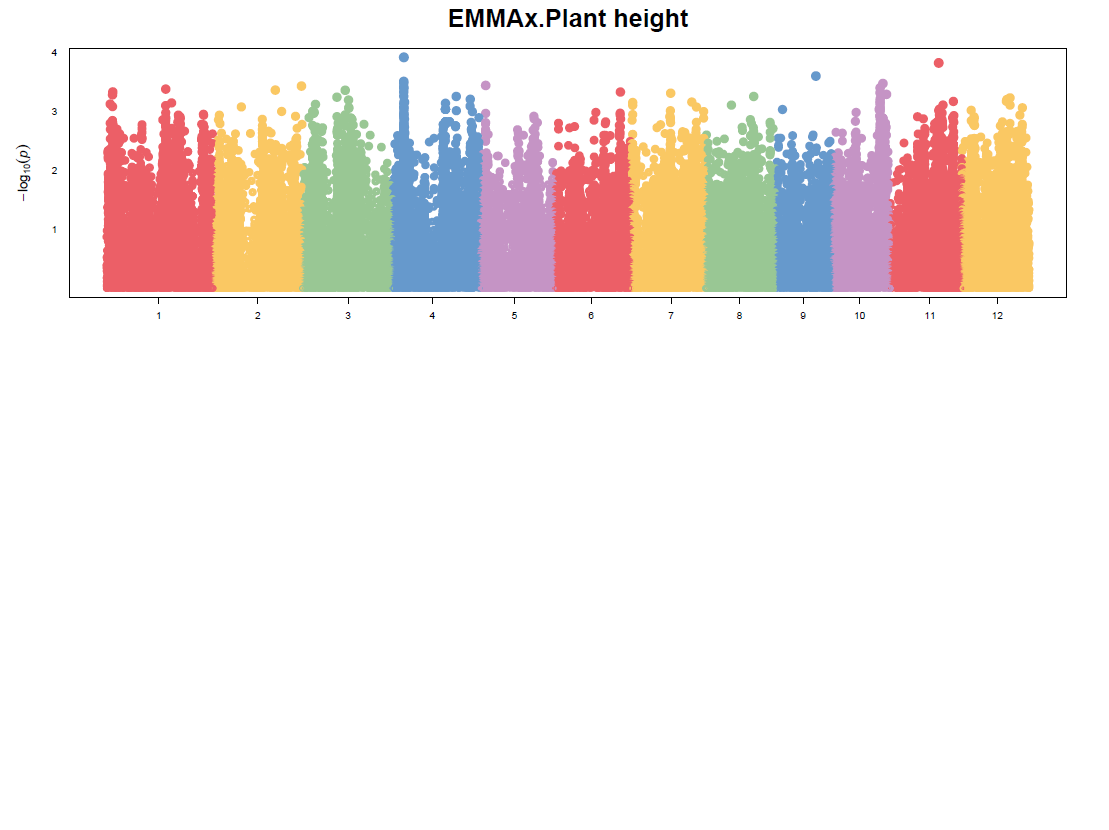


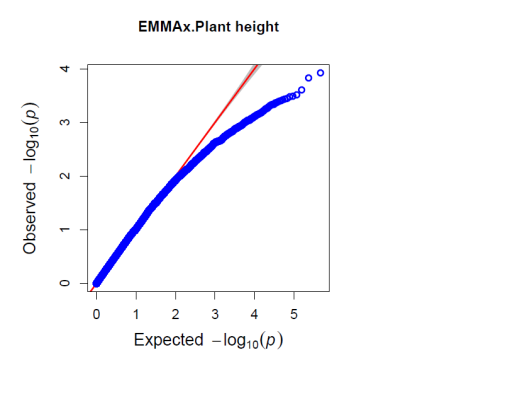


Relative shoot biomass


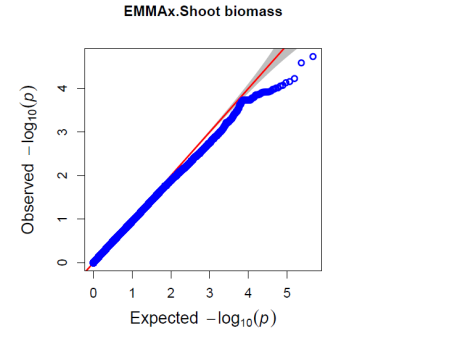


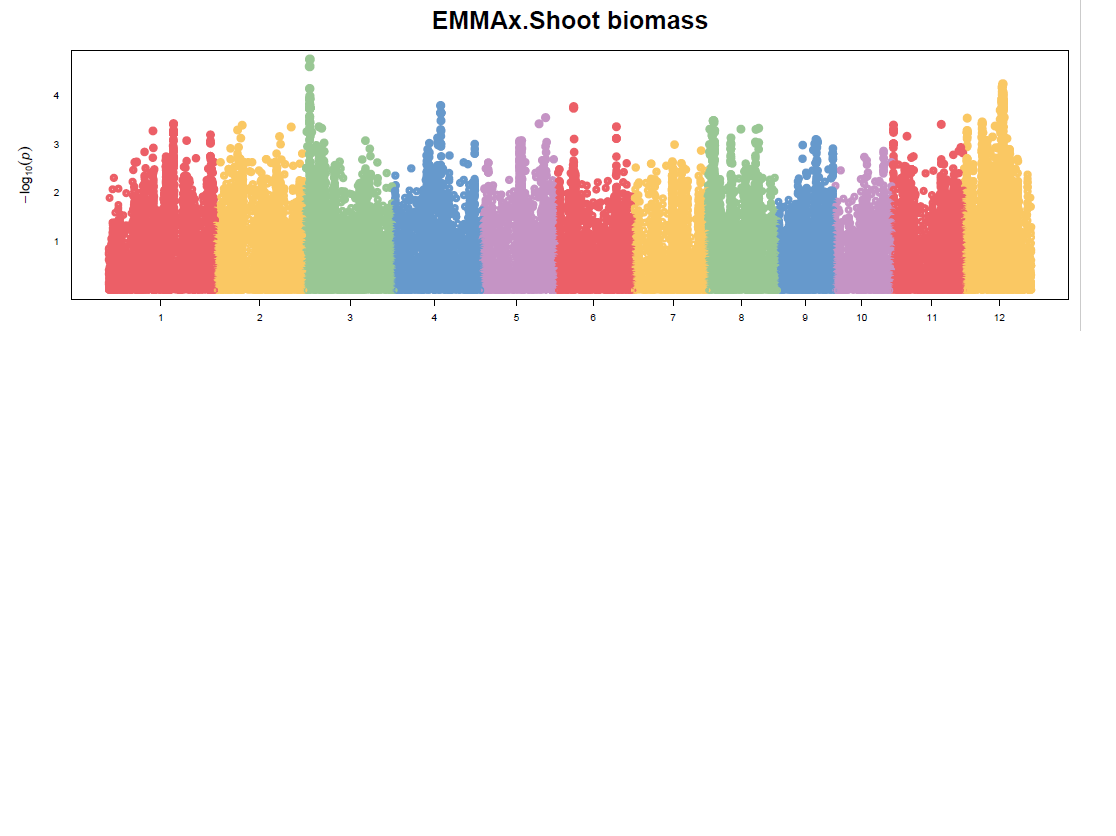


Chromosome

Chromosome

Relative tiller number


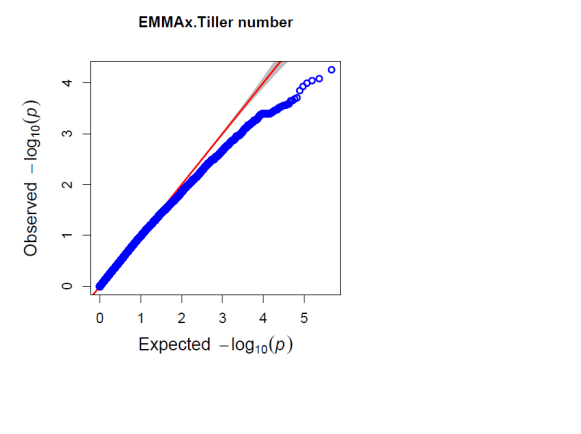


Chromosome


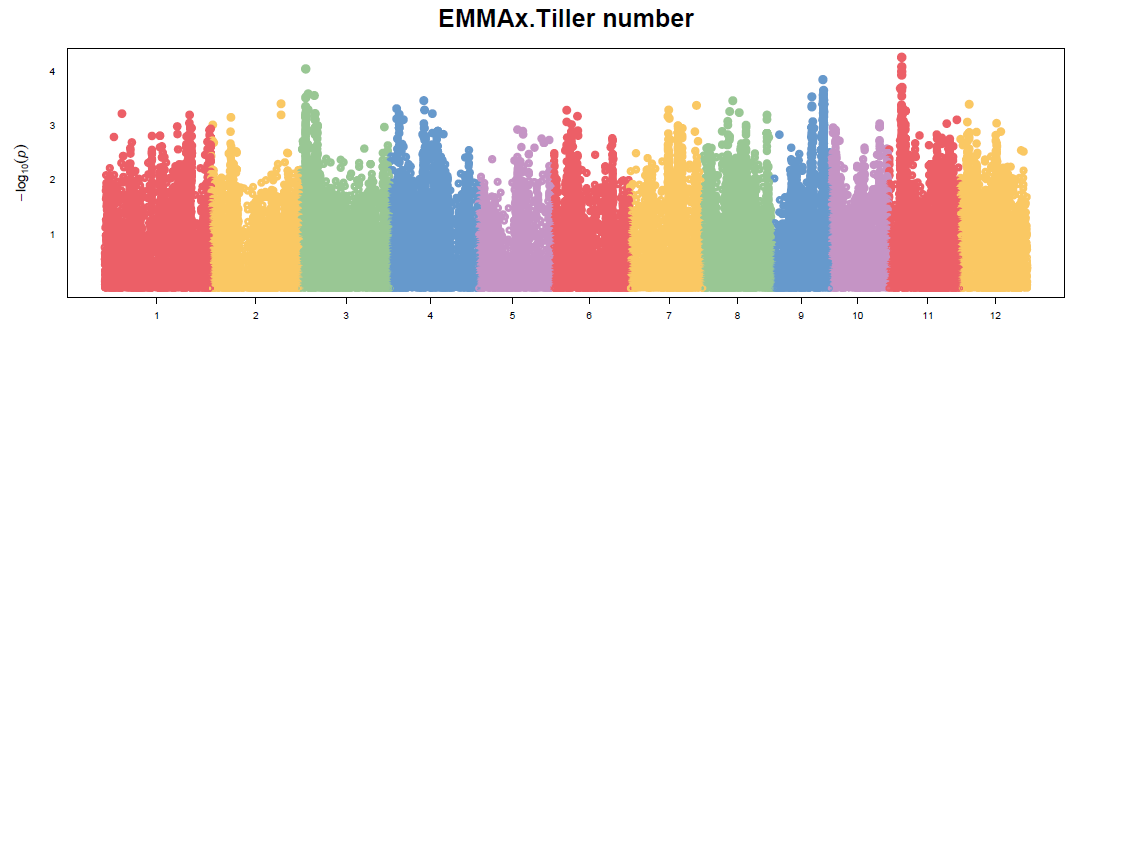


(**Figure S6 continued.)**

Leaf [Na^+]^ under salinity


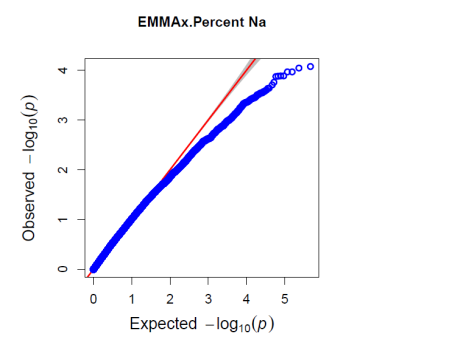


Chromosome


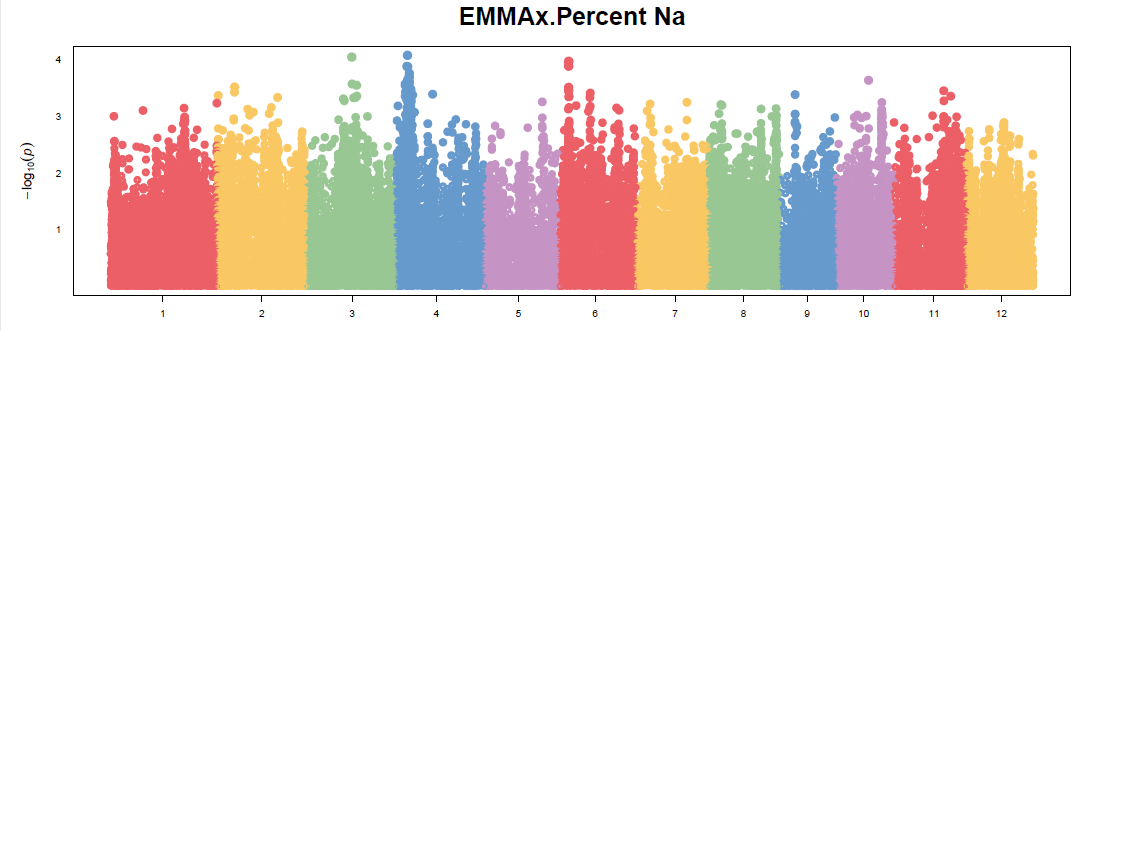


Leaf [K^+^] under salinity


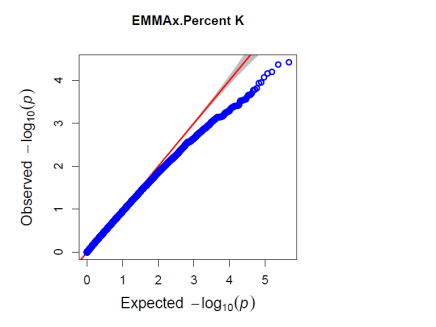


Chromosome


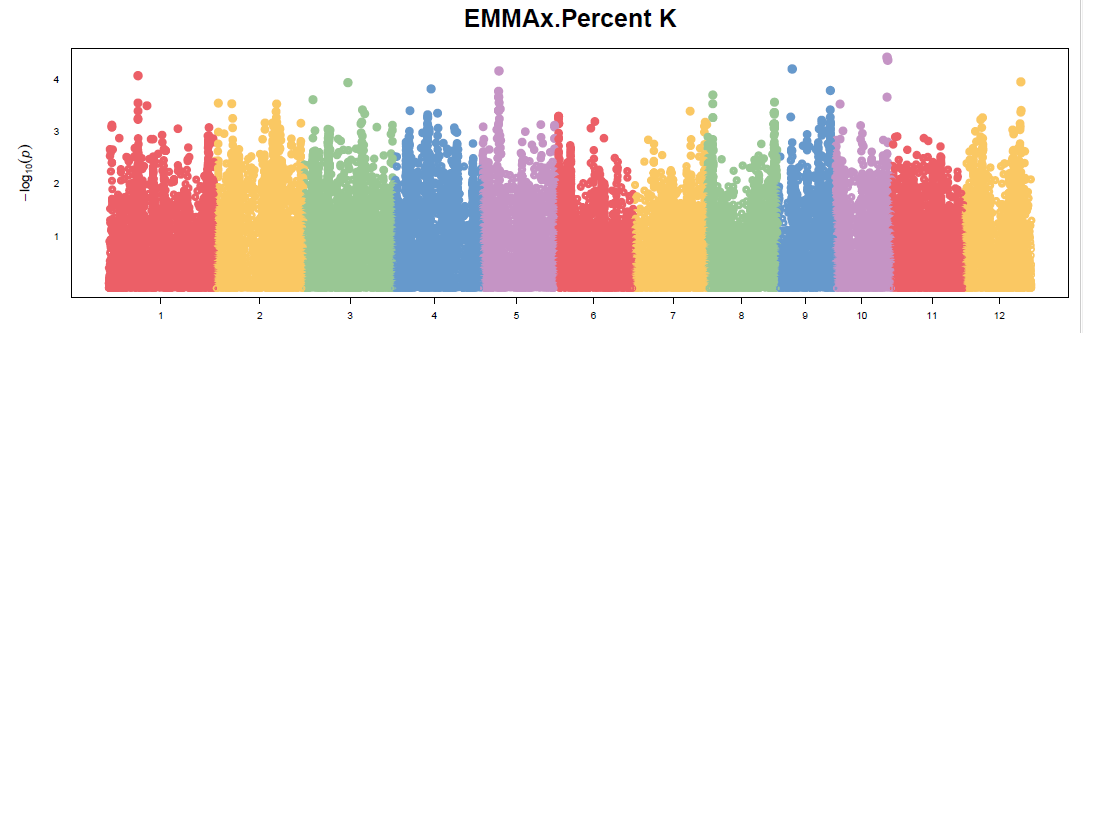


Leaf Na^+^/K^+^ ratio under salinity


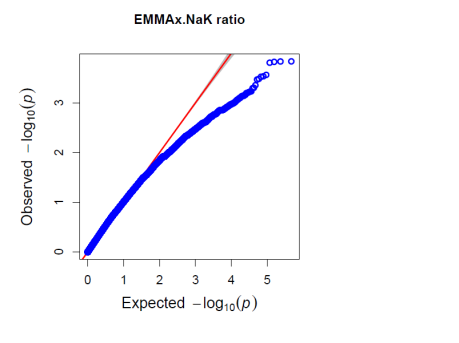


Chromosome


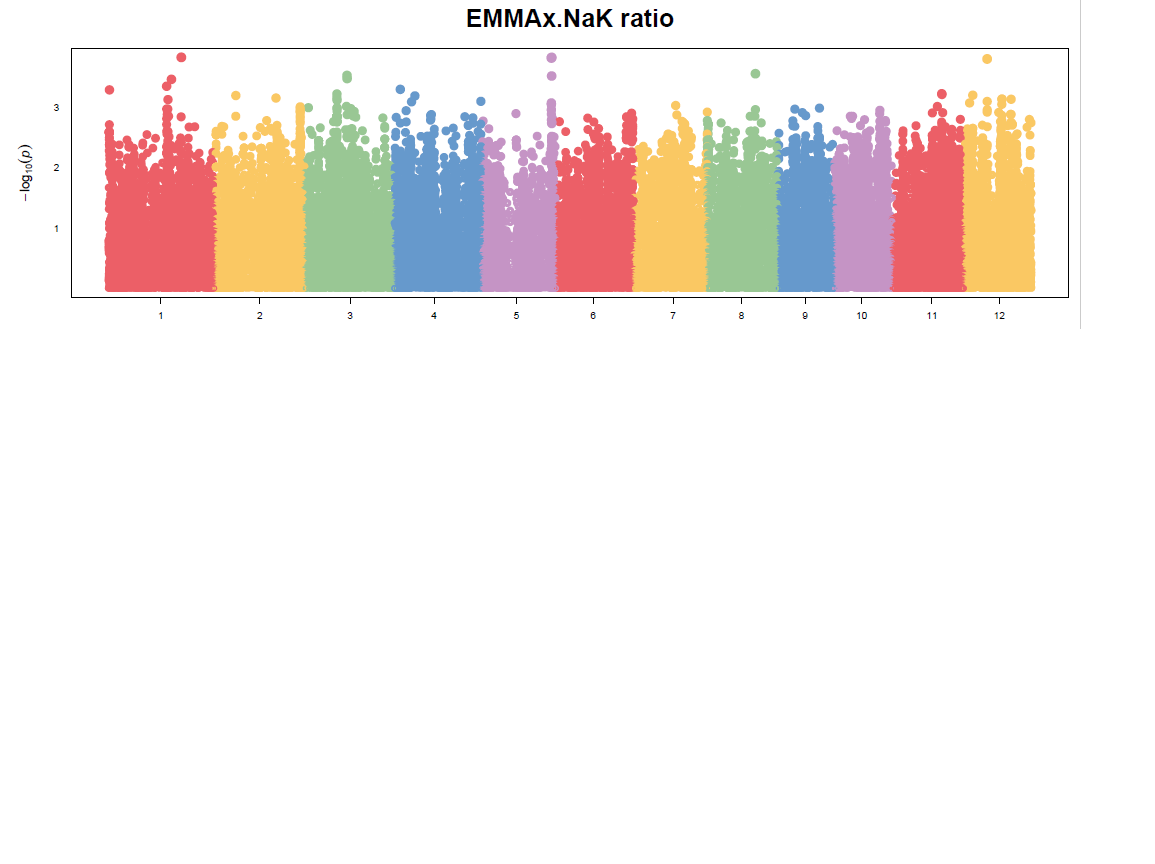


(**Figure S6 continued.)**


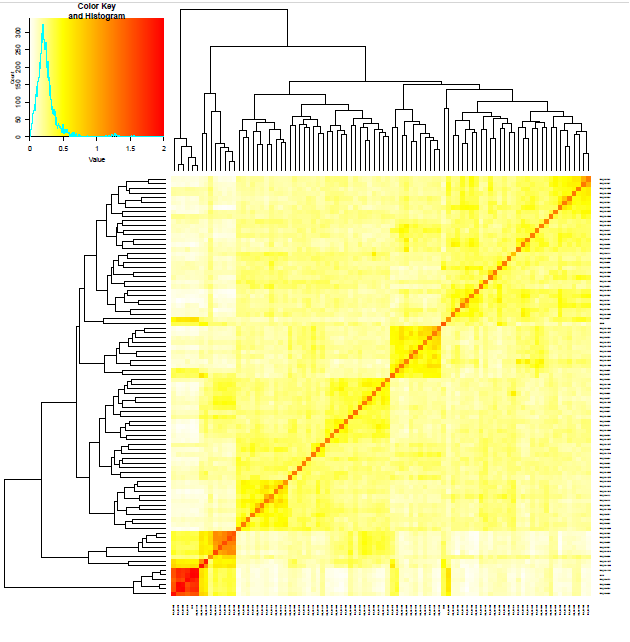

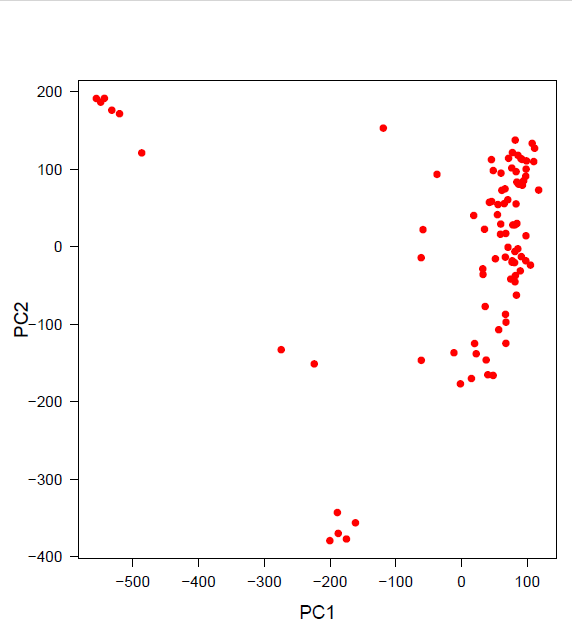


**Figure S7.** Population structure in the 3KRG 2^nd^ batch panel (A) Heatmap of the kinship matrix and (B) Scatterplot of the first two principal components (PC) produced by GAPIT.


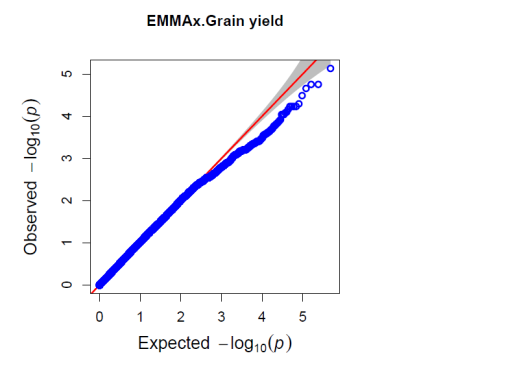


Chromosome


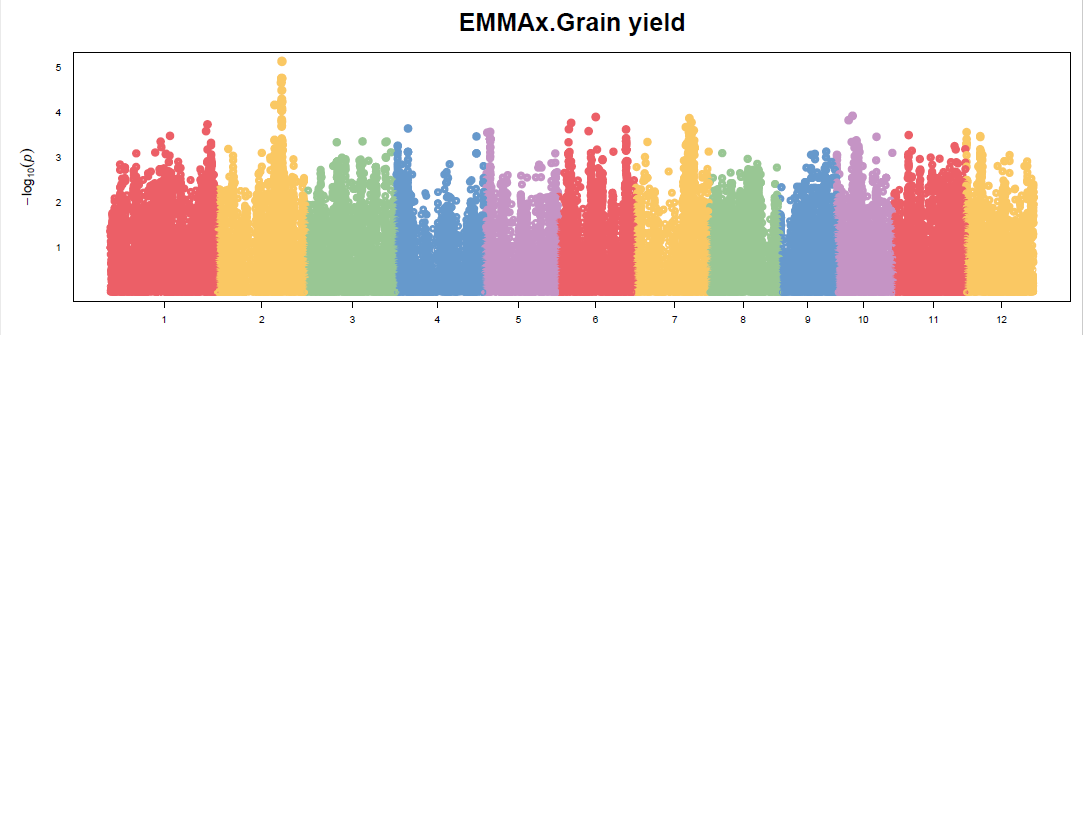


Relative grain yield

Relative hundred grain weight


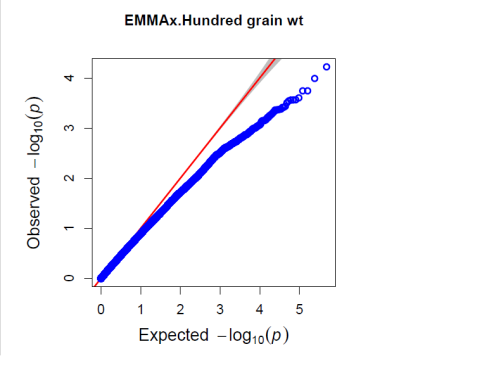


Chromosome


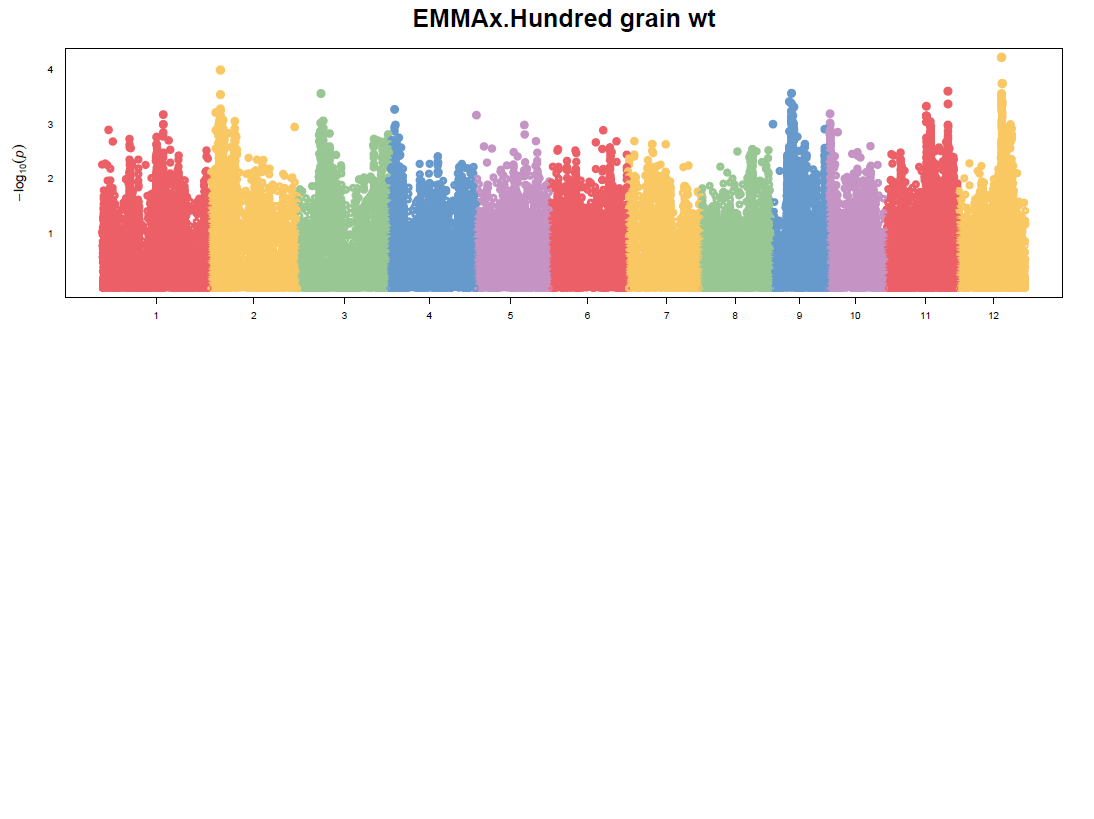


Relative number of filled grains


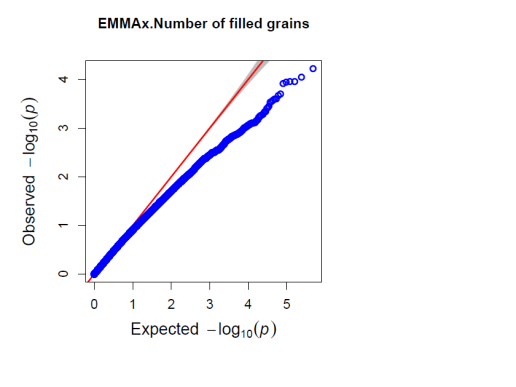


Chromosome


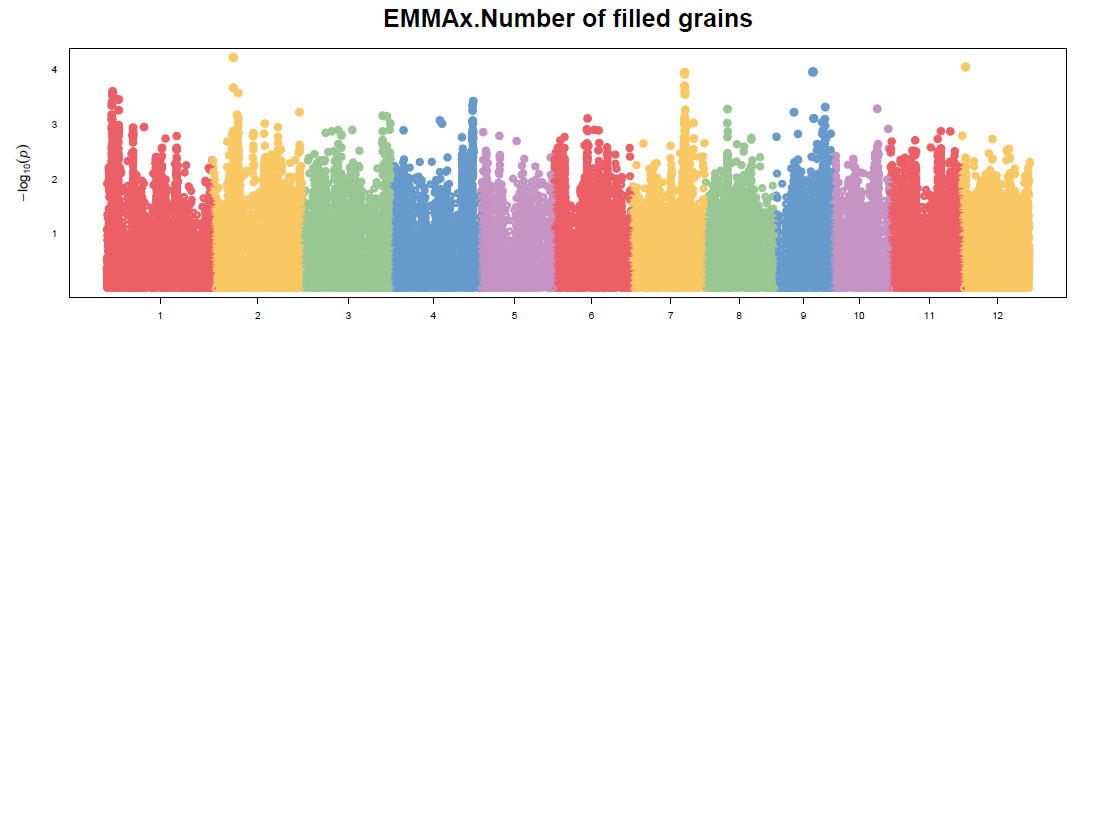


Relative number of unfilled grains


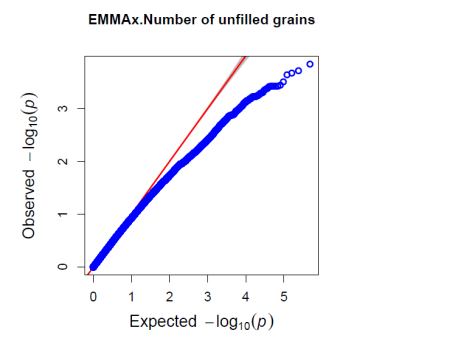


Chromosome


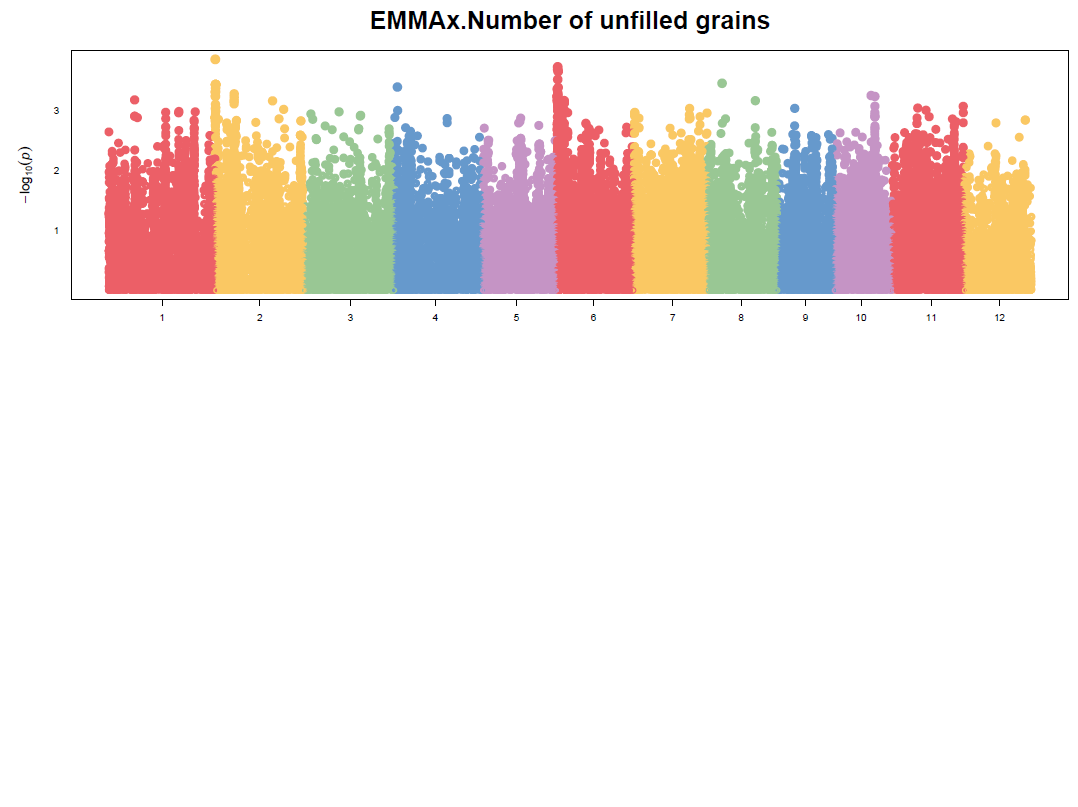


**Figure S8.** Manhattan plot and Quantile-Quantile (QQ) plots of relative grain yield, relative hundred grain weight, relative number of filled grains, relative number of unfilled grains, relative panicle length, relative plant height, relative shoot biomass, relative tiller number, leaf [Na^+^] under salinity, leaf [K^+^] under salinity and leaf Na^+^/K^+^ ratio under salinity using 3KRG 2^nd^ batch panel. Negative log_10_- transformed P values from a genome wide scan are plotted against position on each of the 12 chromosomes.

Relative panicle length

Relative plant height

Relative shoot biomass

Relative tiller number


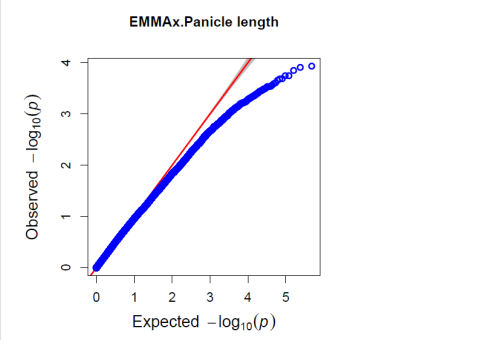


Chromosome


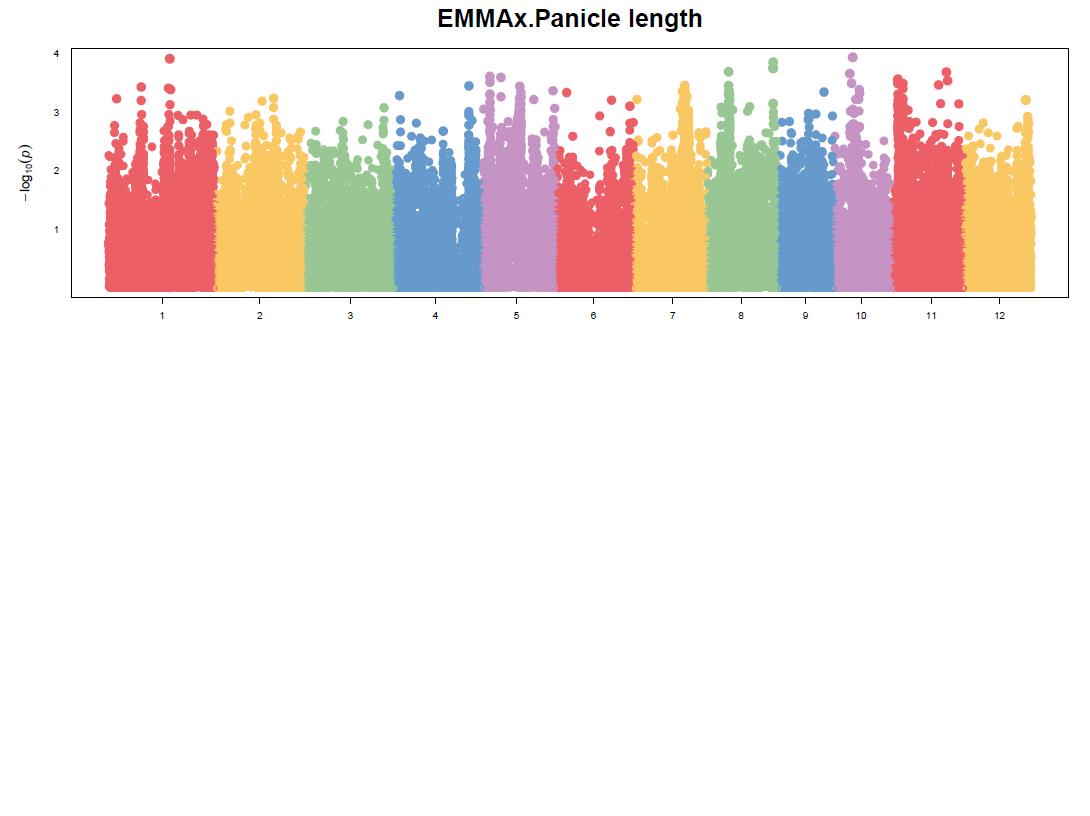


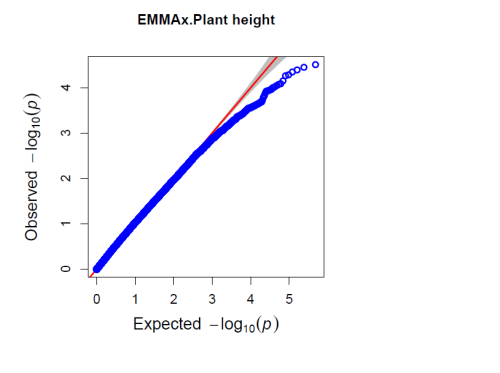


Chromosome


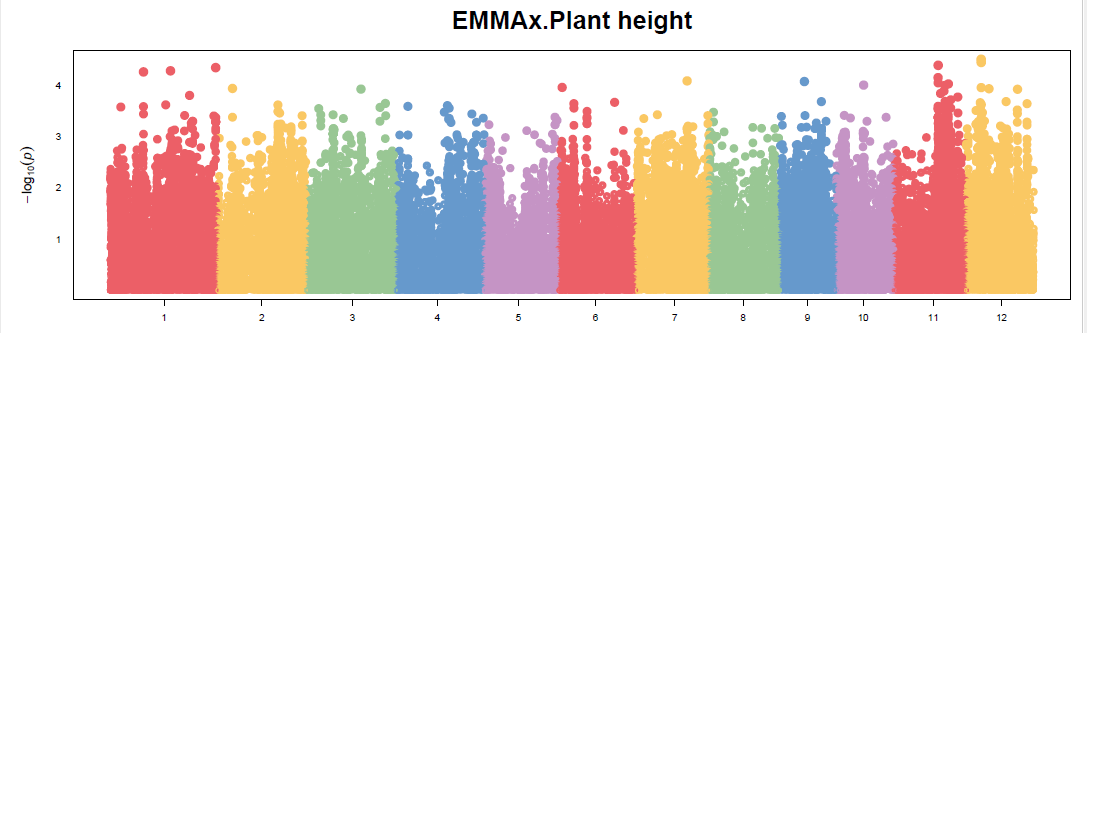


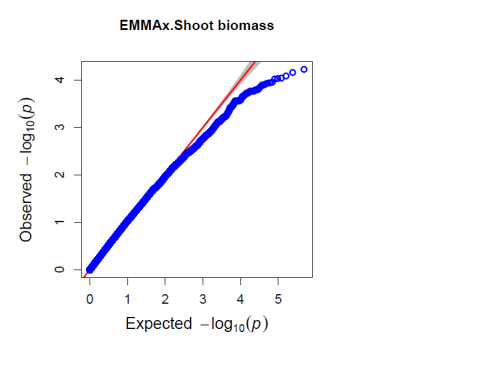


Chromosome


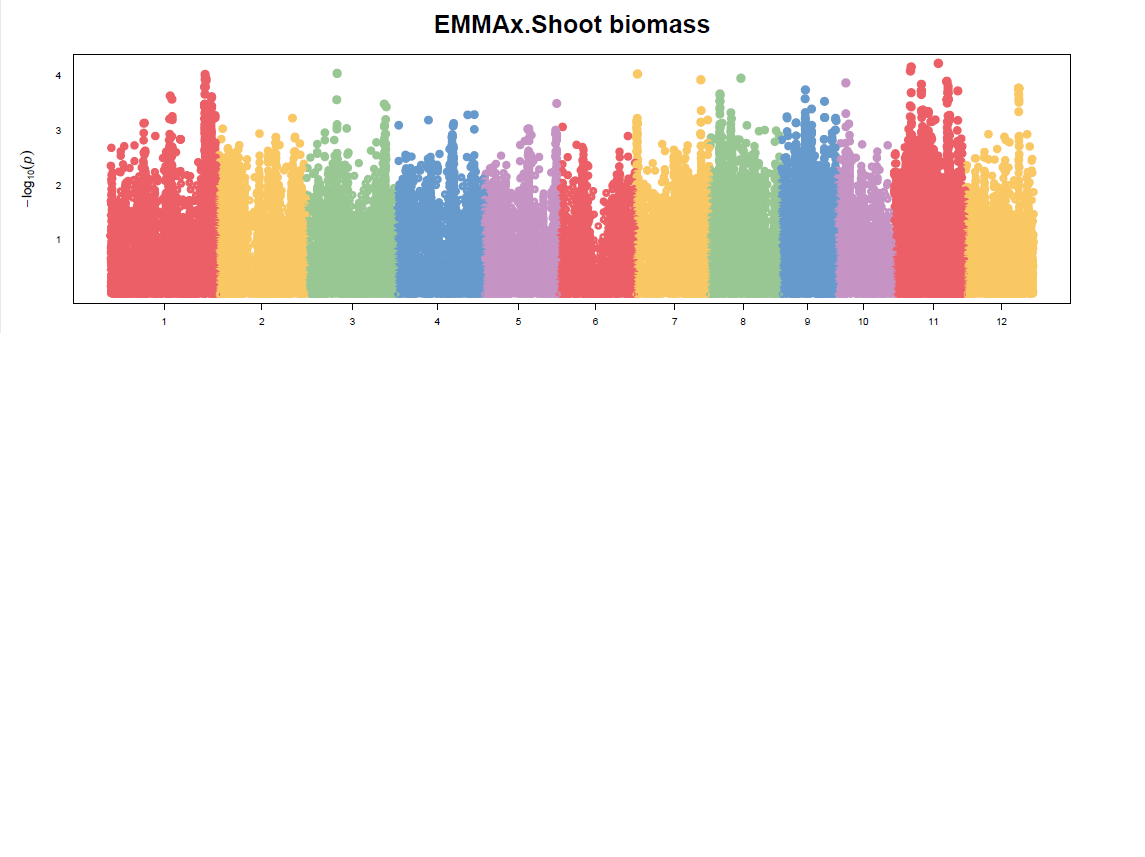


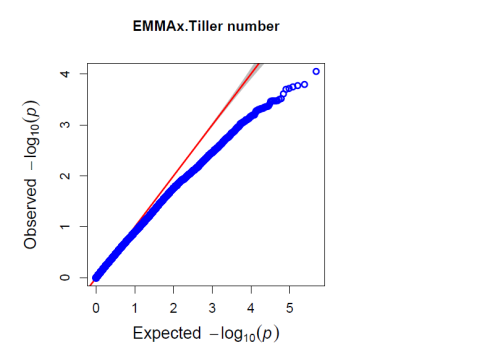


Chromosome


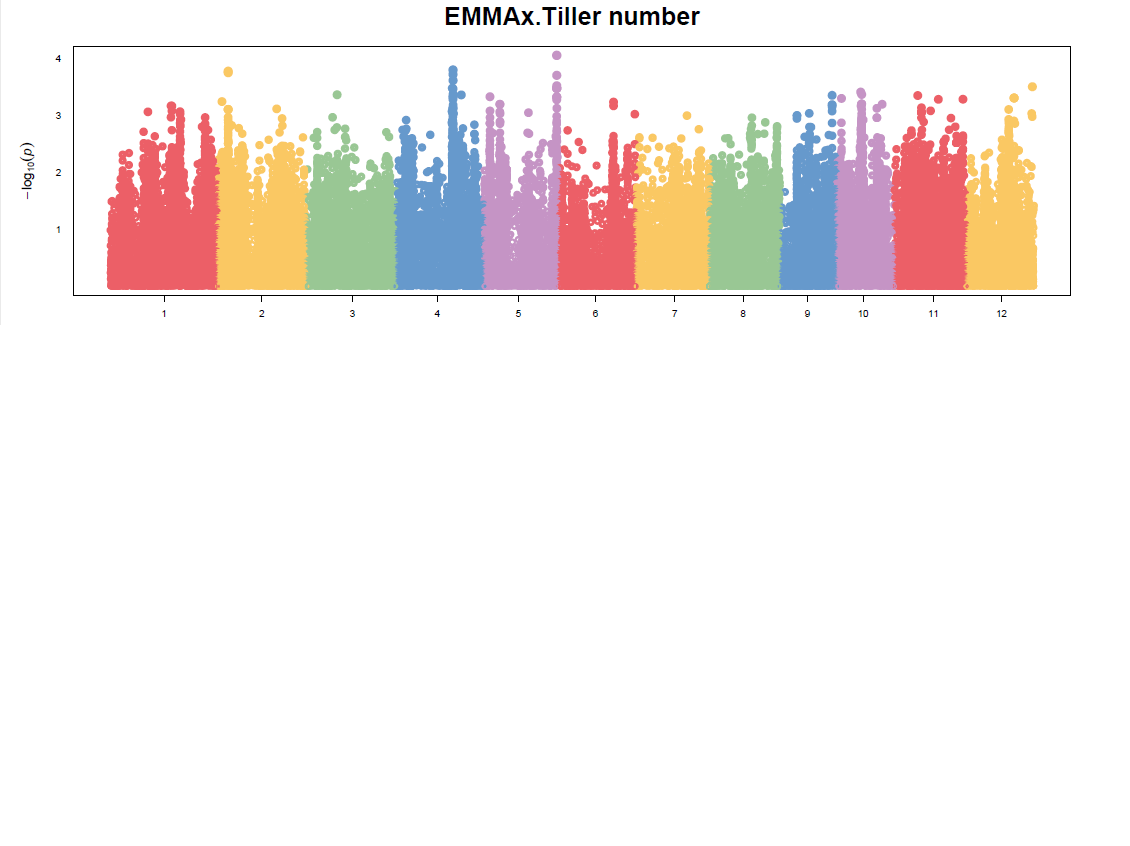


(**Figure S8 continued.)**

Leaf [Na^+]^ under salinity

Leaf Na^+^/K^+^ ratio under salinity

Leaf [K^+^] under salinity


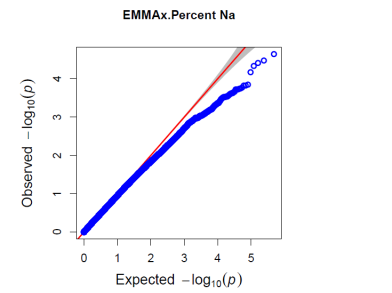


Chromosome


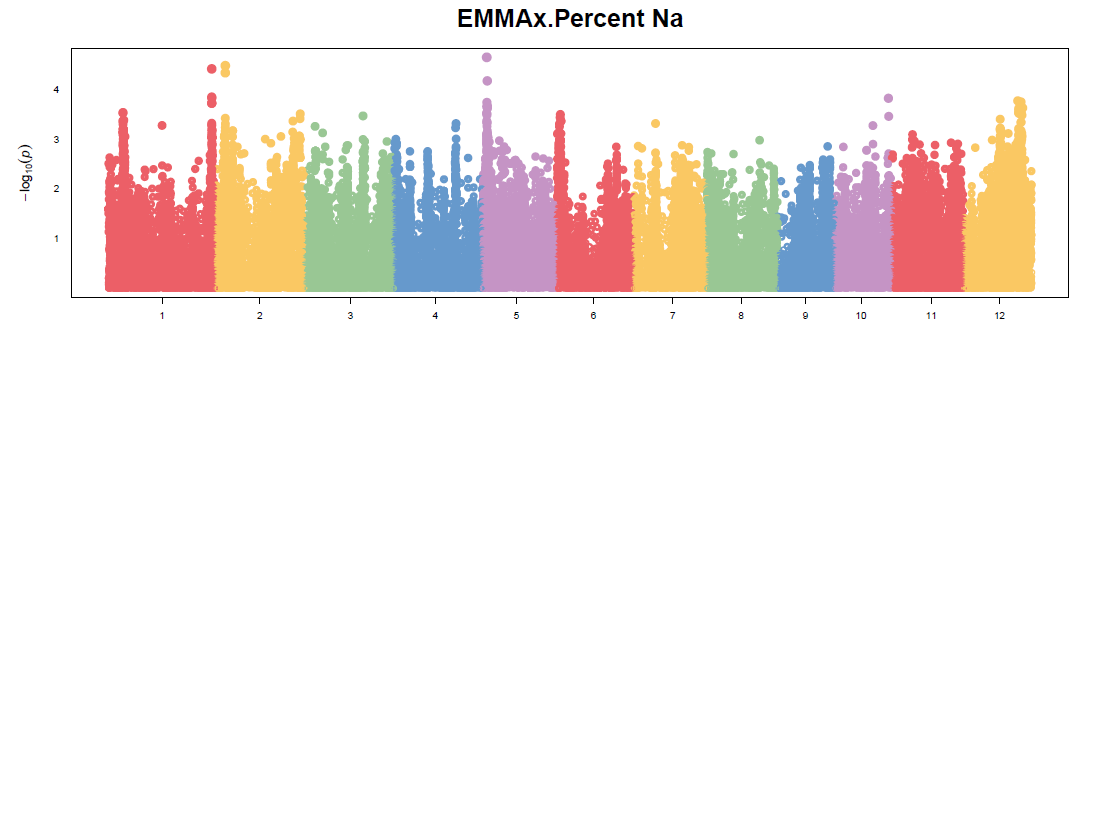


Chromosome


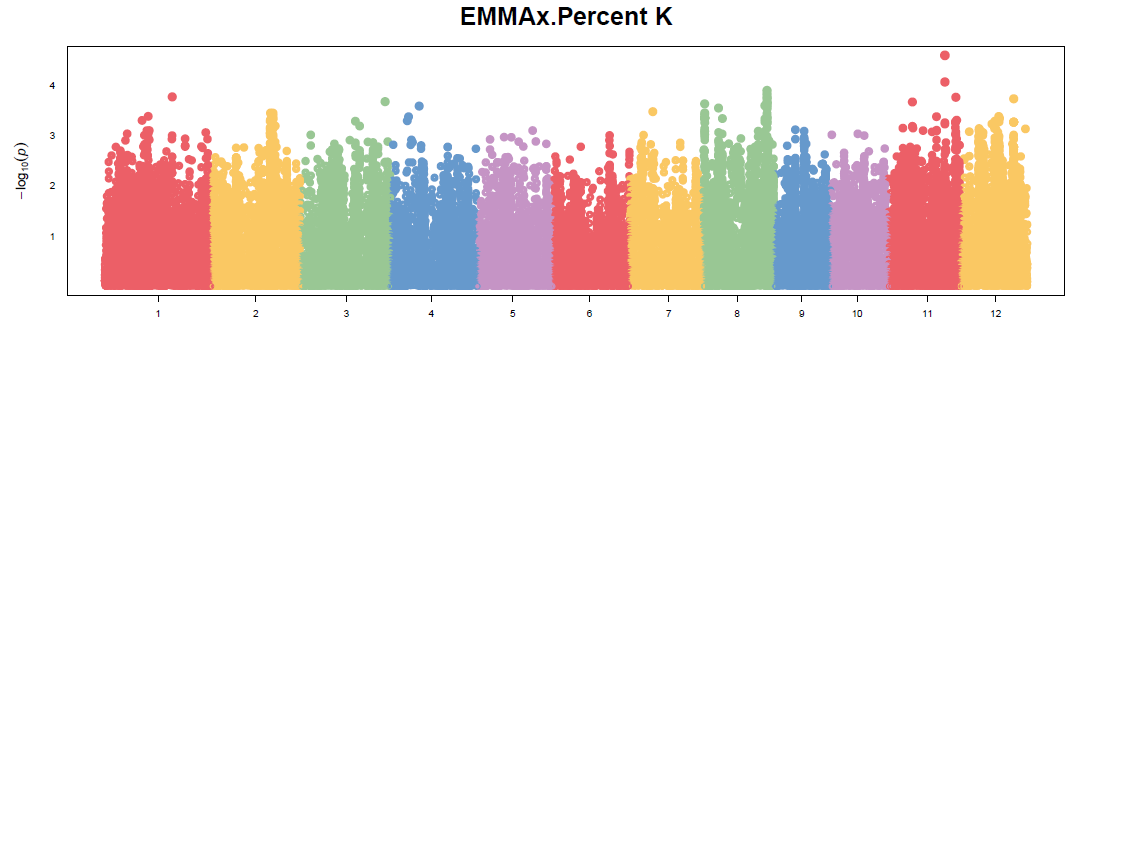


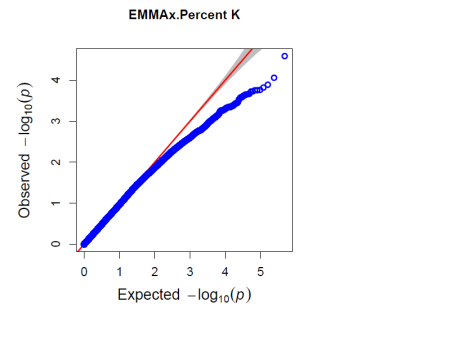


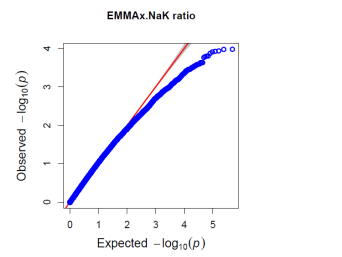


Chromosome


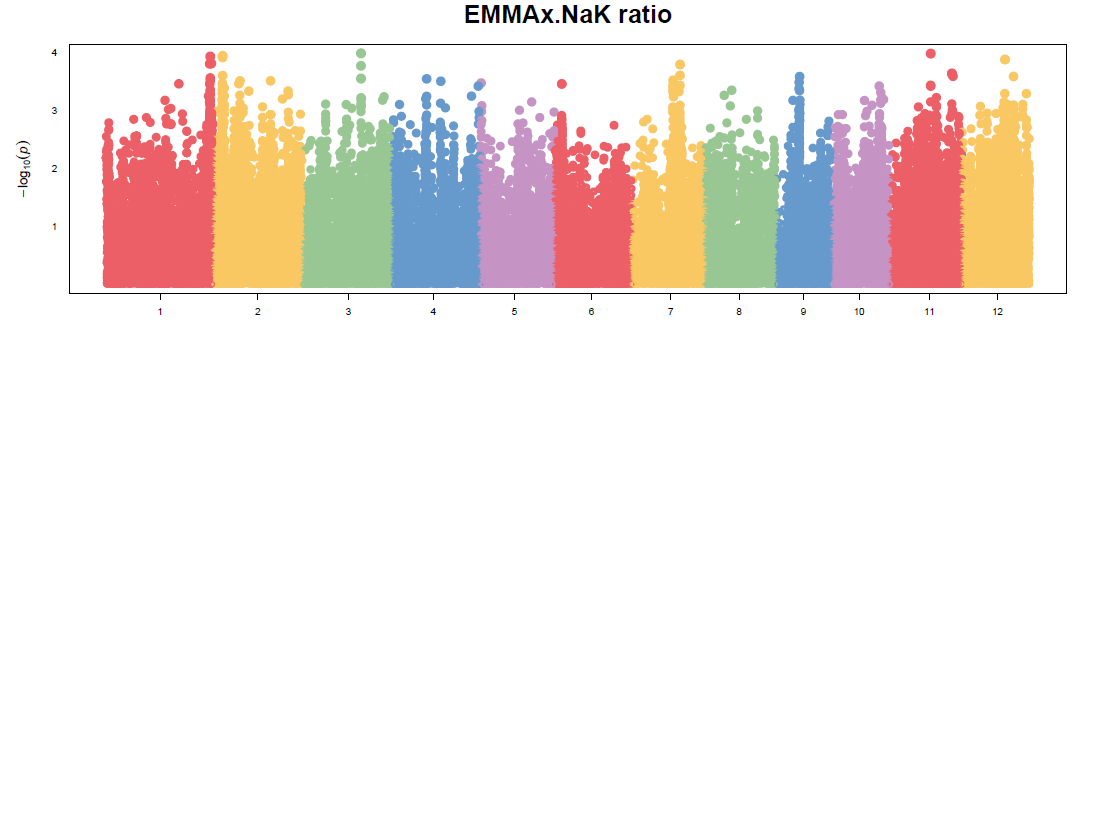


(**Figure S8 continued.)**

A


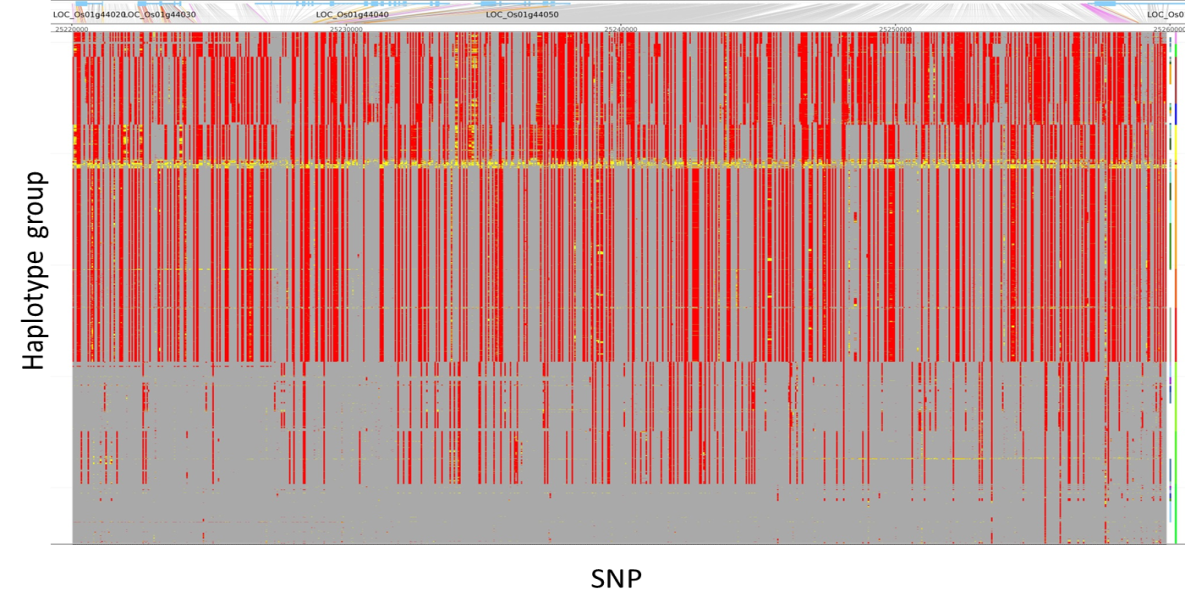

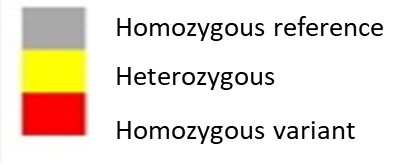


B

RDP1

3KRG 1^st^ batch

3KRG 2^nd^ batch batch


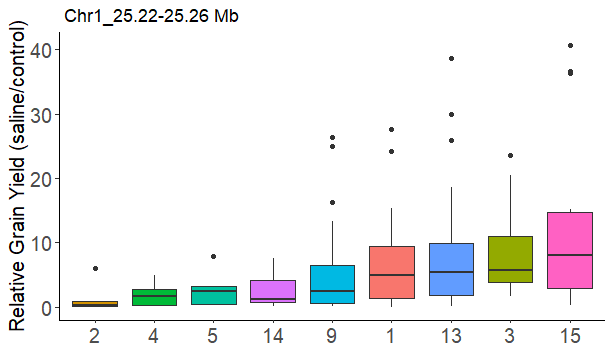

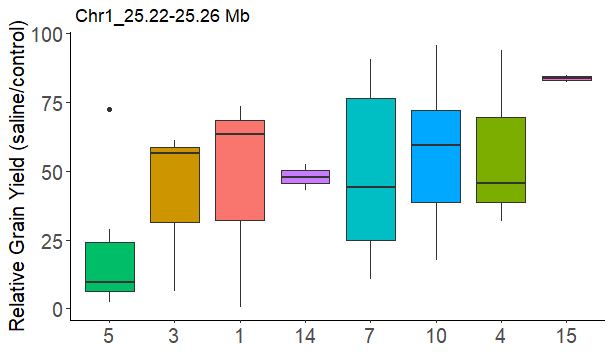

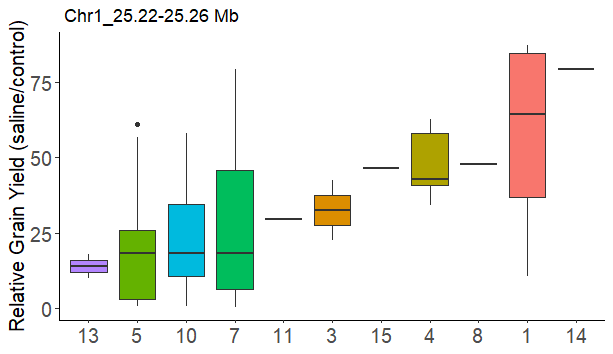


**Figure S9.** (A) Haploview of Chr1_25.22-25.26 Mb and (B) Boxplot of the haplotype groupings for the 40kb subregion inside the 100 kb window on Chr 1 (the position indicated after underscore denotes the start of the locus in Mb that showed a high number of co-locating GWAS peaks for relative grain yield.

A


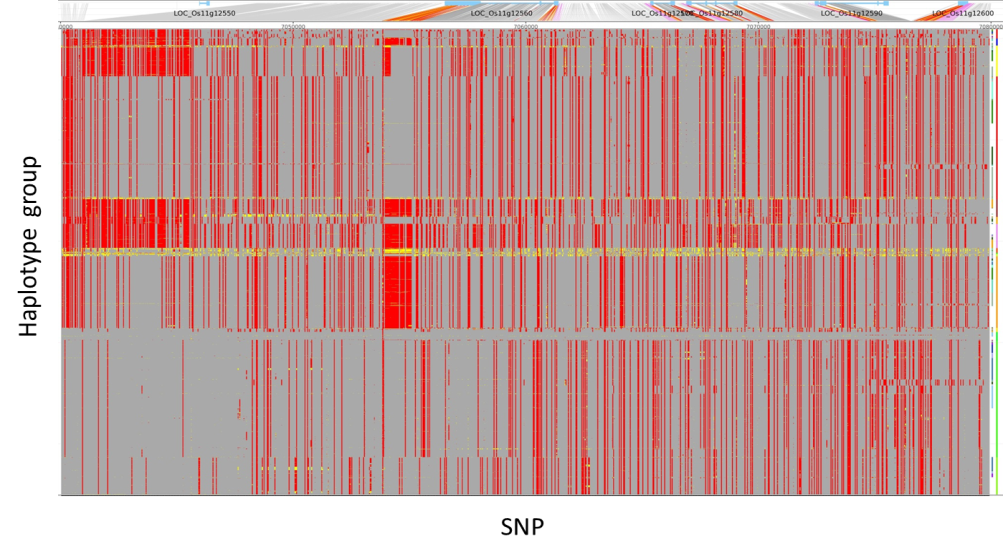

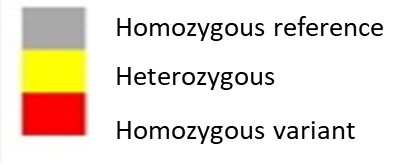


B

RDP1

3KRG 1^st^ batch

3KRG 2^nd^ batch


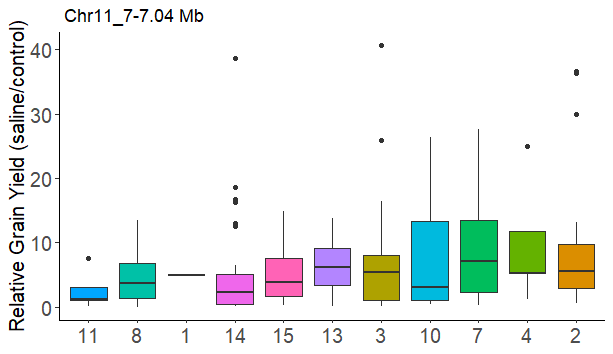

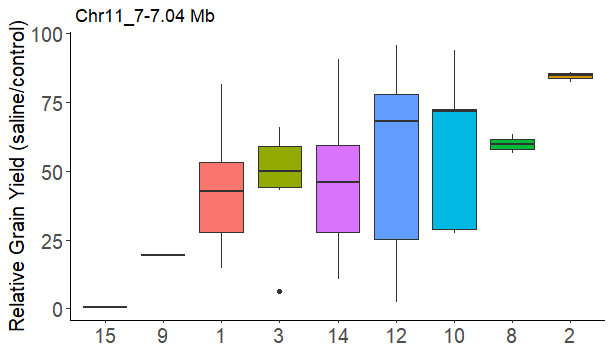

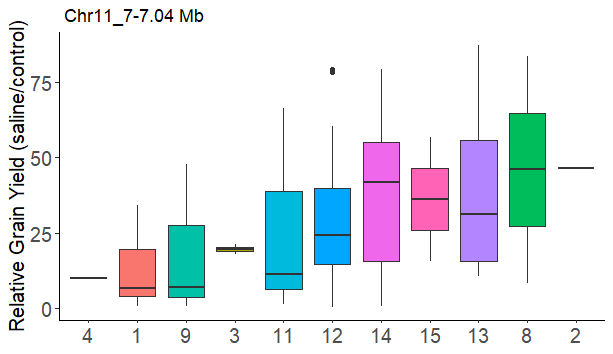


**Figure S10.** (A) Haploview of Chr11_7-7.04Mb and (B) Boxplot of the haplotype groupings for the 40kb subregion inside the 100 kb window on Chr 11 (the position indicated after underscore denotes the start of the locus in Mb that showed a high number of co-locating GWAS peaks for relative grain yield.


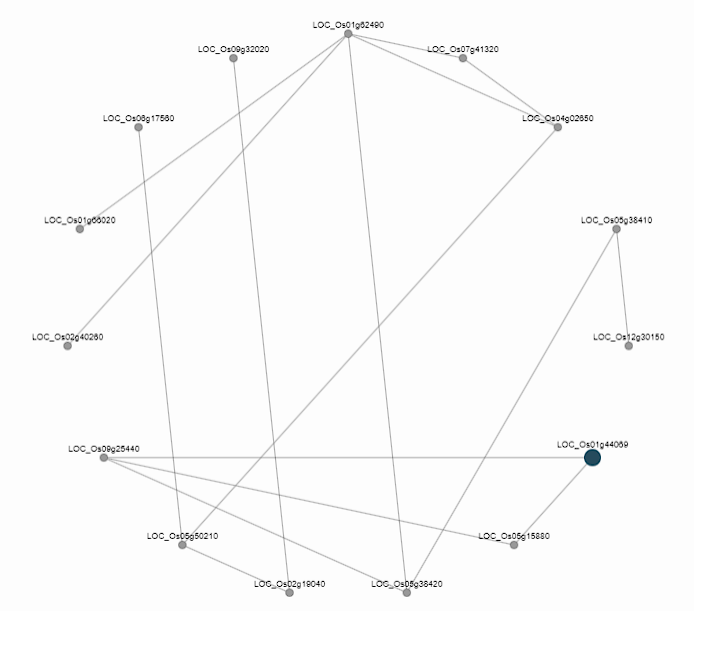


**Figure S11.** Co-expression network analysis of Chr1_25.22-25.26 GWAS interval showing enriched ClusterONE module 2580 (adj. p-value = 2.442115e-02) of RiceNet v2 in RicePilaf. The larger, dark blue nodes refer to the genes implicated by the GWAS interval and the lines connecting them represent relationships between genes. The more connected the genes are, the more important the gene in the interaction network.
